# Discovery of novel 1,3,4-thiadiazole derivatives as potential dual EGFR/VEGFR inhibitors with activity against breast, colon, and liver cancer cells: biological evaluation and molecular docking investigation

**DOI:** 10.1039/d6ra07414f

**Published:** 2026-11-11

**Authors:** Amany Belal, Mohammed A. S. Abourehab, Reem I. Alsantali, Ahmad J. Obaidullah, Randa Erfan, Yasmine Elwan, Ahmed A. Gaber, Ahmed B. M. Mehany, Rehab Sabour, Walid E. Elgammal

**Affiliations:** a Department of Pharmaceutical Chemistry, College of Pharmacy, Taif University P. O. Box 11099 Taif 21944 Saudi Arabia; b Pharmaceutical Sciences Department, College of Pharmacy, Umm Al-Qura University Makkah 21955 Saudi Arabia; c Department of Pharmaceutical Chemistry, College of Pharmacy, King Saud University P. O. Box 2457 Riyadh 11451 Saudi Arabia; d Department of Medical Biochemistry and Molecular Biology, Faculty of Medicine, Cairo University Cairo Egypt; e Department of Pharmaceutical Organic Chemistry, Faculty of Pharmacy (Boys), Al-Azhar University Cairo 11884 Egypt; f Zoology Department, Faculty of Science (Boys), Al-Azhar University Nasr City 11884 Cairo Egypt; g Department of Pharmaceutical Chemistry, Faculty of Pharmacy (Girls), Al-Azhar University Cairo Egypt; h Chemistry Department, Faculty of Science (Boys), Al-Azhar University Nasr City 11884 Cairo Egypt walidebaied.sci85@azhar.edu.eg +201025267111

## Abstract

New derivatives of the 1,3,4-thiadiazole scaffold were synthesized and evaluated for their cytotoxic effects on breast (MCF-7), hepatic (HepG-2), and colorectal (HCT-116) carcinomas. The most active compounds (7, 8, 15, and 16) exhibited substantial, broad-spectrum anticancer efficacy, with IC_50_ values ranging from 1.58–4.27 µM relative to doxorubicin. To explore their potential modulatory capacity on receptor tyrosine kinases (RTKs), compounds 7, 8, 15, and 16 were evaluated for enzymatic efficacy against VEGFR-2, and the most active hits, 7 and 15, were further assessed against EGFR. The findings suggest a possible VEGFR/EGFR-mediated inhibition of cancer cell proliferation, with compounds 7 and 15 showing enzymatic inhibitory activity, displaying IC_50_ values of 0.42 and 0.23 µM for EGFR and 0.17 and 0.16 µM for VEGFR-2, respectively. Furthermore, both new hits demonstrated reduced cytotoxicity towards normal WI-38 cells, signifying favorable safety profiles. Molecular docking investigations of the active compounds within the VEGFR-2 and EGFR ATP-binding sites supported these *in vitro* assay results. Overall, these findings point to a potential anticancer activity and dual EGFR/VEGFR inhibitors for these compounds.

## Abstract 2

New derivatives of the 1,3,4-thiadiazole-quinazoline-4(3*H*)-one scaffold were synthesized and evaluated for their cytotoxic effects on breast (MCF-7), hepatic (HepG-2), and colorectal (HCT-116) carcinomas.

## Graphical abstract

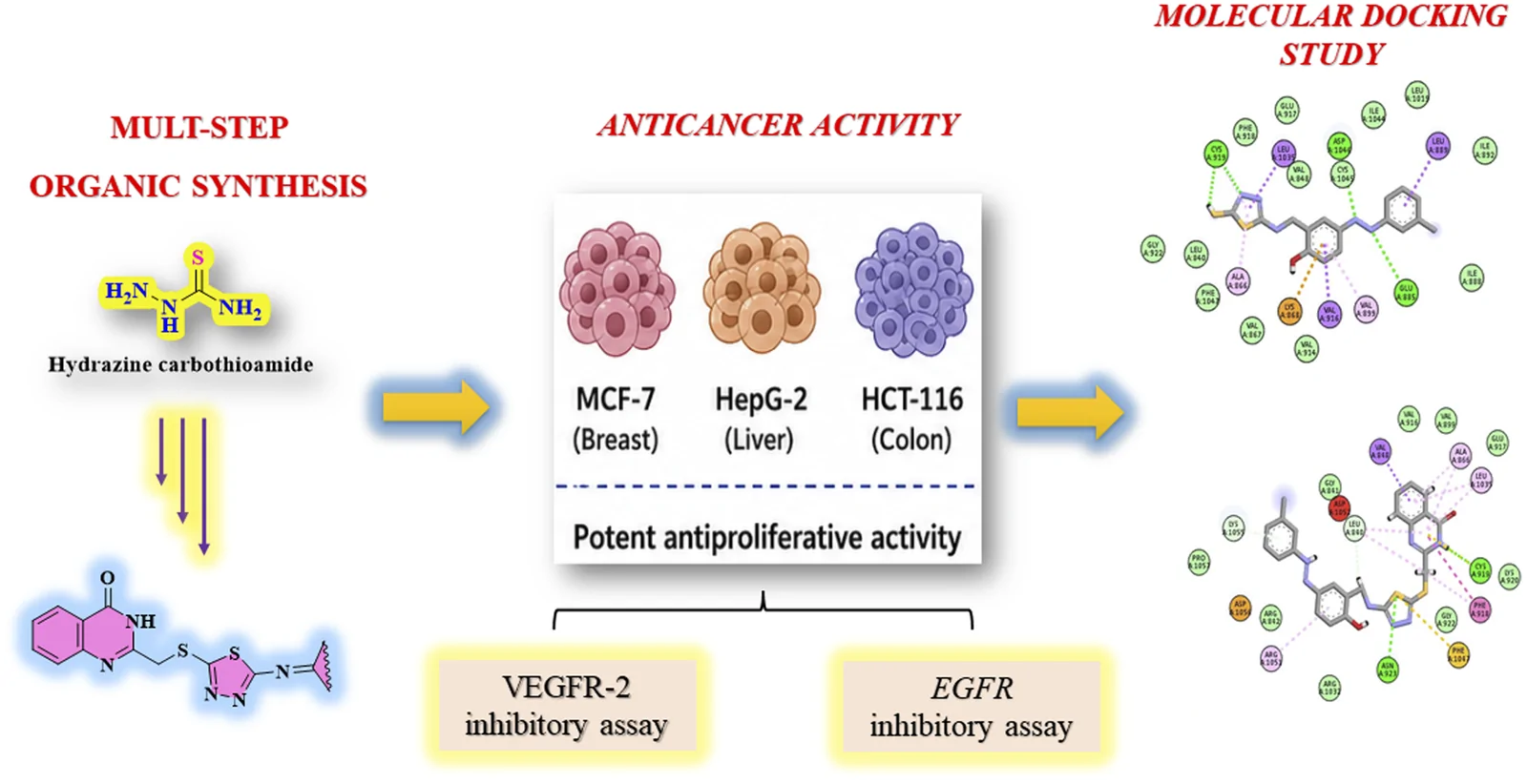

## 1. Introduction

The quest for novel inhibitors targeting receptor tyrosine kinases (RTKs) continues to be a prominent area of research in the field of anticancer investigations.^1^ The reason for this is that the excessive production of specific receptor proteins linked to RTKs results in aberrant cell proliferation and the development of cancer; hence, it is essential to discover new drugs that can inhibit RTK signaling. A well-studied pathway in this domain is the vascular endothelial growth factor (VEGF) and its cell surface receptor, VEGFR-2, because of their crucial role in angiogenesis, a vital mechanism for the survival and expansion of cancer cells.^2,3^ VEGFR-2 is mostly located in endothelial cells, and it is activated when it binds to VEGF and experiences autophosphorylation of its intracellular kinase domains. The C-terminal and N-terminal lobes of protein kinases contribute residues to the area of VEGFR-2 that actively binds adenosine triphosphate (ATP), which is positioned in the space between the two lobes.^4^ Located at the lobe, the C-terminal contains an activation loop that begins with a conserved aspartate–phenylalanine–glycine (DFG) motif.^5^ There are three classes of VEGFR-2 inhibitors, including Type I, Type II, and Type III inhibitors. Type II inhibitors preserve the inactive conformation of VEGFR2 kinase, which includes the DFG motif in the DFG-out state. These inhibitors also establish a hydrophobic allosteric pocket adjacent to the ATP-binding site.^6^ Through suppression of VEGFR-2, this triggers signaling pathways that lead to the development of blood vessels towards the tumor cells.^7^ Hence, inhibiting the activity of VEGFR-2 kinase and so preventing angiogenesis could provide a worthy approach to treating cancer.^8^

Thiadiazole is a commonly occurring 5-membered heterocyclic structure found in many biologically active natural compounds and commercially available medications.^9–11^ The core's strong aromaticity, excellent *in vivo* stability, and non-toxic nature make it highly useful in bioactive compounds.^12–14^ Through a comprehensive analysis of the literature, it has been established that many compounds based on thiadiazole have demonstrated significant inhibitory effects on the growth of tumors. Moreover, the 1,3,4-thiadiazole moiety has been introduced in many FDA-approved anticancer drugs such as filanesib.^15^ The 1,3,4-thiadiazole I was evaluated for its anticancer activity against the colon carcinoma cell line (HCT-116). It revealed promising antitumor activity with IC_50_ = 0.73 µg mL^−1^.^16^ Furthermore, compound II, which possesses the same core, revealed a remarkable ability to suppress the development of PC3 cells, with an IC_50_ value of 2.4 µM.^17^ Interestingly, the quinazoline-based 1,3,4-thiadiazole III displayed high inhibitory activity acting on dihydrofolate reductase (DHFR) with an IC_50_ value of 0.1 µM.^18^ Compound IV showed the best inhibitory effect against MCF-7 breast cancer cells among its counterparts, having an IC_50_ = 4.56 µM.^19^ Remarkably, compound V showed potent activity of IC_50_ = 1.47 µM against a panel of breast cancer cell lines.^20^ The coumarin-based 1,3,4-thiadiazole derivative VI displayed promising antitumor activity (IC_50_ = 0.90 µM) against the liver carcinoma cell line (HEPG2)^21^ (Fig. 1).

**Fig. 1:**
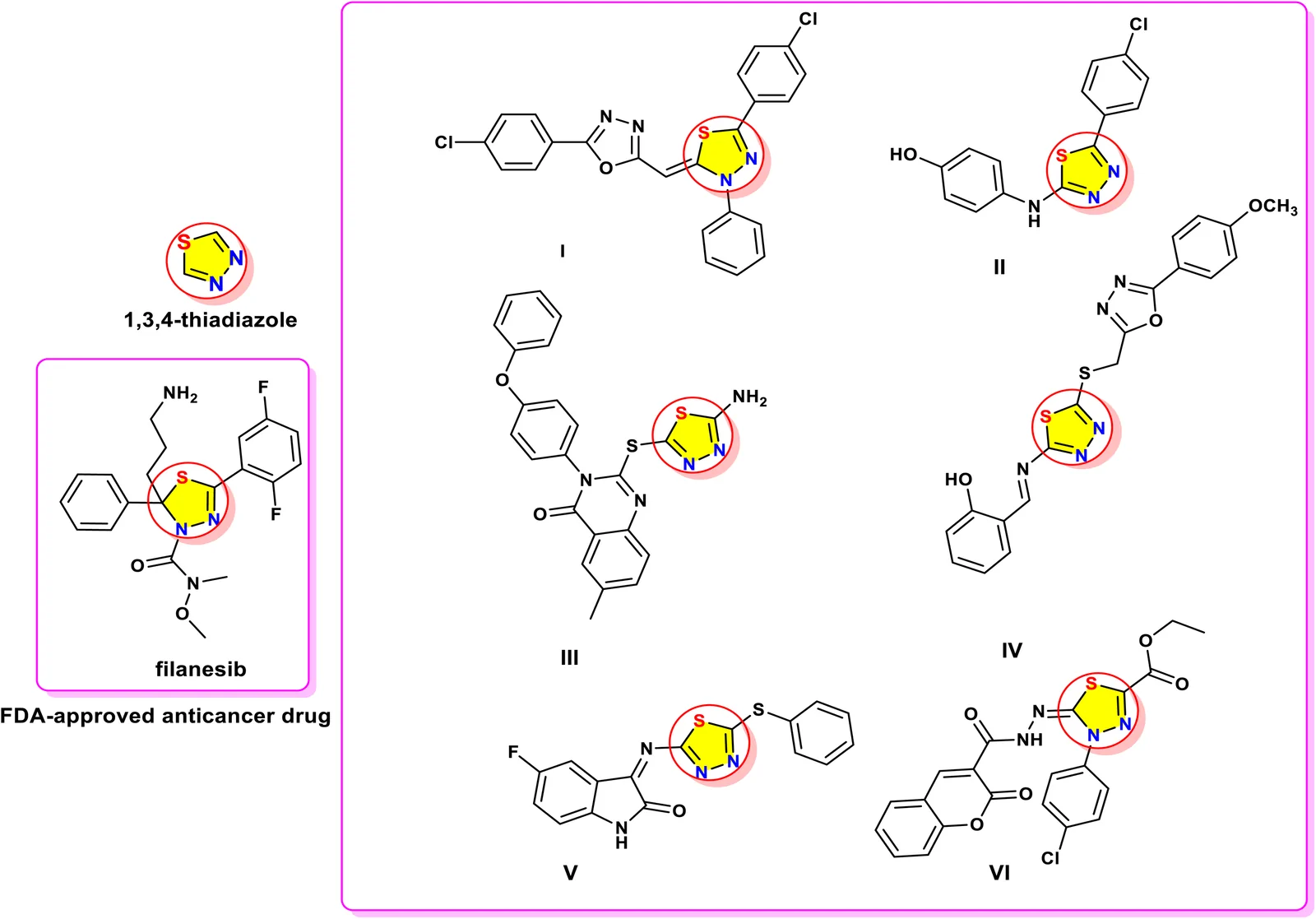
1,3,4-Thiadiazole-based anticancer reported derivatives.

Reviewing the literature on small-molecule inhibitors of VEGFR-2, different 1,3,4-thiadiazole derivatives exhibited promising VEGFR-2 inhibitory activity; among them, compound VII displayed powerful activity with an IC_50_ in the nanomolar range = 9.40 nM.^22^ The reported compounds VIII and IX, bearing a 1,3,4-thiadiazole core, have remarkably low IC_50_ values of 0.03 µM and 0.21 µM, respectively, against MCF-7 cells, and IC_50_ = 0.39 µM and 0.17 µM, respectively, against HepG2 cells. Moreover, both exhibited a VEGFR-2 inhibitory action, having IC_50_ = 0.042 and 0.041 µM, respectively, compared to sorafenib.^10,22^ Besides, compound X exhibited notably low IC_50_ values of 0.04 µM and 0.18 µM against MCF-7 and HepG2, respectively, while its VEGFR-2 inhibitory assessment revealed an appealing IC_50_ value of 103 nM (Fig. 2).^11^

**Fig. 2:**
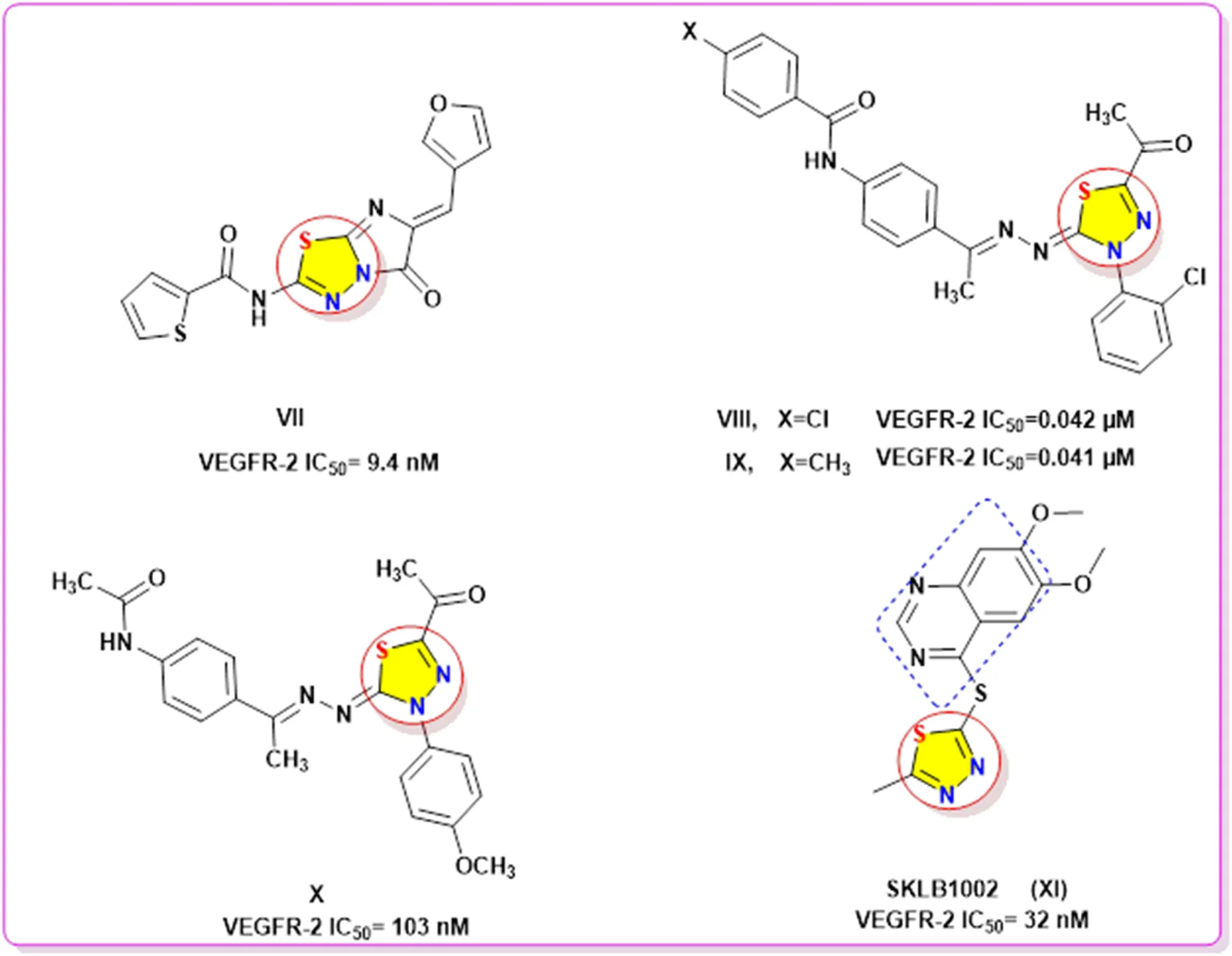
1,3,4-Thiadiazole-based VEGFR-2 reported inhibitors.

Interestingly, the quinazoline-bearing 1,3,4-thiadiazole, XI (SKLB1002), has been recognized as a potent VEGFR-2 inhibitor with minimum toxicity; it demonstrated its antiangiogenic effect by directly interacting with VEGFR2 present on the surface of endothelial cells and subsequently inhibiting the downstream signaling pathway regulated by VEGFR-2.^23–29^

EGFR is a member of the receptor tyrosine kinase family and plays a crucial role in cell signaling pathways related to cell growth, cell death, and blood vessel formation. However, increased levels of EGFR are seen as an indicator of cancer progression. Remarkably, compound XII bearing the 1,3,4-thiadiazole backbone core exhibited significant efficacy against EGFR in the nanomolar range. Also, it demonstrated apoptosis induction *via* modulating the levels of Bcl-2 and BAX.^9^ In addition, the existence of the 1,3,4-thiadiazole moiety in compound XIII was important, as it contributed to hydrogen-bonding interactions with the essential amino acids within the active site of the EGFR enzyme. Also, XIII showed potent antitumor activity against HepG2 and HCT116 cancer cell lines, with IC_50_ values of 17.2 and 14.8 µg mL^−1^, respectively. Furthermore, it has a significant inhibitory effect against EGFR, with IC_50_ = 1.22 µM.^30^ The 1,3,4-thiadiazole XIV exhibited a 32% inhibition of EGFR kinase activity and resulted in a 25% reduction in the proliferation of the MCF-7 cell line.^31^ Furthermore, XV revealed good cytotoxicity against the A549 cell line with an IC_50_ value of 1.53 µM. It showed inhibitory action on the EGFR enzyme with an IC_50_ = 0.02 µM (ref. 32) (Fig. 3).

**Fig. 3:**
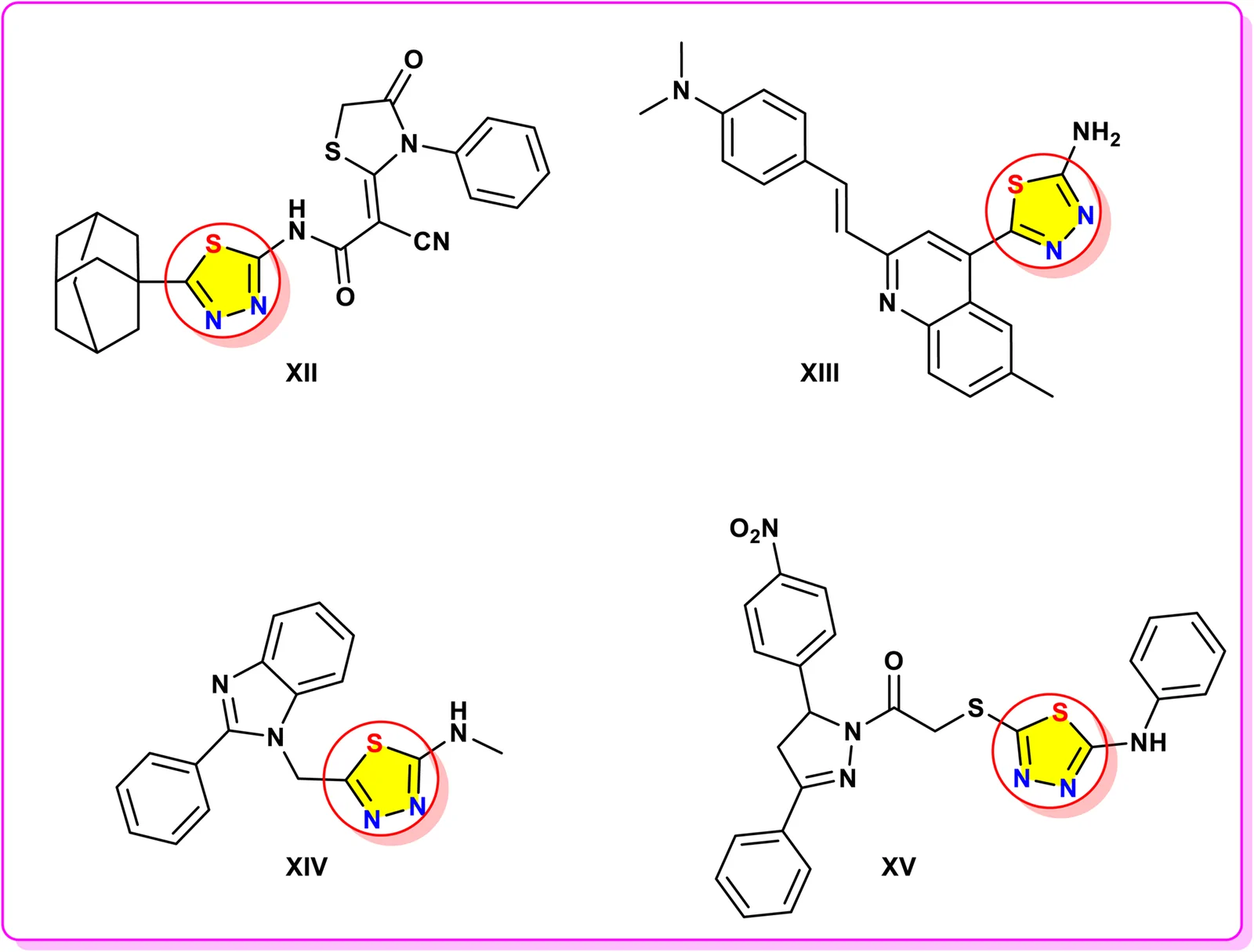
1,3,4-Thiadiazole-based EGFR-reported inhibitors.

Additionally, there has been a notable increase in the activity of both VEGFR-2 and EGFR enzymes in a diverse range of human malignancies. There are several clinically authorized anticancer medicines that effectively inhibit EGFR and VEGFR-2, including erlotinib, lapatinib, sorafenib, and sunitinib. Hence, the suppression of the VEGFR-2 and EGFR signaling pathways emerged as a compelling approach for developing novel antiproliferative medications.^33^

### 1.1. Rationale of molecular design

An analysis of the pharmacophoric characteristics shared by different VEGFR-2 inhibitors demonstrated that the majority of VEGFR-2 inhibitors exhibit four primary properties, as illustrated in Fig. 3: initially, a planar heteroaromatic ring is located within the ATP-binding domain. The second characteristic is the existence of a central aryl ring, also known as a hydrophobic spacer, that occupies the gatekeeper area of the enzyme's active site. This gatekeeper region is located in the linker region between the ATP-binding domain and the DFG domain of the enzyme. The third characteristic is the inclusion of a linker that contains a functional group that serves as a pharmacophore. This pharmacophore is responsible for binding with important residues in the DFG motif, a vital tripeptide sequence found in the active domain of the kinase. Finally, the terminal hydrophobic moiety occupies the allosteric hydrophobic pocket, resulting in the formation of many hydrophobic contacts.^34^

The molecular hybridization technique is effective, as it combines two or more distinct moieties through covalent coupling, resulting in a new molecular hybrid with potentially higher activity and efficacy compared to the original moieties.^35–37^ This is typically achieved by using a linker or spacer to connect two or more structural domains with different biological functions.^38^ Applying this strategy herein, two scaffolds, namely, quinazoline^39^ and coumarin,^6^ known for their dual mechanism of action against both enzymes,^40–42^ were introduced together with the thiadiazole core in an effort to acquire novel compounds exhibiting interesting dual activity against both enzymes.

The outcome of the drug design strategy is contingent upon the ligand used; sunitinib and compound IV were used as lead molecules for the design of our new derivatives *via* keeping the crucial pharmacophoric features. Concerning derivatives (3–8), the 1,3,4-thiadiazole core was designed to occupy the hinge region. While the methylene-amino linker signifies the pharmacophore, either the terminal coumarin moiety or diphenyl diazene groups act as the hydrophobic tail. The new hybrids (11–17) comprise a quinazoline moiety as a heterocyclic head. In addition, the (1,3,4-thiadiazol-2-yl)thio-methyl moiety is utilized as a linker in such derivatives, resembling the linker that exists in the lead compound (IV). Likewise, the methylene-amino group was retained as a pharmacophore. Finally, the terminal hydrophobic tail is represented by either a coumarin or substituted diphenyl-diazenyl group (Fig. 4).

**Fig. 4:**
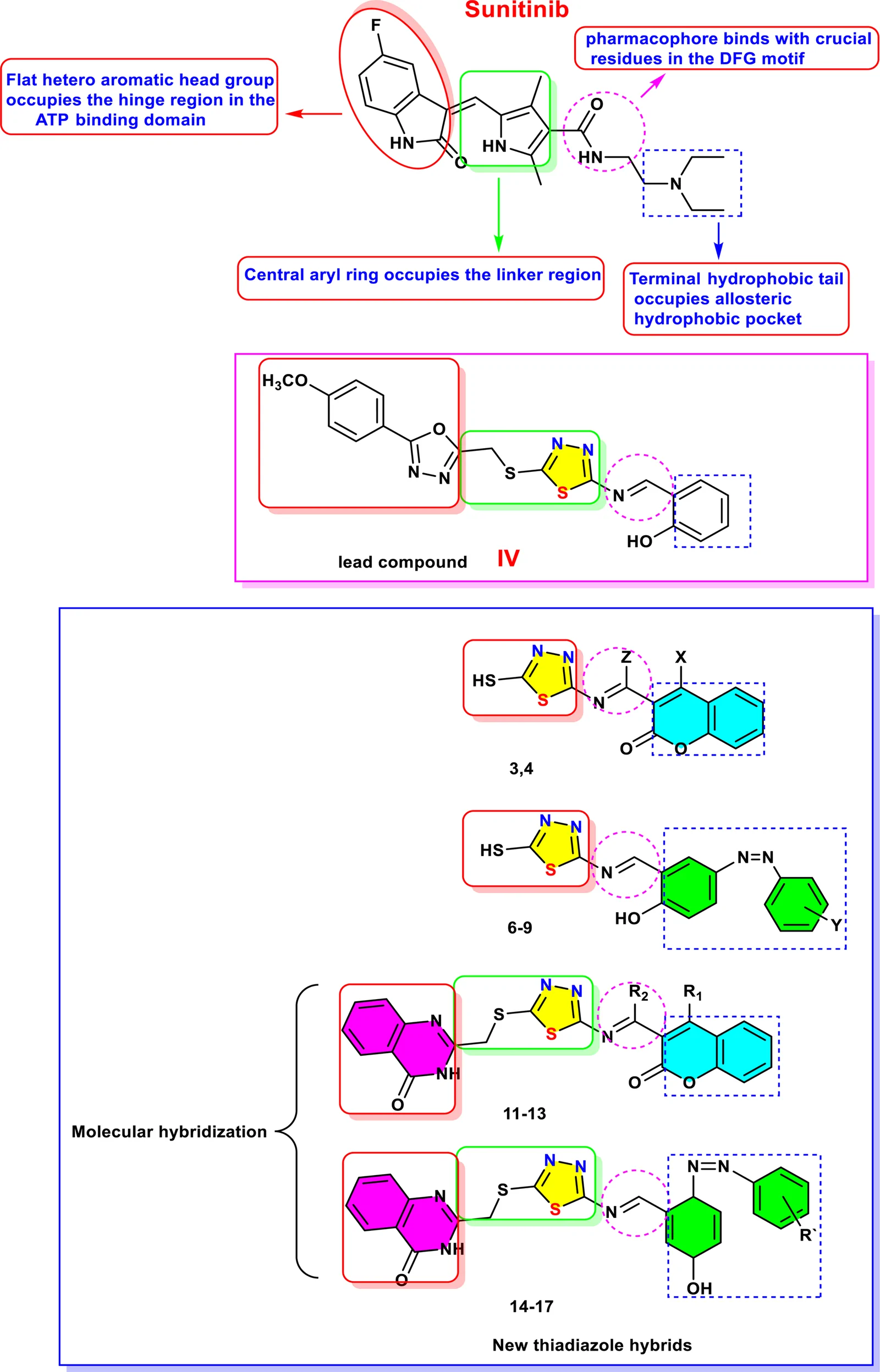
Sunitinib as a VEGFR-2 inhibitor and molecular design strategy.

The aim of our work is to synthesize new 1,3,4-thiadiazolederivatives having the essential pharmacophoric features of the clinically active VEGFR-2 inhibitors such as sunitinib. The target of our molecular design rationale comprised bioisosteric modification strategies of VEGFR-2 inhibitors (Fig. 4). First, a bioisosteric approach was adopted to replace the 5-fluoro-2-oxoindolin ring in sunitinib with a quinazoline ring. The second strategy was replacing the central pyrrole ring with a 1,3,4-thiadiazole moiety, aiming to increase the binding affinity to the VEGFR-2 active site. The third strategy was using a methylene amino linker in all derivatives resembling the linker present in the lead compound (IV). Also, the hydrophobic tail of the reported ligand sunitinib was replaced by a coumarin or substituted diphenyl-diazenyl group. The designed targets, the 1,3,4-thiadiazole derivatives, were synthesized and evaluated for antitumor activities against three human cancerous cell lines, namely breast cancer (MCF-7), hepatocellular carcinoma (HepG-2), and human colorectal carcinoma (HCT116). Additionally, compounds eliciting promising anticancer activity were investigated as dual inhibitors of EGFR and VEGFR-2 activity to shed light on their possible cellular mechanism. Meanwhile, a molecular docking study for the synthesized compounds on the ATP-binding site of VEGFR-2 was carried out to reveal the inhibitory binding mode.

## 2. Results and discussion

### 2.1. Organic synthesis

In accordance with Scheme 1, 5-amino-1,3,4-thiadiazole-2-thiol 1 can be used as a starting material to squarely synthesize new Schiff bases. Following the analogous method in previous works,^43–48^ compound 1 was synthesized. By using a catalyst of an inorganic base, like anhydrous sodium carbonate (Na_2_CO_3_), and absolute ethanol as the reaction solvent, hydrazine carbothioamide (CH_5_N_3_S) was refluxed with methanedithione (CS_2_). Subsequently, derivative 1 was directly condensed with various aldehydes 2a–g in absolute ethanol, yielding intermediates 3–9*via* a condensation reaction in an acidic medium (a few drops of concentrated sulfuric acid).^49,50^

**Scheme 1:**
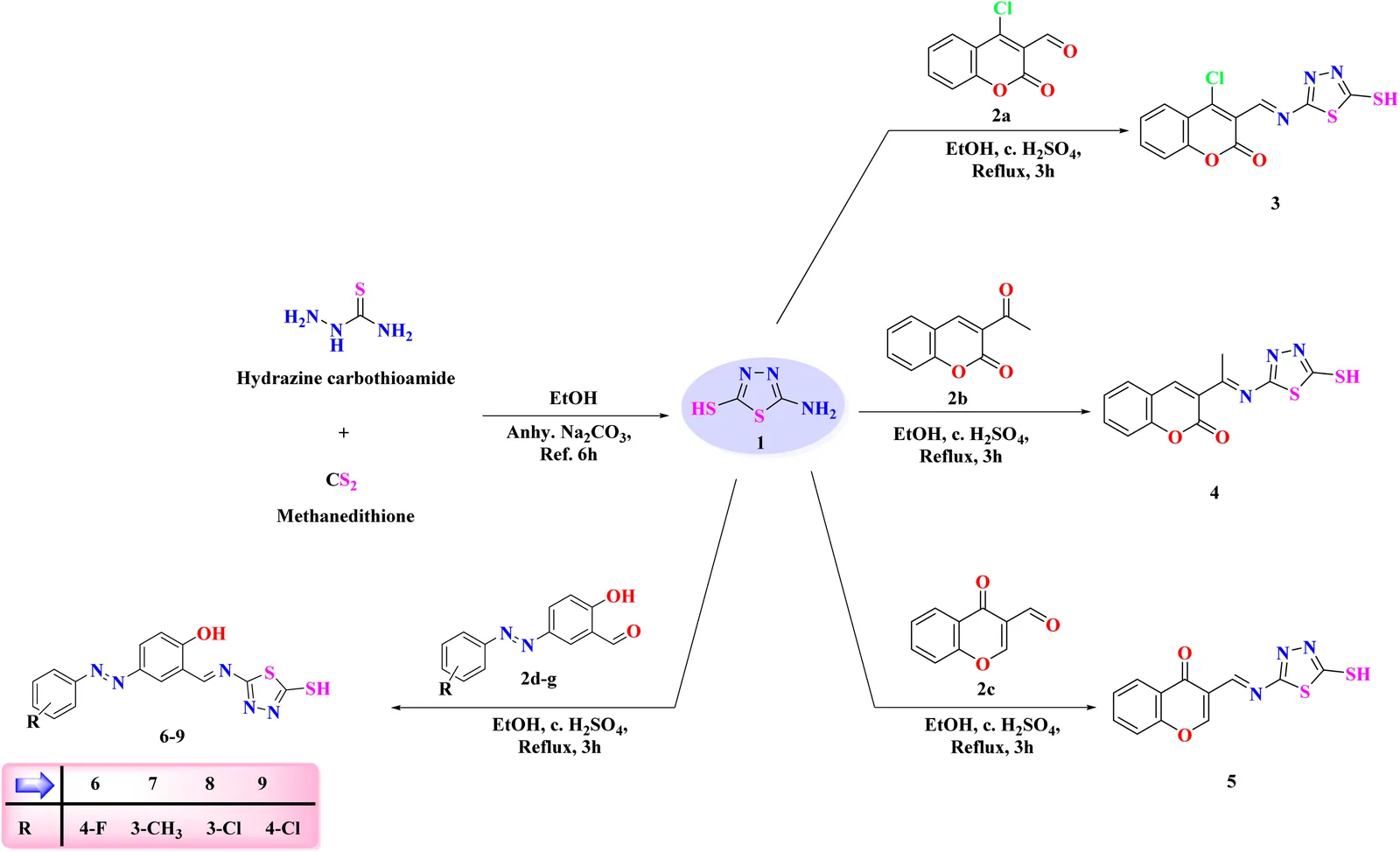
The preparation of Schiff base derivatives linked to mercapto-1,3,4-thiadiazole.

The structural elucidation of the newly synthesized thiadiazole derivatives 3–9 was achieved through spectral data analysis. This is demonstrated in the SI, where FTIR-KBr spectroscopy reveals the disappearance of amino bands between 3395 cm^−1^ and 3275 cm^−1^, replaced by new bands between 3414 cm^−1^ and 3353 cm^−1^ in this region. The presence of absorption bands was observed between 1713 cm^−1^ and 1607 cm^−1^, corresponding to carbonyl and imine groups, respectively. In addition, derivatives 6–9 exhibited additional absorption bands at 1449–1482 cm^−1^, indicative of the subsistence of an azo group. On the other hand, the assigned structural framework of derivatives 3–9 was validated through measurements of their proton and carbon nuclear magnetic resonance spectra. The ^1^H NMR spectra exhibited a singlet signal at *δ*_H_ = 14.97–13.16 ppm belonging to one thiol (SH) proton; additionally, the distinctive singlet signal in compounds 6–9 corresponds to the hydroxyl proton, observed within the range of *δ*_H_ = 10.40–10.35 ppm, and the azomethine proton (–CH=N–) manifested as a singlet peak in the *δ*_H_ = 9.10–8.00 ppm range. Furthermore, the protons of both coumarin-4H and chromene-2H were observed as singlets at *δ*_H_ = 8.64 ppm and *δ*_H_ = 10.12 ppm, respectively. Whereas the protons of salicylaldehyde and *m*-,*p*-substituted aryl groups appeared at the expected chemical shifts within the range of *δ*_H_ = 9.20–9.10 ppm and *δ*_H_ = 8.20–6.87 ppm as a singlet and multiple signals, respectively. Details regarding the ^13^C NMR and mass spectra of all intermediates are provided in the SI Section.

Subsequently, in Scheme 2, reacting unsubstituted *o*-anthranilic acid with chloroacetonitrile in methanol and sodium methylate (CH_3_O^−^Na^+^) as a base at 0 °C, the intermediate 2-(chloromethyl) quinazoline-4(3*H*)-one 10 was obtained.^51^ In an alcoholic potassium hydroxide solution, compound 10 underwent a nucleophilic substitution reaction with the intermediates 3–9, producing the expected compounds 11–17, as sketched in Scheme 2.

**Scheme 2:**
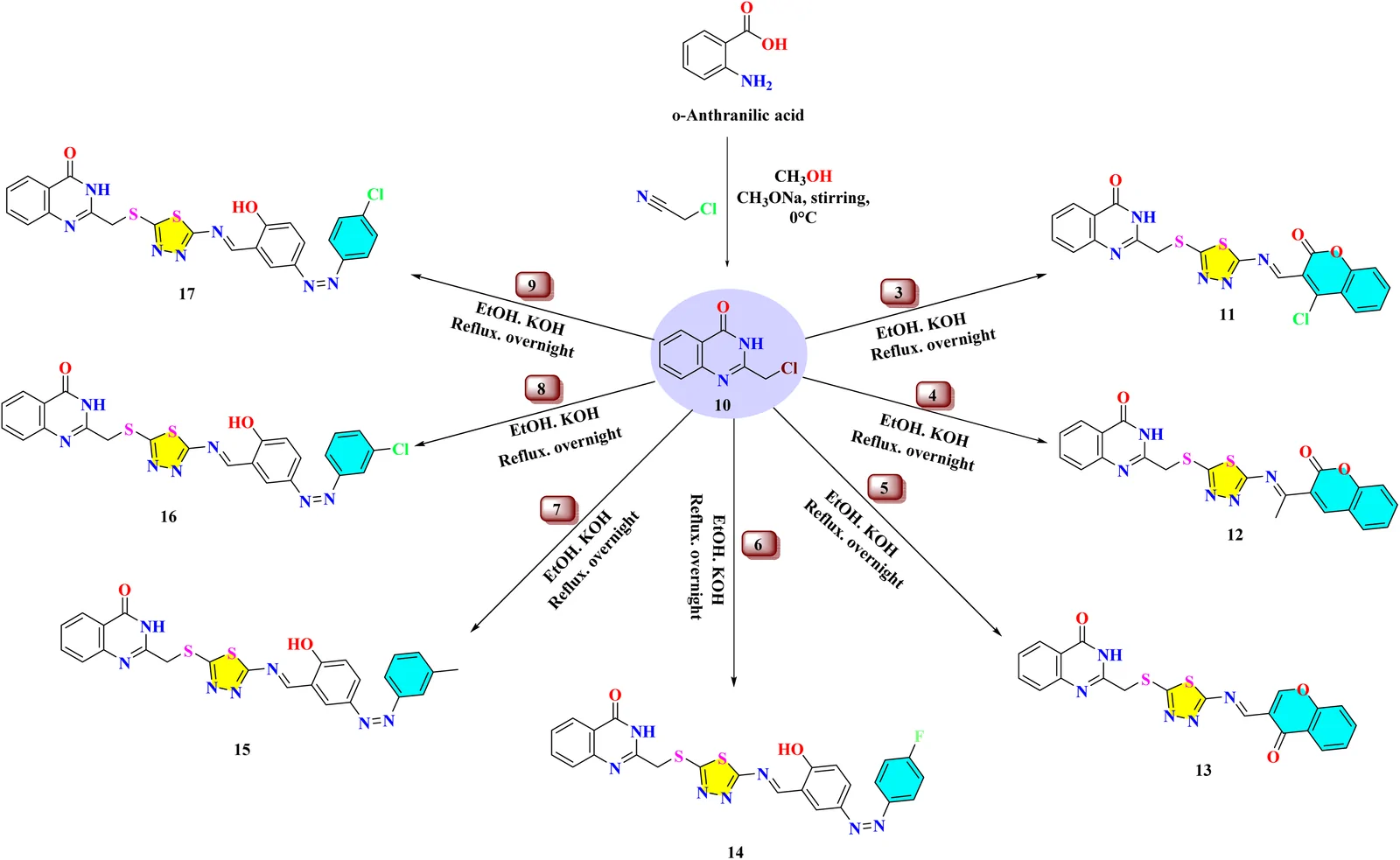
The preparation of novel mercapto thiadiazole–quinazoline derivatives 11–17.

FTIR analysis revealed unique peaks, in addition to pre-existing signals at the specified ranges, suggesting the presence of both imino and carbonyl groups within the carbon framework of the synthesized compounds. The ^1^H NMR of these derivatives displays distinct signals within *δ*_H_ = 4.25–3.65 ppm, indicating the presence of protons of the methylene group between quinazolinone and the sulfur atom attached to the thiadiazole ring. The results suggest a successful alkylation reaction between derivative 10 and the thiadiazole derivatives 3–9. The methylene group is also observed in up-field ^13^C NMR at *δ*_C_ = 30.42 ppm to *δ*_C_ = 37.71 ppm. In contrast, the remaining groups are present at their distinct positions within both the ^1^H and ^13^C NMR data. Moreover, the structure of the target compounds was also established based on mass spectrometry, as shown in the SI Section.

### 2.2. Biological studies

#### 2.2.1. Cytotoxicity activity

The cytotoxic effect of twelve newly synthesized targeted compounds were evaluated *in vitro* against cell lines that represent breast (MCF-7), hepatic (HepG-2), and colorectal (HCT-116) carcinomas. The cytotoxic effect of these compounds was assessed using the MTT assay, with doxorubicin, a well-known chemotherapeutic drug, serving as a reference standard. The IC_50_ values of the compounds under test and doxorubicin were indicated in Table 1. In general, compounds 7, 8, 15, and 16 demonstrated noteworthy anticancer properties; compounds 15 and 16, in particular, were the most effective derivatives against the three cell lines. Based on the acquired data, it was found that the remaining compounds under test exhibited a moderate cytotoxic impact, with an IC_50_ range of 3.47–20.11 µM.

**Table 1:** Cytotoxicity results of new compounds along with doxorubicin assessed in three cancer cell lines^a^

Compounds	IC_50_ (µM)		
	MCF-7	HepG-2	HCT-116
3	15.72 ± 0.12	12.85 ± 0.10	11.34 ± 0.10
4	17.25 ± 0.15	16.41 ± 0.13	13.25 ± 0.11
5	20.11 ± 0.18	15.75 ± 0.14	14.26 ± 0.10
6	6.84 ± 0.04	5.22 ± 0.03	3.47 ± 0.02
7	4.27 ± 0.03	4.10 ± 0.02	2.54 ± 0.05
8	4.11 ± 0.03	3.08 ± 0.024	1.58 ± 0.015
9	8.35 ± 0.06	6.21 ± 0.04	5.62 ± 0.03
11	11.94 ± 0.08	9.36 ± 0.07	7.52 ± 0.05
12	14.31 ± 0.11	14.28 ± 0.10	10.36 ± 0.09
13	10.41 ± 0.08	8.55 ± 0.06	7.35 ± 0.05
14	9.65 ± 0.08	10.18 ± 0.1	8.24 ± 0.06
15	3.95 ± 0.01	2.87 ± 0.05	1.75 ± 0.01
16	3.14 ± 0.02	2.74 ± 0.01	2.05 ± 0.01
17	9.58 ± 0.07	8.59 ± 0.06	7.16 ± 0.06
Doxorubicin	**4.94 ± 0.81**	**7.33 ± 0.57**	**3.51 ± 0.21**

a Data are expressed as the mean ± SD of three independent experiments.

#### 2.2.2. Structure–activity relationship (SAR)

Regarding compounds 3–5, which have moderate activity between 11.34 and 20.11 µM, the thiadiazole derivatives with a coumarin scaffold (3 and 4) demonstrated a moderate degree of activity across the three assessed cell lines. Similarly, compound 5 exhibited moderate activity in the presence of a chromone moiety. The incorporation of a quinazoline ring in compounds 11–13, resulting in a hybrid of three moieties (thiadiazole, coumarin, and quinazoline), significantly enhanced the activity, yielding an IC_50_ range of 7.52–14.31 µM.

The synthesis of a hybrid molecule comprising thiadiazole and diphenyl diazene led to a notable increase in activity, as evidenced by the thiadiazole derivatives, compounds 6–9. The assessed activity demonstrated an IC_50_ range of 1.58 to 8.35 µM. Furthermore, the incorporation of hydrophobic groups, specifically CH_3_ or Cl, at the meta position of the terminal phenyl ring of the diphenyl diazene moiety, as demonstrated in compounds 7 and 8, displayed superior efficacy on the examined cell lines, surpassing that of doxorubicin, in contrast to their counterparts 6 and 9, which possess substituents at the para position.

The measured IC_50_ results of the synthesized hybrids indicate that both the diphenyldiazene scaffold and the quinazoline ring significantly affect activity when interacting with thiadiazole. Motivated by these results, thiadiazole hybrids 14–17 were synthesized by maintaining the primary backbone (thiadiazole) and including two relevant moieties to investigate their impact on activity. Compounds 15 and 16, featuring hydrophobic substituents at the meta position of the terminal phenyl ring of the diphenyl diazene moiety, exhibited the highest activity among the evaluated derivatives, surpassing the standard drug's efficacy. These data corroborated our prior hypothesis and motivated us to pursue further assessment.

#### 2.2.3. *In vitro* VEGFR-2 inhibitory assay

This study aims to provide a mechanistic understanding of the anticancer properties of our most potent compounds, specifically compounds 7, 8, 15, and 16. These compounds have demonstrated the most significant cytotoxic effects against MCF-7, HepG-2, and HCT-116 carcinoma cell lines. The VEGFR-2 inhibitory activity of the entities was assessed by employing the conventional pharmaceutical agent sorafenib. The acquired results, expressed as IC_50_ (µM), are displayed in Table 2. Compounds 7 and 15 had the highest level of activity, as seen by their respective IC_50_ values of 0.17 and 0.16 µM. In comparison, sorafenib demonstrated an IC_50_ value of 0.10 µM. In contrast, compounds 8 and 16 exhibited a 9-fold decline in activity compared to the reference medication. The obtained results indicated the importance of the CH_3_ group as a substituent on the diphenyl diazene scaffold, showing nearly equipotent activity to the reference and preceding the other two derivatives bearing the Cl group.

**Table 2:** VEGFR-2 inhibitory activity of compounds 7, 8, 15, and 16^a^

Compound	IC_50_ (µM)
7	0.17 ± 0.005
8	0.94 ± 0.050
15	0.16 ± 0.002
16	0.91 ± 0.070
Sorafenib	0.10 ± 0.020

a Data are expressed as the mean ± SD of three independent experiments.

#### 2.2.4. *In vitro EGFR* inhibitory assay

The purpose of conducting the EGFR inhibitory test was to investigate the potential mechanism underlying the dual effect induced by the newly synthesized hits. The produced compounds that had the greatest efficacy against VEGFR-2 and resulted in significant inhibition of cancer cell proliferation were evaluated for their ability to inhibit the EGFR tyrosine kinase enzyme. Upon analysis of the recently synthesized compounds (Table 3), it was shown that the derivatives based on 1,3,4-thiadiazole and diphenyl diazene (7 and 15) exhibited a significant degree of inhibitory activity against EGFR. This finding is consistent with the results obtained from prior experiments conducted on cell lines.

**Table 3:** EGFR inhibitory activity of compounds 7 and 15^a^

Compound	IC_50_ (µM)
7	0.42 ± 0.5
15	0.23 ± 0.02
Erlotinib	0.22 ± 0.01

a Data are expressed as the mean ± SD of three independent experiments.

#### 2.2.5. Cytotoxic activity on normal cells

In order to ensure selectivity and safety, the most promising and highly dual-active novel compounds were subjected to additional evaluation through the assessment of their cytotoxicity against normal noncancerous cells (WI-38 cells), as presented in Table 4. The findings of the study indicate that compounds 7 and 15 had IC_50_ values of 59.58 and 50.15 µM, respectively. These results suggest that these compounds possess selective cytotoxic properties against cancer cell lines while demonstrating a comparatively lower cytotoxic effect on normal fibroblasts (WI-38).

**Table 4:** Cytotoxicity of compounds 7 and 15 against the normal cell line WI-38^a^

Compound	IC_50_ (µM)		Selectivity index^b^	
	WI-38	MCF-7	HepG-2	HCT-116
7	59.58 ± 1.7	13.95	14.50	23.45
15	50.15 ± 1.3	12.69	17.47	28.65

a Data are expressed as the mean ± SD of three independent experiments.

b Selectivity index = IC_50_ on WI-38/IC_50_ on cancer cells.

#### 2.2.6. Molecular docking study

##### 2.2.6.1. Docking inside the VEGFR-2 active site

This study aimed to evaluate the binding manner of the most active compounds, 7 and 15, against the proposed target VEGFR-2. To confirm the validity of our design, docking experiments were attained *via* Autodocking Vina, a software program that specially requires both receptors and ligands to be in pdbqt format.^52^ The 3D coordinates of VEGFR-2 in its X-ray crystal structure (PDB ID: 4ASD) were obtained from the Protein Data Bank website.^53^ The structure has a resolution of 2.03 Å and consists of 353 amino acids. The reference molecule employed was the co-crystallized ligand sorafenib. Discovery Studio 4.5 visualizer was utilized to visually depict the results. The predicted binding affinity (docking score) of compound 15 was −12.02 kcal mol^−1^, which suggests a potentially stronger interaction compared to the theoretical score of sorafenib (−11.41 kcal mol^−1^). In order to verify the docking techniques, the co-crystallized ligand (sorafenib) was re-docked with VEGFR-2; it displayed a docking score (−11.41 kcal mol^−1^). The obtained root mean square deviation (RMSD) value was 0.172, indicating the successful validation of the implemented docking methodology.

The co-crystallized ligand interacted with the active site of VEGFR-2 by the following interactions: the *N*-methylpicolinamide motif formed 2H-bonds with Cys919 in the hinge region, while the ureido group interacted *via* three hydrogen bonds: Asp1046 formed 1H-bond with the carbonyl group, whereas Glu885 interacted *via* 2H-bonds with the same moiety in the DGF domain. In addition, different hydrophobic interactions were observed between the central phenyl ring occupying the linker region and Val848, Leu899, Val916, and Phe1047 residues. Also, it formed a pi–cation interaction with Lys868. The terminal phenyl ring interacted with Leu889 *via* a pi–alkyl interaction, occupying the allosteric binding region (Table 5 and Fig. 5).

**Table 5:** Docking results of the co-crystallized ligand (sorafenib) and the most active compounds 7 and 15, inside the active site of the VEGFR-2 enzyme

Comp.	Docking score (kcal mol^−1^)	Interacting residues	
7	−10.23	Ala866	Lys868
		Glu885	Leu889
		Val899	Val916
		Cys919	Leu1035
			Cys1045
15	−12.02	Leu840	Val848
		Ala866	Phe918
		Cys919	Asn923
		Arg1051	Leu1035
		Phe1047	Lys1055
Sorafenib	−11.41	Val848	Ala866
		Lys868	Glu885
		Leu889	Val899
		Val916	Glu917
		Phe918	Cys919
		Lys920	Leu1035
		Cys1045	Asp1046
		Phe1047	

**Fig. 5:**
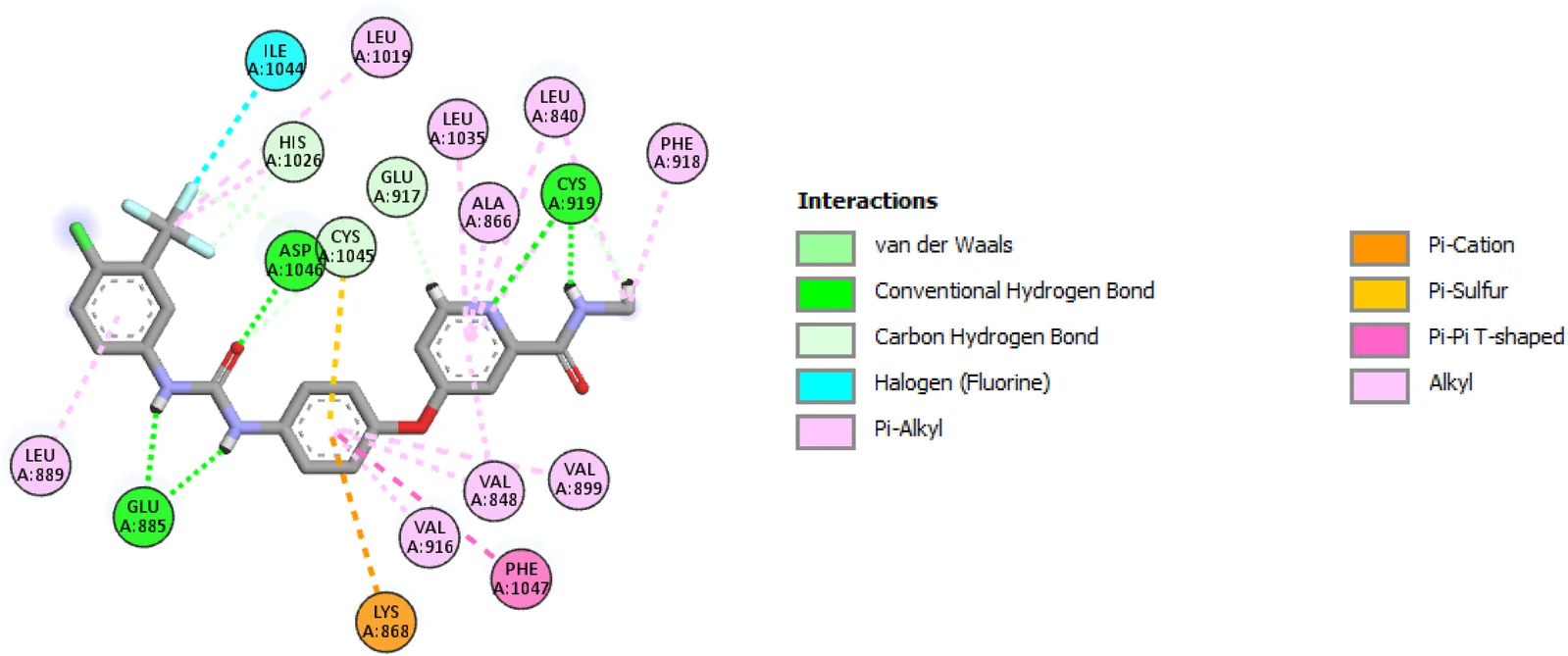
The two-dimensional suggested binding mode of sorafenib inside the VEGFR-2 active site.

Furthermore, studying the binding site of VEGFR-2 docked with compound 7 indicated that our new hit binds to essential amino acids in the binding site, resembling sorafenib, with a binding score of −10.23 kcal mol^−1^. In the hinge region, two hydrogen bonds were discovered between Cys919 (the essential amino acid in the ATP-binding site) and the thiadiazole moiety. The same ring successfully engaged in two hydrophobic contacts with Ala866 and Leu1035. Additionally, the diazenyl group formed an additional two hydrogen bonds with the conserved amino acids in the DFG domain (Glu885 and Asp1046). Moreover, the central phenyl ring facilitated enhanced contacting the form of a pi–cation interaction with Lys868 in the gatekeeper region, in addition to pi–sigma and pi–alkyl contacts with Val916 and Val899 residues, respectively, ensuring optimal fitting. Whereas the terminal phenyl ring occupied the allosteric hydrophobic region *via* a pi–sigma interaction with Leu889. The aforementioned data strongly demonstrate compound 7's potential to inhibit VEGFR-2 (Fig. 6 and 7).

**Fig. 6:**
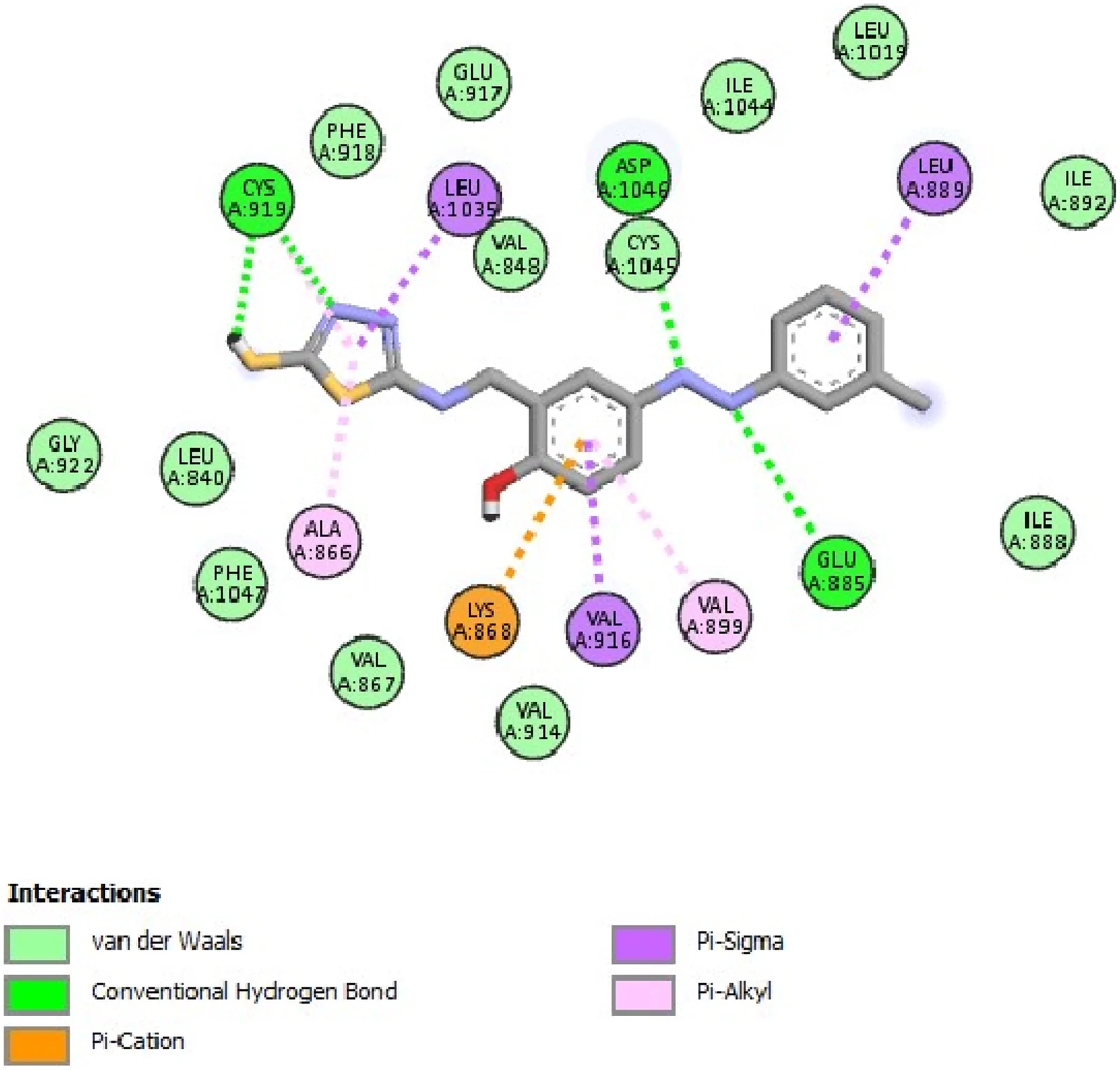
The two-dimensional suggested binding mode of compound 7 inside the VEGFR-2 active site.

**Fig. 7:**
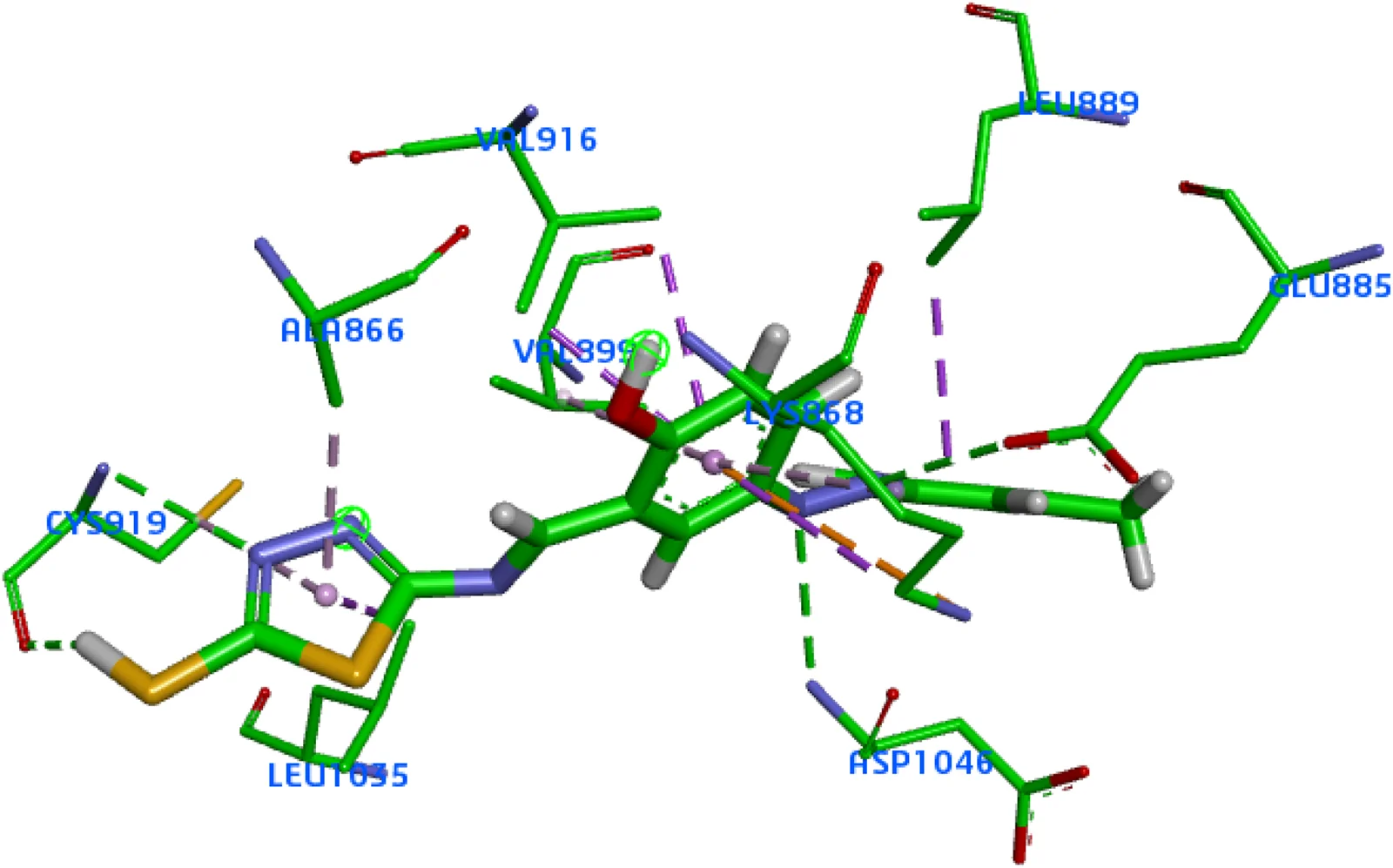
The three-dimensional suggested binding mode of compound 7 inside the VEGFR-2 active site.

The proposed binding mode of compound 15 revealed an affinity value of-12.02 kcal mol^−1^ and two conventional H-bonds. The quinazoline moiety was stabilized and oriented inside the hinge region by the formation of one H-bond with Cys919. In addition, the quinazoline scaffold was able to perform different hydrophobic interactions (pi–pi stack, pi–alkyl, alkyl, and pi–sigma) with Phe918, Ala866, Leu1035, and Val848 amino acids, respectively. Moreover, the sulphur atom of the thiadiazole ring contributed to binding with the active site by a hydrogen bond with Asn923 and a pi–sulphur interaction with Phe1047. Also, a pi-donor hydrogen bond exists between the methylene-amino moiety, representing the pharmacophore, and Leu840. Finally, the terminal diphenyldiazenyl scaffold was engaged with Arg1053 and Lys1055 by the two phenyl rings *via* pi–alkyl and pi-donor hydrogen bonds, respectively (Fig. 8 and 9).

**Fig. 8:**
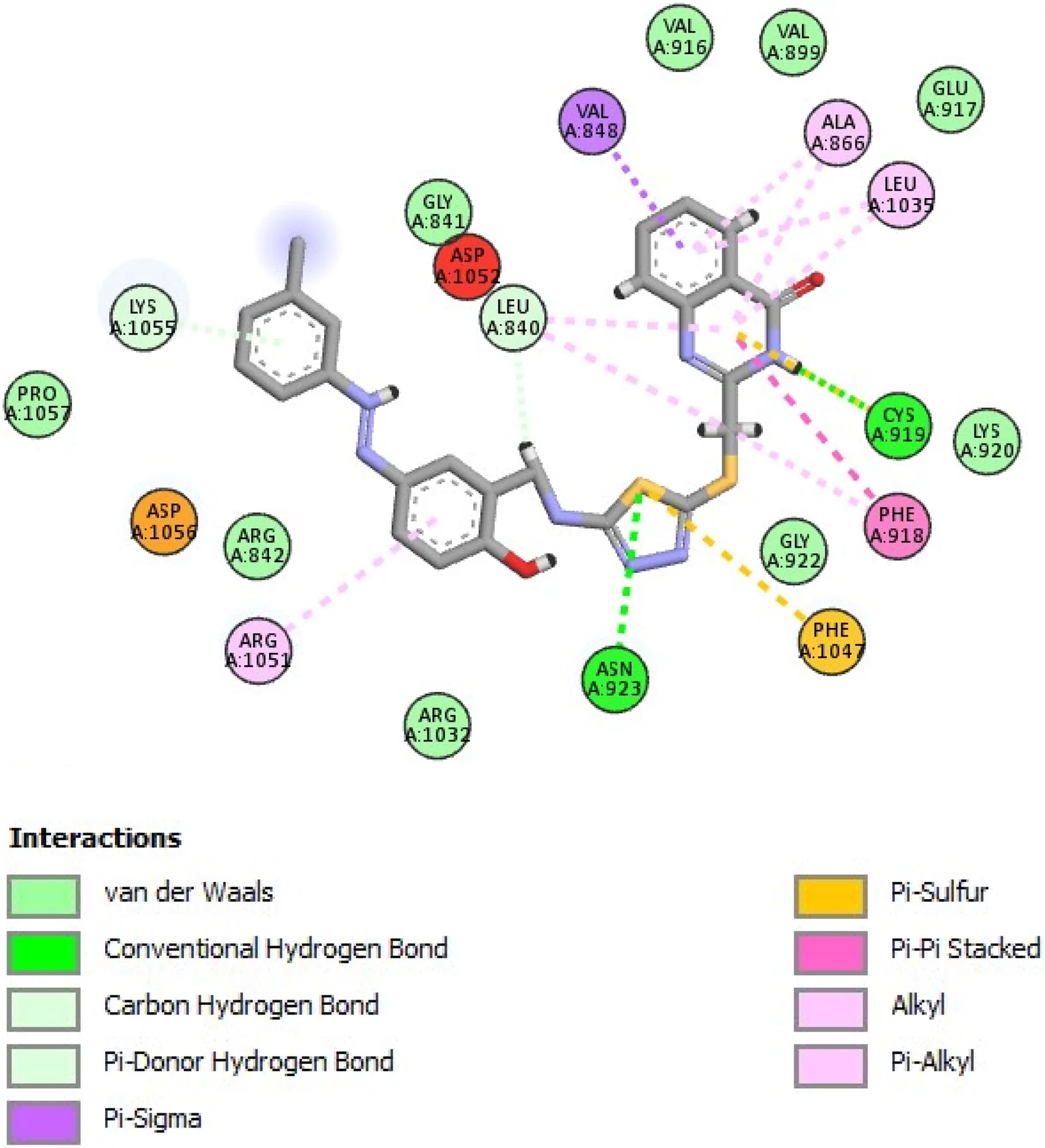
The two-dimensional suggested binding mode of compound 15 inside the VEGFR-2 active site.

**Fig. 9:**
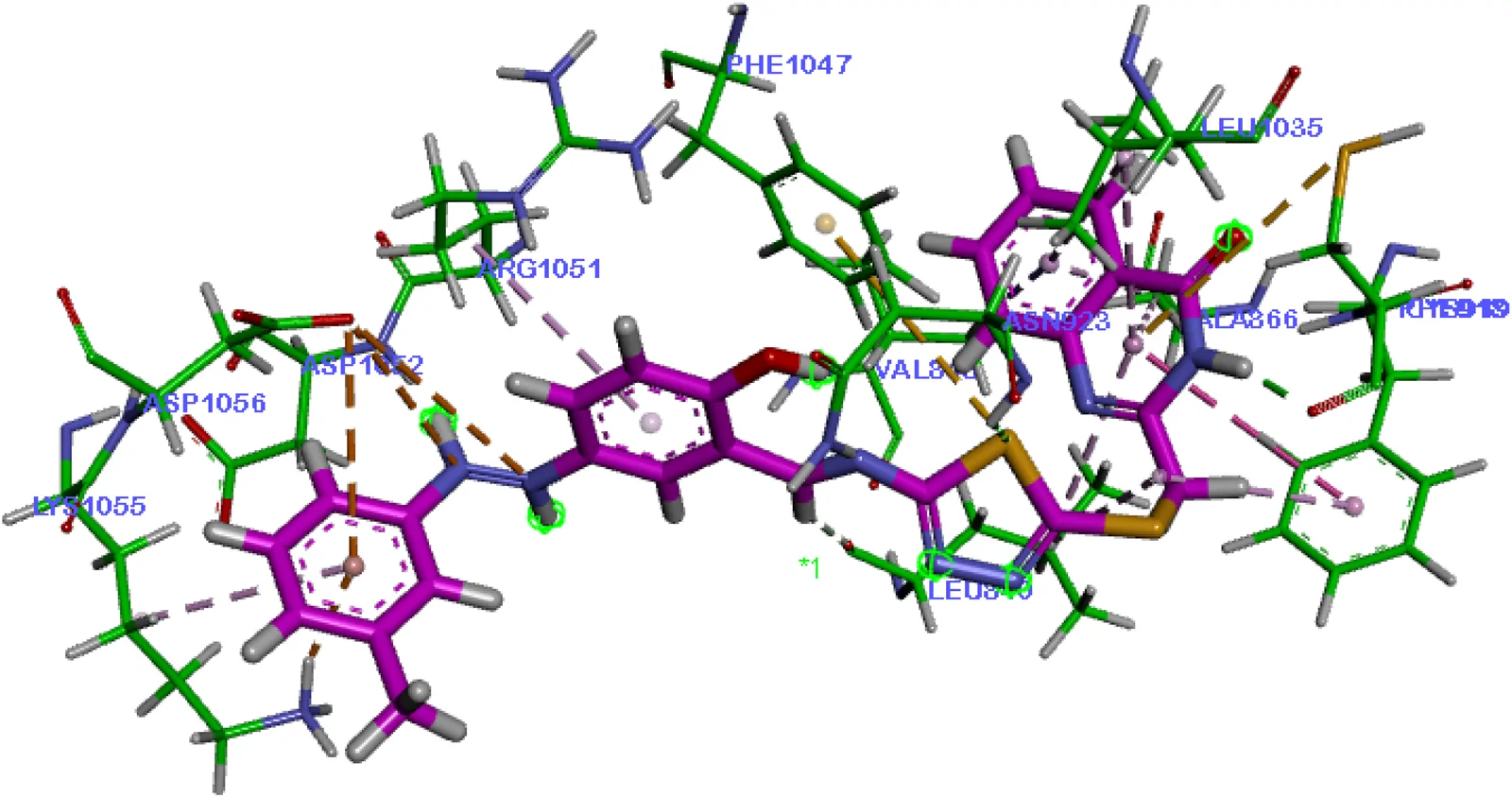
The three-dimensional suggested binding mode of compound 15 inside the VEGFR-2 active site.

##### 2.2.6.2. Docking inside the EGFR active site

Docking of both compounds 7 and 15 in the active site of EGFR was performed on EGFR in complex with erlotinib, downloaded from the Protein Data Bank with PDB code (1M17).^54^ Redocking of the co-crystallized ligand was first carried out for validation. It revealed a root-mean-square deviation (RMSD) value = 1.59 Å and a docking score = −10.16 kcal mol^−1^. Erlotinib formed multiple interactions within the ATP-binding pocket *via* hydrogen bonding with Thr830. Also, carbon–hydrogen bonds were recorded with Gln767, Glu738, and Asp831 amino acids. Besides, the quinazoline moiety contributed *via* pi–alkyl interactions with Ala719, Met769, and Leu820. Finally, Leu768 and Lys704 were bonded by two alkyl interactions with the terminal ethynyl group (Fig. 10).

**Fig. 10:**
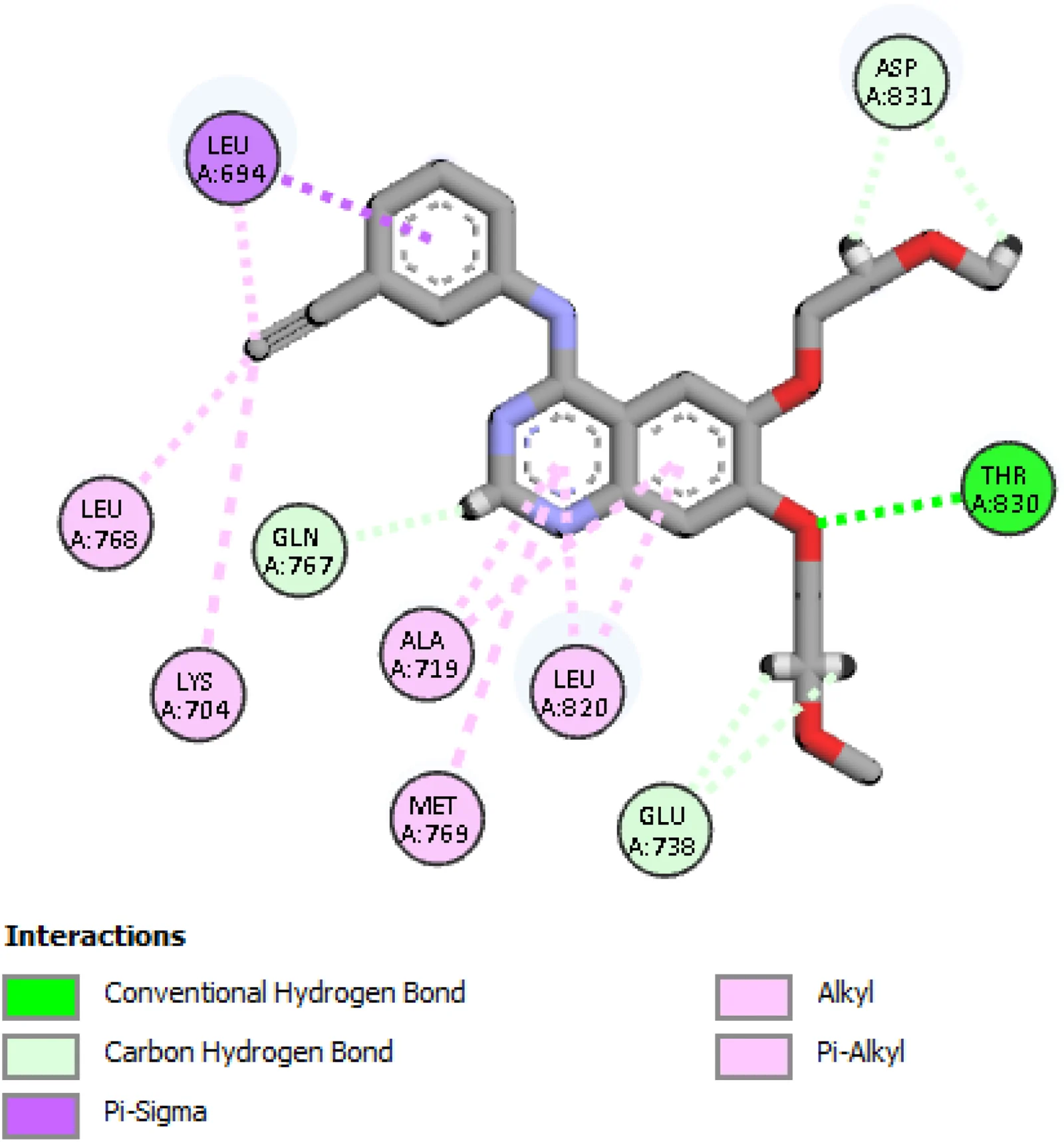
The two-dimensional suggested binding mode of sorafenib inside the EGFR active site.

Interestingly, the tested compounds 7 and 15 demonstrated the ability to interact with key residues within the ATP-binding site, which may explain their promising *in vitro* cytotoxic activity. This finding was further supported by their favorable docking scores and binding orientations (Fig. 11–14 and Table 6).

**Table 6:** Docking results of the co-crystallized ligand (erlotinib) and the most active compounds 7 and 15 inside the active site of the EGFR enzyme

Comp.	Docking score (kcal mol^−1^)	Interacting residues
7	−9.64	Leu694, Ala719, Lys721
		Met742, Thr766, Glu738
		Leu820, Asp831, Phe832
15	−10.64	Leu694, Lys704, Ala719, Lys721
		Met742, Leu753, Leu764, Leu768
		Met769, Pro770, Gly772, Cys773, Leu820
		
Erlotinib	−10.16	Leu694, Lys704, Ala719, Glu738
		Gln767, Leu768, Met769, Leu820
		Thr830, Asp831

Focusing on compound 7, displaying good EGFR inhibitory activity compared with erlotinib, it exhibited a docking score of −9.64 kcal mol^−1^, and it participated in binding with two hydrogen bonds; the mercapto group formed a hydrogen bond with Glu738, and the other H-bond was between the hydroxyl group and Thr766 residue. Moreover, pi–alkyl interactions were observed between the aromatic rings of compound 7 and Leu694, Ala719, Lys721, Met742, and Leu820 (Fig. 11 and 12).

**Fig. 11:**
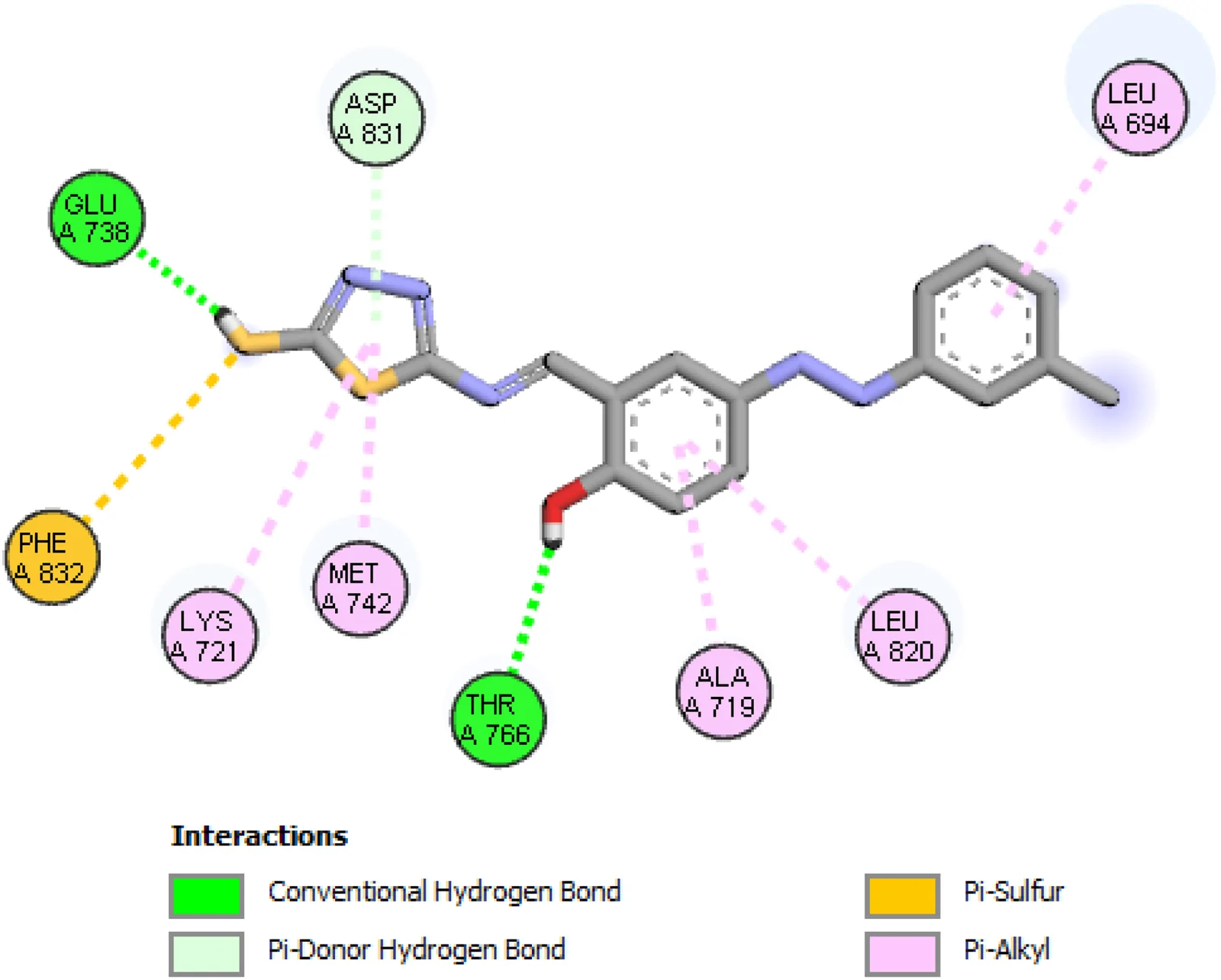
The two-dimensional suggested binding mode of compound 7 inside the EGFR active site.

**Fig. 12:**
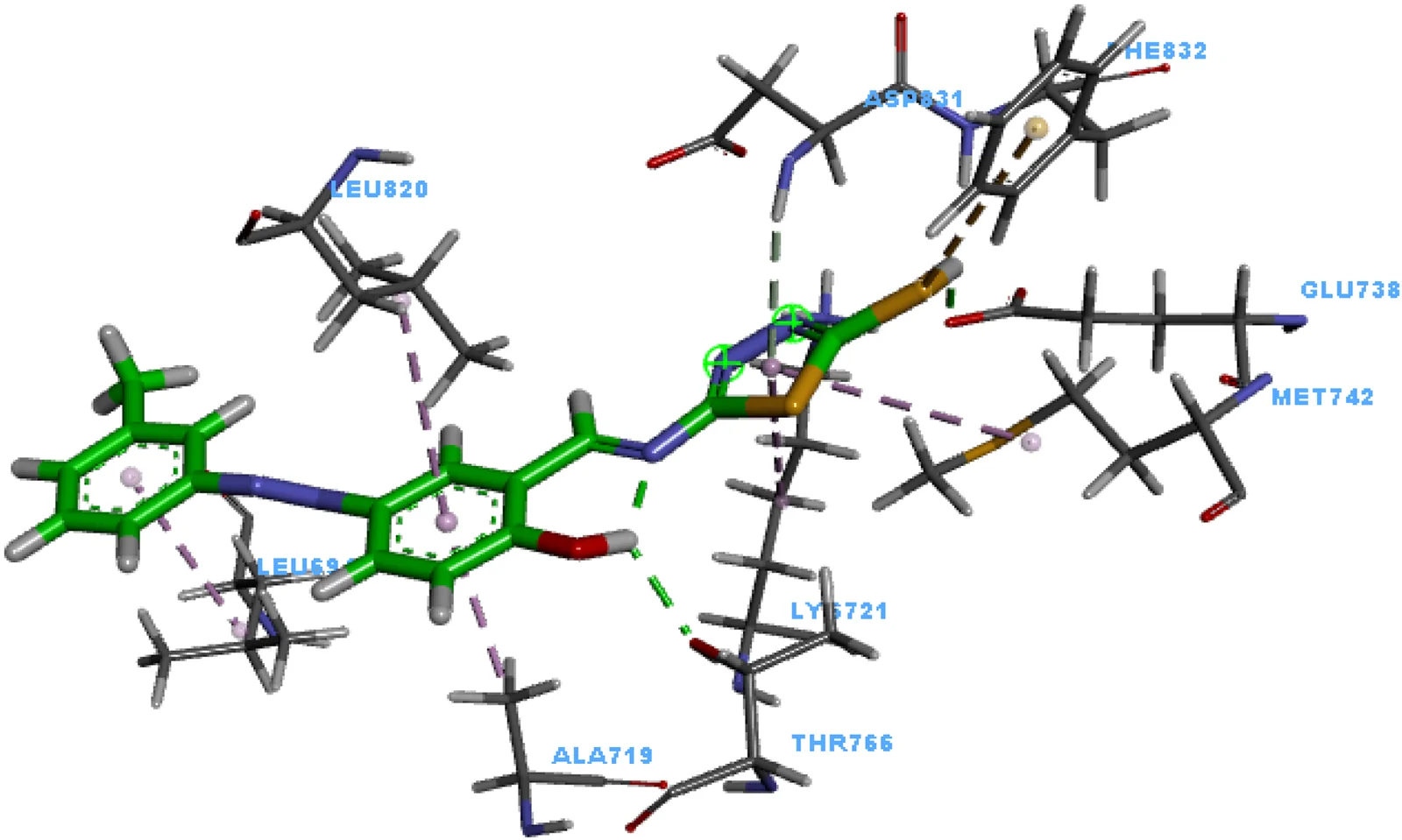
The three-dimensional suggested binding mode of compound 7 inside the EGFR active site.

Regarding compound 15, it recorded a docking score = −10.64 kcal mol^−1^, and it formed three hydrogen bonds; the hydroxyl group formed two hydrogen bonds with the most essential amino acid in the active site, Met769. The third one is between one nitrogen of the thiadiazole ring and Cys773. Moreover, several pi–alkyl interactions were observed between the aromatic rings of the structure and the essential residues in the binding pocket: Leu694, Lys704, Ala719, Lys721, Met742, Leu753, Leu764, Leu820 (Fig. 13 and 14).

**Fig. 13:**
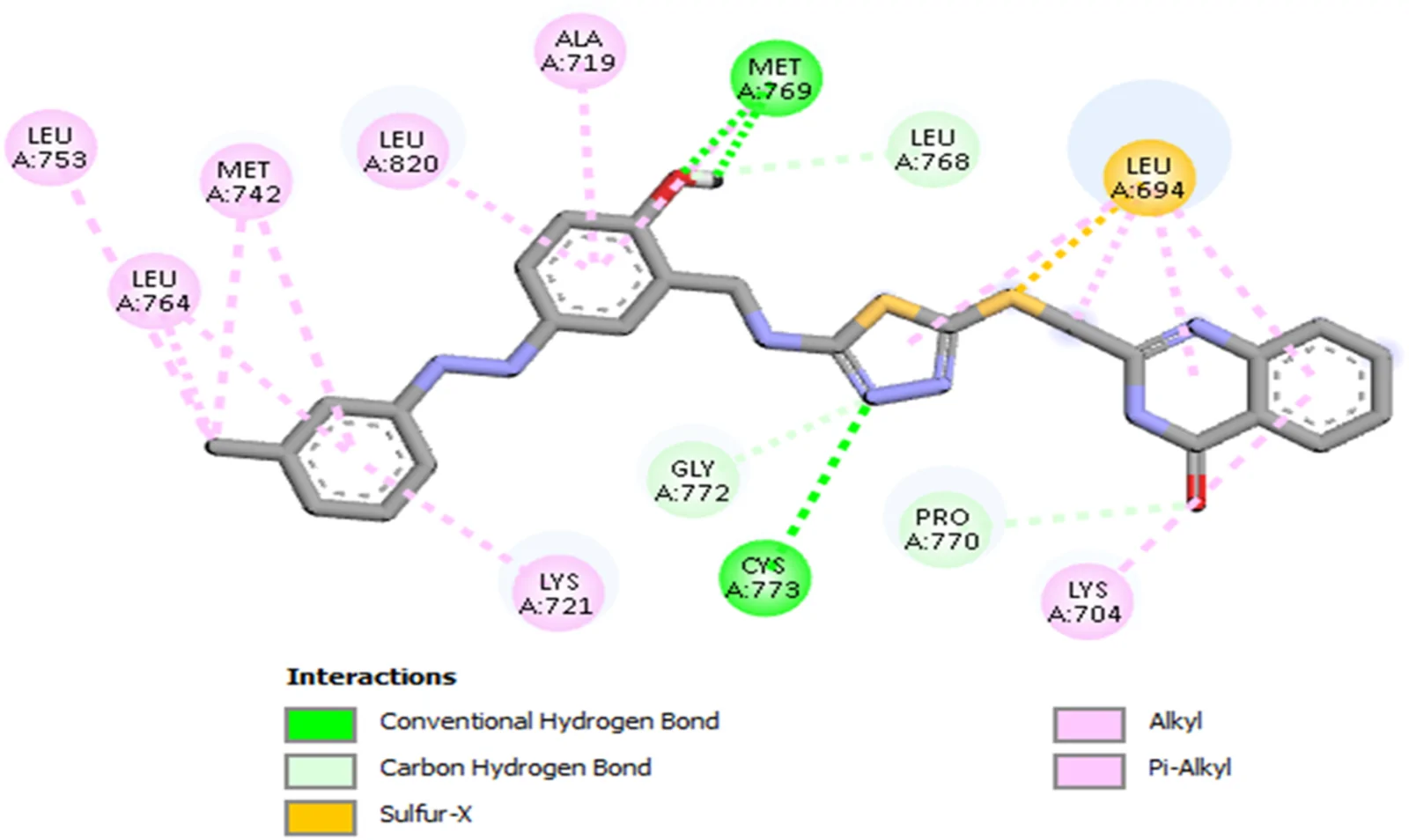
The two-dimensional suggested binding mode of compound 15 inside the EGFR active site.

**Fig. 14:**
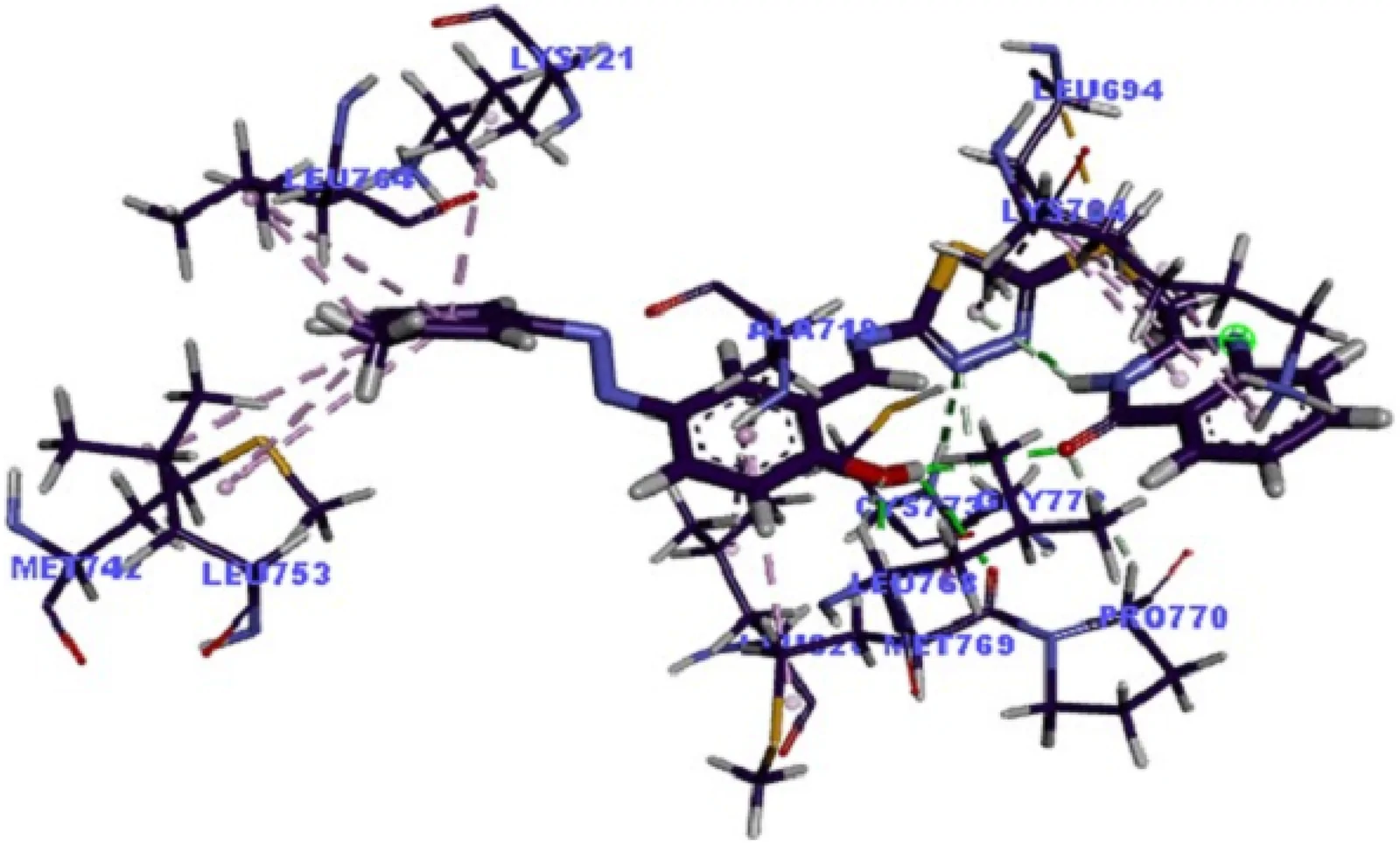
The three-dimensional suggested binding mode of compound 15 inside the EGFR active site.

#### 2.2.7. *In silico* prediction of physicochemical and pharmacokinetic properties

Both physicochemical and pharmacokinetic properties of the most active compounds 5 and 17 were estimated using SwissADME^55^Table 7.

**Table 7:** *In silico* physicochemical and pharmacokinetic properties of compounds 7 and 15

Parameter	Comp. 7	Comp. 15	Optimal range/criteria
Formula	C_16_H_13_N_5_OS_2_	C_25_H_19_N_7_O_2_S_2_	—
(MW)	355.44 g mol^−1^	513.59 g mol^−1^	≤500 g mol^−1^
Consensus log *P*	4.09	5.19	≤5.0
TPSA (Å^2^)	150.13	182.38	≤140 Å^2^
H-bond D/A	1/6	2/8	≤5 HBD/≤10 HBA
Rotatable bonds	4	7	≤10
Solubility (ESOL class)	Moderately soluble	Poorly soluble	Soluble/moderately soluble
GI absorption	Low	Low	High
BBB permeability	No	No	Depends on target (yes/no)
P-gp substrate	No	No	No (to avoid efflux)
CYP inhibitors	CYP2C19, CYP2C9	None	No inhibition (minimizes DDI)
Lipinski violations	0	1	≤1
Veber violations	1 (TPSA > 140)	1 (TPSA > 140)	0
Synthetic accessibility	3.47	4.15	1 (easy)–10 (difficult)

##### 2.2.7.1. Physicochemical properties and drug likeness

Compound 7 exhibits favorable basic drug-likeness with zero Lipinski violations (MW = 355.44, consensus log *P* = 4.09). It violates Veber's rule due to a high topological polar surface area (TPSA = 150.13 Å^2^ > 140 Å^2^), which correlates with limited oral membrane permeability. Whereas, concerning compound 5, the derivatization with the quinazolinone-like core significantly increases molecular weight (513.59 g mol^−1^) and lipophilicity (consensus log *P* = 5.19), leading to 1 Lipinski violation (MW > 500, XLOGP3 = 6.49) and 1 Veber violation (TPSA = 182.38 Å^2^).

##### 2.2.7.2. Pharmacokinetics and ADME profile

Regarding absorption and permeability, both molecules exhibit low gastrointestinal (GI) absorption and are non-permeant to the blood–brain barrier (BBB). This is primarily driven by their high polar surface areas (TPSA > 140 Å^2^) and rigid aromatic architecture.

Also, neither compound is predicted to be a P-glycoprotein (P-gp) substrate, suggesting they are not subject to P-gp-mediated active efflux. Moreover, compound 7 acts as an inhibitor of CYP2C19 and CYP2C9, pointing to potential drug–drug interaction (DDI) risk for liver metabolism pathways involving these enzymes. While compound 15 exhibits no inhibition across all five major CYP isoforms (1A2, 2C19, 2C9, 2D6, 3A4), indicating a lower metabolic clearance interference risk. Finally, considering the synthetic accessibility, compound 7 (SA score = 3.47) and compound 15 (SA score = 4.15) both fall in the easy-to-moderate synthesis range (SA < 5), confirming that chemical synthesis and structural modification in the laboratory are straightforward.

## 3. Conclusion

A novel series of 1,3,4-thiadiazole derivatives was synthesized and assessed for its cytotoxic effects *in vitro* against MCF-7, HepG-2, and HCT-116 cancer cell lines. Four derivatives, specifically 7, 8, 15, and 16, exhibited significant antitumor efficacy against the evaluated cancer cell lines. The inhibitory potencies of these compounds on VEGFR-2 and EGFR enzymes were examined, showing notable enzymatic sensitivity, particularly for compounds 7 and 15, which exhibited IC50 values of 0.17 and 0.16 µM, respectively, against VEGFR-2, and 0.42 and 0.23 µM against EGFR. Furthermore, they exhibited preferential cytotoxicity towards cancer cells in contrast to normal WI-38 cells. Molecular docking analysis demonstrated significant affinity for the ATP active site of both VEGFR-2 and EGFR enzymes. While these *in vitro* enzymatic and docking results point toward a possible VEGFR/EGFR mediated inhibition mechanism, further comprehensive studies (such as broader kinome profiling and downstream signaling analysis) will be necessary to fully rule out off target effects. Nonetheless, based on the aforementioned findings, compounds 7 and 15 emerge as promising candidates for the development of novel targeted anticancer therapeutics.

## 4. Experimental section

### 4.1. Materials and instrumentation

Unless otherwise noted, all the required chemical reagents and solvents were obtained from known commercial sources and used without further refinement. The melting point of the products obtained was measured at the SMP50 Digital Melting Point APP (Bibby Scientific, Staffordshire, UK) 120/230 V instrument, which was used to measure the melting points (mp) and was uncorrected. The prepared compounds' infrared (IR) spectra were registered with an FT-IR Spectrometer (Nicolet iS50 Spectrometer, Thermo Fisher Scientific, Resolution 16, using the potassium bromide disc method, and the outcomes are reported in (*v* cm^−1^). Nuclear magnetic resonance ^1^H NMR spectra were taken at 300 MHz and referenced to the residual solvent signal (*δ* = 2.50 ppm for DMSO-*d*_6_). ^13^C NMR spectra were obtained at 75 MHz and referenced to the residual solvent signal (*δ* = 39.52 ppm for DMSO-*d*_6_). ^1^H NMR data are described as follows: the chemical shift (delta, *δ*) in part per million, multiplicity (s = singlet, d = doublet, t = triplet, and m = multiplet), integration, and assignment. Mass spectra were defined at the Thermo Scientific GCMS model (ISQ LT) using Thermo X-Calibur software (Shimadzu, Kyoto, Japan) at the Regional Center for Mycology and Biotechnology (RCMB), Al-Azhar University, Nasr City, Cairo, Egypt. The elemental analysis was carried out at the Microanalytical Center, Cairo University, Giza, Egypt, using an Elemental C–H–N–S Analyzer, Vario El M, Germany. All the data were within ±0.15% of the theoretical values.

### 4.2. Organic synthesis

#### 4.2.1. Synthesis of 5-amino-1,3,4-thiadiazole-2-thiol 1 (ref. 43–46)

##### 4.2.1.1. General procedure for synthesis of Schiff base derivatives

A suspension of 5-amino-1,3,4-thiadiazole-2-thiol (1) (1.1 eq.) in absolute C_2_H_5_OH (20 mL) was subjected to the addition of various aromatic aldehydes (2a–g) (1.1 eq.) under acidic conditions (3 drops of concentrated H_2_SO_4_). The reaction mixture underwent reflux for 8 hours and was continuously monitored by TLC until the completion of the reaction. Upon the termination of the reaction, the mixture underwent a cooling process until it reached room temperature and was left undisturbed throughout the night. Precipitates were obtained from 1,3,4-thiadiazole derivatives incorporating Schiff base moieties. The solids were filtered and washed with a mixture of ethanol and hot water in a 1 : 1 ratio. After that, the solid was dried and purified by crystallization to produce the desired compound by crystallizing it from the proper solvent.

###### 4.2.1.1.1 (*E*)-4-Chloro-3-(((5-mercapto-1,3,4-thiadiazol-2-yl)imino)methyl)-2*H*-chromen-2-one (3)

Yield: 89% (dioxane); red powder; m.p.: 220–222 °C; FT-IR (KBr, *v* cm^−1^): 3070 cm^−1^ (Ar–H), 1713 cm^−1^ (C=O), and 1622 cm^−1^ (C=N). ^1^H NMR (300 MHz, DMSO-*d*_6_) *δ*_H_/ppm: 14.94 (s, 1H, SH), 8.80 (s, 1H, azomethine (CH=N)), 7.96–7.38 (m, 4H, Ar–H). EI-MS (*m*/*z*, %): 322 (M^+^, 3.26), and base peak 121.10 (100%); anal. calcd. For C_12_H_6_ClN_3_O_2_S_2_ (322.96): C, 44.52; H, 1.87; N, 12.98; found: C, 44.40; H, 1.75; N, 12.87%.

###### 4.2.1.1.2 (*E*)-3-(1-((5-Mercapto-1,3,4-thiadiazol-2-yl)imino)ethyl)-2*H*-chromen-2-one (4)

Yield: 85% (ethanol); red powder; m.p.: 223–225 °C, FT-IR (KBr, *v* cm^−1^): 3044 cm^−1^ (Ar–H), 2927, 2875 cm^−1^ (Alph.-H), 1674 cm^−1^ (C=O), and 1620 cm^−1^ (C=N). ^1^H NMR (300 MHz, DMSO-*d*_6_) *δ*/ppm: 14.97 (s, 1H, SH), 8.64 (s, 1H cumarin-H_(4)_), 7.96–7.39 (m, 4H, Ar–H), and 2.58 (s, 3H, CH_3_). ^13^C NMR (75 MHz, DMSO-*d*_6_) *δ*_C_/ppm: 195.02, 179.65, 166.19, 158.40, 154.56, 147.04, 134.45, 129.50, 125.97, 124.30, 118.10, 116.06, and 30.04. Anal. calcd. For C_12_H_6_ClN_3_O_2_S_2_ (303.35): C, 51.47; H, 2.99; N, 13.85; found: C, 51.36; H, 2.88; N, 13.74%.

###### 4.2.1.1.3 (*E*)-3-(((5-Mercapto-1,3,4-thiadiazol-2-yl)imino)methyl)-4*H*-chromen-4-one (5)^56^

Yield 90% (dioxane); red powder; m.p.: 234–236 °C, FT-IR (KBr, *v* cm^−1^): 3017 cm^−1^ (Ar–H), 1642 cm^−1^ (C=O), and 1607 cm^−1^ (C=N). ^1^H NMR (300 MHz, DMSO-*d*_6_) *δ*_H_/ppm: 13.42 (s, 1H, SH), 10.12 (s, 1H, chromenone-H_(2)_), 8.91 (s, 1H, azomethin (CH=N)), 8.15 (dd, *J* = 7.9, 1.7 Hz, 1H, chromenone-H_(5)_), 7.88 (ddd, *J* = 8.7, 7.1, 1.7 Hz, 1H, chromenone-H_(7)_), 7.75 (dd, *J* = 8.5, 1.1 Hz, 1H, chromenone-H_(6)_), 7.58 (ddd, *J* = 8.0, 7.1, 1.1 Hz, 1H, chromenone-H_(8)_). ^13^C NMR (75 MHz, DMSO) *δ*_C_/ppm: 188.35, 174.87, 163.35, 158.13, 155.72, 155.57, 135.16, 126.68, 125.28, 124.62, 119.95, and 118.86. EI-MS (*m*/*z*, %): 289 (M^+^, 58.76), and base peak 64.05 (100%); anal. calcd. For C_12_H_7_N_3_O_2_S_2_ (289.33): C, 49.82; H, 2.44; N, 14.52; found: C, 49.70; H, 2.33; N, 14.39%.

###### 4.2.1.1.4 4-((*E*)-(4-Fluorophenyl)diazenyl)-2-((*E*)-((5-mercapto-1,3,4-thiadiazol-2-yl)imino)methyl) phenol (6)

Yield 82% (dioxane); red powder; m.p.: >300 °C, FT-IR (KBr, *v* cm^−1^): 3414 cm^−1^ (OH), 3073 cm^−1^ (Ar–H), 1618 cm^−1^ (C=N), and 1498 cm^−1^ (N=N). ^1^H NMR (300 MHz, DMSO-*d*_6_) *δ*_H_/ppm: 13.16 (s, 1H, SH), 10.37 (s, 1H, OH), 9.16 (d, *J* = 4.2 Hz, 1H, Ar–H), 9.10–8.96 (m, 1H, azomethin (CH=N)), 8.22–8.11 (m, 2H, Ar–H), 7.93 (s, 2H, Ar–H), 7.35 (s, 1H, ArH), 7.15 (s, 1H, Ar–H). EI-MS (*m*/*z*, %): 360 (M^+^ + 1, 9.51), and base peak 59.10 (100%); anal. calcd. For C_15_H_10_FN_5_OS_2_ (359.397): C, 50.13; H, 2.80; N, 19.49; found: C, 50.02; H, 2.68; N, 19.38%.

###### 4.2.1.1.5 2-((*E*)-((5-Mercapto-1,3,4-thiadiazol-2-yl)imino)methyl)-4-((*E*)-*m*-tolyldiazenyl)phenol (7)

Yield 77% (dioxane); red powder; m.p.: 232–234 °C, FT-IR (KBr, *v* cm^−1^): 3410 cm^−1^ (OH), 3056 cm^−1^ (Ar–H), 2927 cm^−1^ (Alph.-H), 1614 cm^−1^ (C=N), and 1491 cm^−1^ (N=N). ^1^H NMR (300 MHz, DMSO-*d*_6_) *δ*_H_ (ppm): 13.18 (s, 1H, SH), 10.36 (s, 1H, OH), 9.15 (s, 1H, Ar–H), 8.15 (s, 1H, azomethin (CH=N)), 7.65 (s, 2H, Ar–H), 7.44 (s, 2H, Ar–H), 7.33 (s, 2H, Ar–H), 7.15 (s, 1H, Ar–H), 2.41–2.40 (m, 3H, CH_3_). ^13^C NMR (75 MHz, DMSO-*d*_6_) *δ*_C_/ppm: 190.37, 172.03, 163.52, 161.57, 151.90, 144.70, 138.92, 131.77, 129.68, 129.23, 123.32, 122.63, 122.44, 120.01, 118.48, and 20.95; HPLC purity 95.17%; EI-MS (*m*/*z*,%): 355 (M^+^, 0.75), and base peak 91.10 (100%); anal. calcd. For C_16_H_13_N_5_OS_2_ (355.434): C, 54.07; H, 3.69; N, 19.70; O, found: C, 53.95; H, 3.58; N, 19.58%.

###### 4.2.1.1.6 4-((*E*)-(3-Chlorophenyl)diazenyl)-2-((*E*)-((5-mercapto-1,3,4-thiadiazol-2-yl)imino)methyl) phenol (8)

Yield 86% (dioxane); red powder; m.p.: 240–242 °C; FT-IR (KBr, *v* cm^−1^): 3353 cm^−1^ (OH), 3064 cm^−1^ (Ar–H), 1611 cm^−1^ (C=N), and 1487 cm^−1^ (N=N). ^1^H NMR (300 MHz, DMSO-*d*_6_) *δ*_H_/ppm: 13.16 (s, 1H, SH), 11.80 (s, 1H, OH), 10.35 (s, 1H, Ar–H), 8.21–8.10 (m, 1H, azomethin (CH=N)), 7.79 (s, 3H, Ar–H), 7.66 (s, 1H, Ar–H), 7.56 (s, 1H, Ar–H), 7.30 (s, 1H, Ar–H), 7.12 (s, 1H, Ar–H); HPLC purity 100%; EI-MS (*m*/*z*, %): 375, 376 (M^+^, 7.25, M^+^ + 1, 6.44), and base peak 111.05 (100%); anal. calcd. For C_15_H_10_ClN_5_OS_2_ (375.85): C, 47.94; H, 2.68; N, 18.63; found: C, 47.83; H, 2.57; N, 18.51%.

###### 4.2.1.1.7 4-((*E*)-(4-Chlorophenyl)diazenyl)-2-((*E*)-((5-mercapto-1,3,4-thiadiazol-2-yl)imino)methyl) phenol (9)^57^

Yield 87% (dioxane); red powder; m.p.: > 300 °C, FT-IR (KBr, *v* cm^−1^): 3413 cm^−1^ (OH), 3090 cm^−1^ (Ar–H), 1617 cm^−1^ (C=N), and 1482 cm^−1^ (N=N). ^1^H NMR (300 MHz, DMSO-*d*_6_) *δ*_H_/ppm: 13.18 (s, 1H, SH), 10.36 (s, 1H, OH), 9.13 (s, 1H, Ar–H), 8.31 (s, 1H), 8.19 (d, *J* = 3.00 Hz, 1H, Ar–H), 7.86 (d, *J* = 9.00 Hz, 2H, Ar–H), 7.65 (d, *J* = 6.00 Hz, 2H, Ar–H), 7.22 (d, *J* = 9.00 Hz, 1H, Ar–H), 7.07 (s, 1H, Ar–H). ^13^C NMR (75 MHz, DMSO) *δ*_C_/ppm: 190.38, 180.88 (C=N), 163.62, 161.50, 150.42, 144.64, 135.39, 129.79, 127.81, 127.45, 124.03, 123.94, 123.31, 122.66, and 118.48. EI-MS (*m*/*z*, %): 375 (M^+^, 2.40), and base peak 111.10 (100%); anal. calcd. For C_15_H_10_ClN_5_OS_2_ (375.00): C, 47.94; H, 2.68; N, 18.63; O. Found: C, 47.81; H, 2.56; N, 18.52%.

###### 4.2.1.1.8 2-(Chloromethyl)quinazolin-4(3*H*)-one (10)

Sodium methoxide (cat.) [made from sodium pieces dissolved in methanol, and the solvent was evaporated under reduced pressure to give a white solid] was added at RT into a dried round-bottom flask charged with a stir bar and dropping funnel. The title compound was prepared in accordance with the procedure previously published in the literature,^51^ with minor modifications. After that, an ice bath was added to the flask. Concurrently, chloroacetonitrile (1 mmol) was syringed dropwise into the resulting mixture over a period of 15 min at 0 °C. Afterward, crude *o*-anthranilic acid in methanol was added sequentially and dropwise. The resulting mixture was then subjected to an additional 45-minute stirring period at the same temperature, followed by continuous stirring at room temperature. The major product was rapidly generated as a precipitated solid, and the mixture was filtered and washed with cool water and then left to dry at room temperature. As a result, the corresponding 2-(chloromethyl)quinazoline-4(3*H*)-one (10) was obtained as a white solid; yield: 40%; mp: 249 °C [lit. m.p. 249–250 °C].

##### 4.2.1.2. General method for synthesis of compounds (11–17)

A catalytic amount of anhydrous potassium hydroxide was solubilized in absolute ethanol (30 mL) in a 100 mL one-necked round-bottom flask. The thiadiazole derivatives (3–9) (0.01 mol) were subsequently placed into the flask. The resulting mixture was agitated for 1 hour under reflux conditions, with the reaction mixture temperature consistently maintained in the range of 55° to 60°. Next, the same amount of 2-(chloromethyl)quinazolin-4(3*H*)-one (10) was steadily incorporated dropwise over five minutes. The flask contents were subjected to reflux overnight with stirring. Subsequently, the solid product underwent filtration, followed by washing with cold ethanol and drying in a vacuum oven for 24 hours, resulting in the production of the desired compounds (11–17).

###### 4.2.1.2.1 (*E*)-2-(((5-(((4-Chloro-2-oxo-2*H*-chromen-3-yl)methylene)amino)-1,3,4-thiadiazol-2-yl)thio) methyl)quinazolin-4(3*H*)-one (11)

Yield 78% (dioxane + EtOH); red powder; m.p.: >300 °C, FT-IR (KBr, *ν* cm^−1^): 3316 cm^−1^ (NH), 3069 cm^−1^ (Ar–H), 2923 cm^−1^ (Aliph.-H), 1713, 1661 cm^−1^ (2C=O), and 1606 cm^−1^ (C=N). ^1^H NMR (300 MHz, DMSO-*d*_6_) *δ*_H_/ppm: 12.92 (s, 1H, NH), 8.77 (s, 1H, CH=N), 8.18–7.38 (m, 8H, Ar–H), and 3.66 (s, 2H, CH_2_). EI-MS (*m*/*z*, %): 481 (M^+^, 0.90), and base peak 346.20 (100%); anal. calcd. For C_21_H_12_ClN_5_O_3_S_2_ (481.929): C, 52.34; H, 2.51; N, 14.53; found: C, 52.22; H, 2.40; N, 14.42%.

###### 4.2.1.2.2 (*E*)-2-(((5-((1-(2-Oxo-2*H*-chromen-3-yl)ethylidene)amino)-1,3,4-thiadiazol-2-yl)thio)methyl) quinazolin-4(3*H*)-one (12)

Yield 74% (dioxane + EtOH); red powder; m.p.: 292–294 °C, FT-IR (KBr, *ν* cm^−1^): 3334 cm^−1^ (NH), 3029 cm^−1^ (Ar–H), 2925 cm^−1^ (Aliph.-H), 1739, 1678 cm^−1^ (2C=O), and 1611 cm^−1^ (C=N). ^1^H NMR (300 MHz, DMSO-*d*_6_) *δ*_H_/ppm: 12.37 (s, 1H, NH), 8.10–7.33 (m, 9H, 1H, coumarin-4H& 8H-Ar-H), 4.19 (s, 1H, CH_2_), 2.50 (s, 3H, CH_3_). ^13^C NMR (75 MHz, DMSO) *δ*_C_/ppm: 174.52, 170.74, 161.54, 153.11, 148.34, 148.26, 147.10, 134.56, 129.34, 127.47, 126.99, 126.83, 126.75, 126.51, 126.08, 125.85, 121.02, 121.01, 116.09, 110.22, 37.71, and 22.66. EI-MS (*m*/*z*, %): 461 (M^+^, 0.12), and base peak 291.10 (100%); anal. calcd. For C_22_H_15_N_5_O_3_S_2_ (461.514): C, 57.26; H, 3.28; N, 15.18. Found: C, 57. p; H, 3.19; N, 15.05%.

###### 4.2.1.2.3 (*E*)-2-(((5-(((4-Oxo-4*H*-chromen-3-yl)methylene)amino)-1,3,4-thiadiazol-2-yl)thio)methyl) quinazolin-4(3*H*)-one (13)

Yield 84% (dioxane + EtOH); red powder; m.p.: 277–279 °C, FT-IR (KBr, *ν* cm^−1^): 3383 (NH), 3079 (Ar–H), 2977 (Aliph.-H), 1676 (C=O), and 1621 (C=N). ^1^H NMR (300 MHz, DMSO-*d*_6_) *δ*_H_/ppm: 13.15 (s, 1H, NH), 10.26 (s, 1H, chromenone-H), 8.82 (s, 1H, azomethin (CH=N)), 8.26 (d, *J* = 8.2 Hz, 1H, Ar–H), 8.09 (d, *J* = 8.3 Hz, 1H, Ar–H), 8.01–7.90 (m, 1H, Ar–H), 7.78 (t, *J* = 8.8 Hz, 2H, Ar–H), 7.58 (d, *J* = 8.2 Hz, 1H, Ar–H), 7.49 (t, *J* = 7.6 Hz, 1H, Ar–H), 7.10 6.91 (m, 1H, Ar–H), 4.19 (s, 2H, CH_2_). ^13^C NMR (75 MHz, DMSO) *δ*_C_/ppm: 173.30, 170.71, 161.49, 158.19, 155.97, 153.08, 148.29, 146.56, 136.02, 134.53, 133.25, 130.63, 129.92, 127.09, 126.95, 126.72, 126.53, 125.82, 121.80, 119.22, 37.67. EI-MS (*m*/*z*, %): 447 (M^+^, 2.70), and base peak 60.00 (100%); anal. calcd. For C_21_H_13_N_5_O_3_S_2_ (447.487): C, 56.37; H, 2.93; N, 15.65; found: C, 56.25; H, 2.83; N, 15.51%.

###### 4.2.1.2.4 2-(((5-(((*E*)-5-((*Z*)-(4-Fluorophenyl)diazenyl)-2-hydroxybenzylidene)amino)-1,3,4-thiadiazol-2-yl)thio)methyl)quinazolin-4(3*H*)-one (14)

Yield 81% (dioxane + EtOH); red powder; m.p.: >300 °C, FT-IR (KBr, *ν* cm^−1^): 3382 (OH), 3179 (NH), 3045 (Ar–H), 2976, 2876 (Aliph.-H), 1673 (C=O), 1619 (C=N), and 1522 (N=N). ^1^H NMR (300 MHz, DMSO-d_6_) *δ*_H_/ppm: 12.37 (s, 1H, OH), 10.48 (s, 1H, NH), 8.17–8.05 (m, 2H, azomethine (CH=N) and Ar–H), 7.86–7.74 (m, 2H, Ar–H), 7.65–7.55 (m, 2H, Ar–H), 7.56–7.45 (m, 3H, Ar–H), 7.33 (s, 2H, Ar–H), 6.99 (d, *J* = 51.6 Hz, 1H, Ar–H), 4.19 (s, 2H, CH_2_). ^13^C NMR (75 MHz, DMSO) *δ*_C_/ppm: 180.29, 170.74, 163.30, 161.53 (F–C), 153.72, 153.23, 153.09, 151.98, 148.36, 148.20, 134.59, 129.95, 129.55, 127.04, 126.78, 126.06, 125.85, 125.47, 121.08, 120.99, 120.42, 118.04, 116.61, 115.73, 37.69. EI-MS (*m*/*z*, %): 517 (M^+^, 0.61), and base peak 291.20 (100%); anal. calcd. For C_24_H_16_FN_7_O_2_S_2_ (517.557): C, 55.70; H, 3.12; N, 18.94; found: C, 56.25; H, 3.03; N, 18.82%.

###### 4.2.1.2.5 2-(((5-(((*E*)-2-Hydroxy-5-((*Z*)-*m*-tolyldiazenyl)benzylidene)amino)-1,3,4-thiadiazo-*l*-2-yl)thio) methyl)quinazolin-4(3*H*)-one (15)

Yield 76% (dioxane + EtOH); red powder; m.p.: >300 °C, FT-IR (KBr, *ν* cm^−1^): 3379 (OH), 3179 (NH), 3047 (Ar–H), 2916, 2878 (Aliph.-H), 1673 (C=O), 1613 (C=N), and 1522 (N=N). ^1^H NMR (300 MHz, DMSO-*d*_6_) *δ*_H_/ppm: 12.38 (s, 1H, OH), 10.99 (s, 1H, NH), 9.09 (s, 1H, azomethine), 8.17–6.95 (m, 11H, Ar–H & quinoxaline-H), 4.20 (s, 2H, CH_2_), 2.39 (s, 3H, CH_3_). ^13^C NMR (75 MHz, DMSO) *δ*_C_/ppm: 170.82, 169.12, 163.69, 161.62, 161.49, 159.15, 159.14, 157.82, 153.16, 153.16, 148.37, 148.34, 134.65, 127.04, 126.84, 126.30, 126.22, 126.03, 125.91, 125.82, 122.59, 121.04, 116.50, 37.78, 21.02; HPLC purity 95.75%; EI-MS (*m*/*z*, %): 513 (M^+^, 3.93), and base peak 90.10 (100%); anal. calcd. For C_25_H_19_N_7_O_2_S_2_ (513.594): C, 58.47; H, 3.73; N, 19.09; found: C, 58.36; H, 3.65; N, 18.97%.

###### 4.2.1.2.6 2-(((5-(((*E*)-5-((*Z*)-(3-Chlorophenyl)diazenyl)-2-hydroxybenzylidene)amino)-1,3,4-thiadiazol-2-yl)thio)methyl)quinazolin-4(3*H*)-one (16)

Yield 76% (dioxane + EtOH); red powder; m.p.: >300 °C, FT-IR (KBr, *v* cm^−1^): 3372 (OH), 3178 (NH), 3066 (Ar–H), 2975, 2877 (Aliph.-H), 1676 (C=O), 1608 (C=N), and 1522 (N=N). ^1^H NMR (300 MHz, DMSO-*d*_6_) *δ*_H_/ppm: 12.37 (s, 1H, OH), 10.36 (s, 1H, NH), 9.15 (s, 1H, azomethin (CH=N)), 8.20 (s, 1H, quinoxaline-H), 8.10 (d, *J* = 8.0 Hz, 1H, quinoxaline-H), 7.81 (dd, *J* = 13.4, 5.2 Hz, 2H, quinoxaline-H), 7.61 (d, *J* = 0.4 Hz, 5H, Ar–H), 7.51 (t, *J* = 7.8 Hz, 7H, Ar–H), 7.33 (s, 1H, Ar–H), 4.19 (s, 2H, CH_2_); HPLC purity 96.96%; EI-MS (*m*/*z*, %): 533, 534 (M^+^, M^+^ + 1, 15.16, 13.20), and base peak 111.10 (100%); anal. calcd. For C_24_H_16_ClN_7_O_2_S_2_ (533.050): C, 53.98; H, 3.02; N, 18.36; found: C, 53.87; H, 2.93; N, 18.24%.

###### 4.2.1.2.7 2-(((5-(((*E*)-5-((*Z*)-(4-Chlorophenyl)diazenyl)-2-hydroxybenzylidene)amino)-1,3,4-thiadiazol-2-yl)thio)methyl)quinazolin-4(3*H*)-one (17)

Yield 79% (dioxane + EtOH); red powder; m.p.: >300 °C, FT-IR (KBr, *ν* cm^−1^): 3381 (OH), 3183 (NH), 3066 (Ar–H), 2971, 2865 (Aliph.-H), 1670 (C=O), 1608 (C=N), and 1522 (N=N). ^1^H NMR (300 MHz, DMSO-*d*_6_) *δ*_H_/ppm: 11.55 (s, 1H, OH), 10.35 (s, 1H, NH), 9.08 (s, 1H, azomethine), 8.17–7.13 (m, 11H, Ar–H & quinoxaline-H), 4.20 (s, 2H, CH_2_). ^13^C NMR (75 MHz, DMSO) *δ*_C_/ppm: 190.43, 171.93, 163.58, 161.53, 150.38, 144.61, 140.35, 135.53, 134.72, 134.54, 129.51, 126.96, 125.96, 125.86, 124.00, 123.93, 122.62, 120.51, 119.32, 118.43, 30.42. EI-MS (*m*/*z*, %): 534, 535, 536 (M^+^, M^+^ + 1, M^+^ + 2, 43.39, 17.07, 32.57), and base peak 111.05 (100%); anal. calcd. For C_24_H_16_ClN_7_O_2_S_2_ (534.009): C, 53.98; H, 3.02; N, 18.36; found: C, 53.88; H, 2.94; N, 18.23%.

### 4.3. Biological evaluation

#### 4.3.1. *In vitro* antitumor assay

##### 4.3.1.1. Methodology

The cancer cell lines MCF-7, HepG-2, and HCT-116, along with the normal cell line WI-38, were sourced from the American Type Culture Collection. They were cultured in Dulbecco's Modified Eagle's Medium (Life Technologies), which supports their growth. The culture medium was supplemented with 10% fetal bovine serum (FBS) from HyClone, 10 µg mL^−1^ of insulin (Sigma), and 1% penicillin/streptomycin. Additional chemicals and reagents were produced from Invitrogen or Sigma. Seed cells, at a density of 1.2–1.8 × 10 000 cells per well, were placed in a 96-well plate containing 100 µL of complete growth medium and 100 µL of the tested chemical per well. The cells were incubated for 24 hours prior to performing the MTT assay.

##### 4.3.1.2. MTT assay

The cytotoxic effect was evaluated with the MTT assay. The mature cells underwent trypsin treatment and subsequent quantification and were then seeded at appropriate densities (2000–1000 cells/0.33 cm^2^ well) in 96-well microtiter plates. The cells were subsequently placed in a controlled environment with elevated humidity for 24 hours at a temperature of 37 °C. Subsequently, the cells were exposed to varying doses of chemicals (0.01, 0.1, 1.0, 10, and 100 µM) for 48 hours. Subsequently, the MTT assay was utilized to evaluate the viability of the treated cells as follows. The medium was removed, and the cells were subsequently treated with 200 µL of a 5% MTT solution per well (supplied by Sigma-Aldrich). The cells were allowed 2 hours to metabolically transform the dye into colored, insoluble formazan crystals. The remaining MTT solution was removed from the wells, and the formazan crystals were dissolved in 200 µL of acidified isopropyl alcohol for 30 minutes. The procedure was conducted at ambient temperature with continuous agitation, while the wells were shielded with aluminum foil. The absorbance of each well was measured at a wavelength of 450 nm utilizing a Robonik P2000 EIA reader. Cell viability was assessed as a percentage of the control, and the concentration necessary to block 50% of cell proliferation (IC_50_) was calculated using GraphPad Prism software (GraphPad Software Inc.).

#### 4.3.2. *In vitro* kinese inhibition

Enzyme-linked immunosorbent assay (ELISA) technique was applied using the human VEGFR-2 ELISA kit to calculate the IC_50_ of all new derivatives against VEGFR-2 kinase. The antibody specific to the enzyme was plated on a 96-well microplate, to which different micromolar concentrations of the positive control and the new compounds were added. After incubation at 25 °C for about 2.5 h, the biotin antibody (100 µL) was added. The plates were further incubated for 1 h at 25 °C and then washed. A solution of streptavidin (100 µL) was added to the microplates, which were incubated for another 45 min at 25 °C and then washed. Similarly, a solution of tetramethylbenzidine (TMB) substrate (100 µL) was added, and the plates were incubated at 25 °C for half an hour. Finally, stop solution (50 µL) was added, and the reading was immediately determined at 450 nm. To get the standard curve, the concentrations used were presented on the *X*-axis and the corresponding absorbances were on the *Y*-axis. The inhibitory concentration 50 was derived using the concentration-inhibition response curve (*n* = 3), and IC_50_ was calculated using the GraphPad Prism 5 program.

#### 4.3.3. Docking study

The VEGFR-2 enzyme (PDB ID: 4ASD) and the EGFR enzyme (PDB ID: 1M17) were the two targets against which the molecular docking study was carried out using AutoDock Vina to assess the binding affinities of the examined compounds.

##### 4.3.3.1. Receptor and ligand preparation

Both receptors were created by eliminating all heteroatoms and co-crystallized water molecules before docking. Polar hydrogens were added, Kollman charges were computed, and the final receptor structures were transformed into the necessary Pdbqt format using MGLTools. Gasteiger partial charges were assigned, rotatable bonds were established, and the 3D structures of the examined compounds were energy-minimized before being converted to the Pdbqt format for ligand synthesis.

##### 4.3.3.2. Grid box and docking parameters

Grid boxes assessed using MGLTools were used to define the binding locations for both targets. The grid dimensions for VEGFR-2 were 40 × 40 × 40, and the grid center coordinates were set to *x* = −24.6, *y* = −0.38, and *z* = −10.9. Similarly, using the same grid size of 40 × 40 × 40, the grid center coordinates for EGFR were determined as *x* = 22.0, *y* = 0.25, and *z* = 52.7. The exhaustiveness parameter was set to 16 for both docking techniques. A maximum of nine binding poses (modes) per ligand were produced by the docking technique for both targets.

##### 4.3.3.3. Visualization and analysis

The grid box surroundings were assessed using Biovia Discovery Studio 2020 Visualizer after the docking operations, and the docking data were used to generate expected 3D and 2D binding postures for the examined compounds.

## 5. Data analysis

The percentage of cell viability was calculated as follows = [(ODt/ODc)] × 100%, where ODt is the mean optical density of wells treated with the tested compound and ODc is the mean optical density of untreated cells. The tested compounds were compared using the IC50 value, *i.e.*, the concentration of an individual compound leading to 50% cell death estimated from graphical plots of surviving cells *versus* compound concentrations, and IC_50_ was calculated using the GraphPad Prism 5 program.

## Conflicts of interest

No conflicts of interest are to be declared.

## Funding

This research work was funded by Umm Al-Qura University, Saudi Arabia under grant number: 26UQU4290565GSSR11.

## Supplementary Material

RA-OLF-D6RA07414F-s001

## Data Availability

All data generated or analyzed during this study are included in this published article and its supplementary information (SI) files. Supplementary information is available. See DOI: https://doi.org/10.1039/d6ra07414f.
