# Supplementary material for: Discovery of novel 1,3,4-thiadiazole derivatives as potential dual EGFR/VEGFR inhibitors with activity against breast, colon, and liver cancer cells: biological evaluation and molecular docking investigation
Source: RSC Adv. 2026 Nov 11. Online ahead of print. doi: 10.1039/d6ra07414f (PMC13752813; doi:10.1039/d6ra07414f)
Supplement: Supplementary file 1 [file RA-OLF-D6RA07414F-s001.pdf]

Supplementary Information (SI) for RSC Advances.
This journal is © The Royal Society of Chemistry

Discovery of Novel 1,3,4-Thiadiazole Derivatives as Potential Dual EGFR/VEGFR Inhibitors with Activity Against Breast, Colon, and Liver Cancer Cells: Biological Evaluation and Molecular Docking Investigation

Amany Belal [a], Mohammed A. S. Abourehab [b], Reem I. Alsantali [a], Ahmad J. Obaidullah [c], Randa Erfan [d], Yasmine Elwan [d], Ahmed A. Gaber [e], Ahmed B. M. Mehany [f], Rehab Sabour [g], Walid E. Elgammal [h] **

[a] Department of Pharmaceutical Chemistry, College of Pharmacy, Taif University, P.O.Box 11099,Taif 21944, Saudi Arabia

[b] Pharmaceutical Science Department College of Pharmacy Umm Al-Qura University. Makkah, 21955, Saudi Arabia

[c] Department of Pharmaceutical Chemistry, College of Pharmacy, King Saud University, P.O. Box 2457, Riyadh 11451, Saudi Arabia.

[d] Department of Medical Biochemistry and Molecular Biology, Faculty of Medicine, Cairo University, Cairo, Egypt

[e] Department of Pharmaceutical Organic Chemistry, Faculty of Pharmacy (Boys), Al-Azhar University, Cairo 11884, Egypt

[f] Zoology Department, Faculty of Science (Boys), Al-Azhar University, Nasr City, 11884, Cairo, Egypt.

[g] Department of Pharmaceutical Chemistry, Faculty of Pharmacy (Girls), Al-Azhar University, Cairo, Egypt

[h] Chemistry Department, Faculty of Science (Boys), Al-Azhar University, Nasr City, 11884, Cairo, Egypt.

Corresponding author:

**** Walid E. Elgammal:**

Chemistry Department, Faculty of Science, Al-Azhar University, Nasr City, Cairo, Egypt,

Tel: +201025267111, Email: walidebaied.sci85@azhar.edu.eg, ORCID: https://orcid.org/0000-0002-5982-5635

Supporting Information

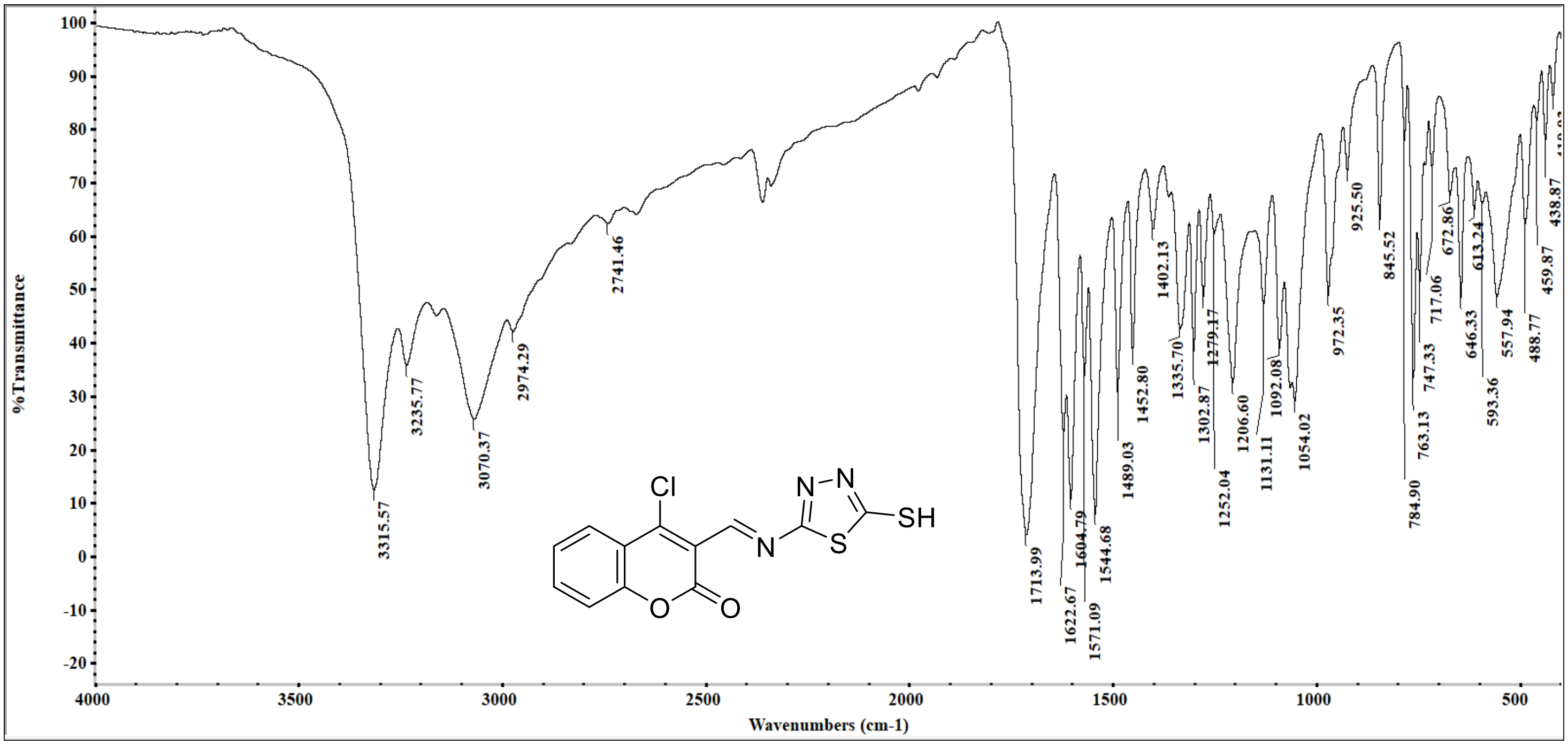

Fig.S1; FT-IR Spectrum of compound 3

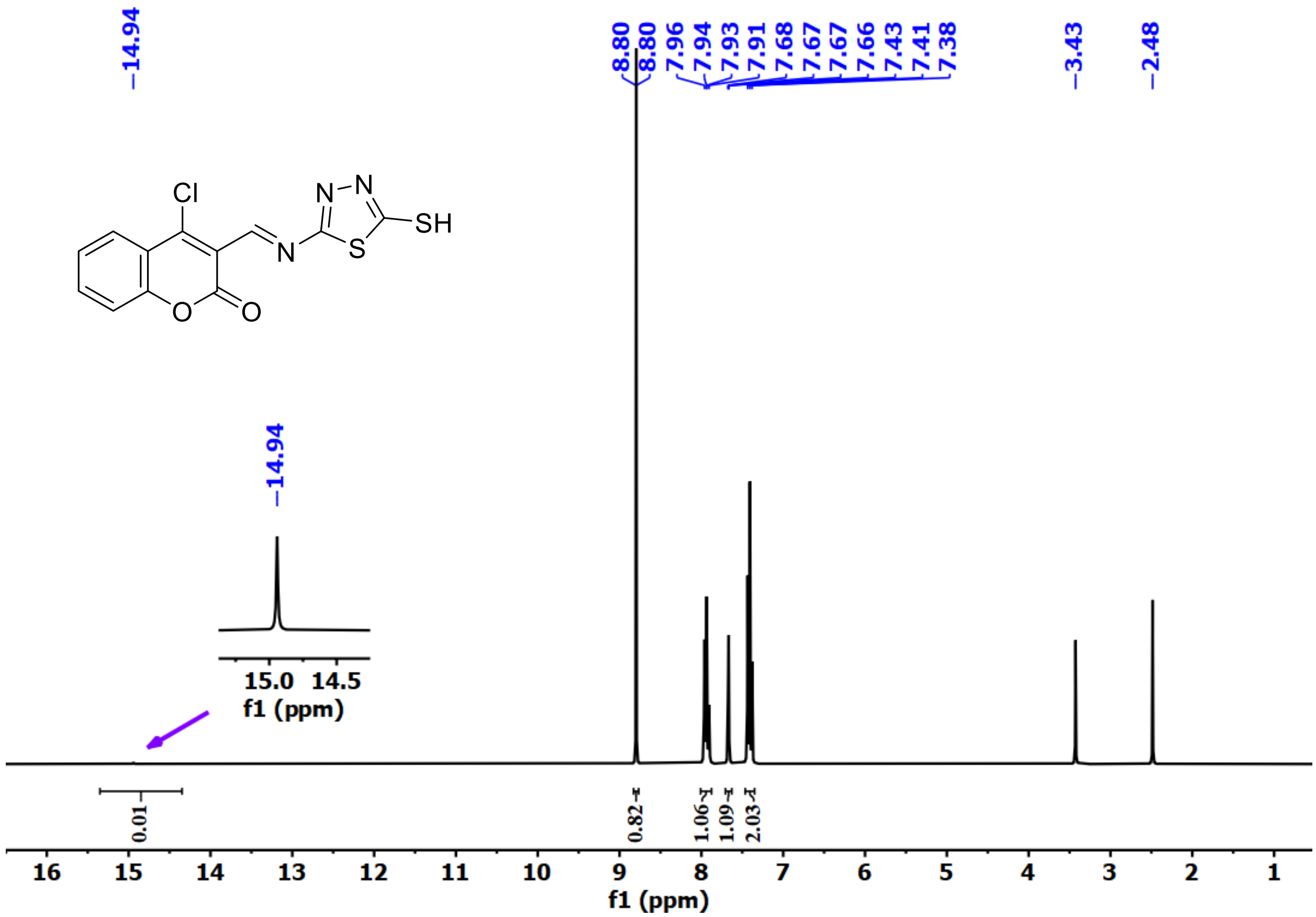

Fig. S2; ^{1}H NMR (DMSO-d_6) Spectrum of compound 3

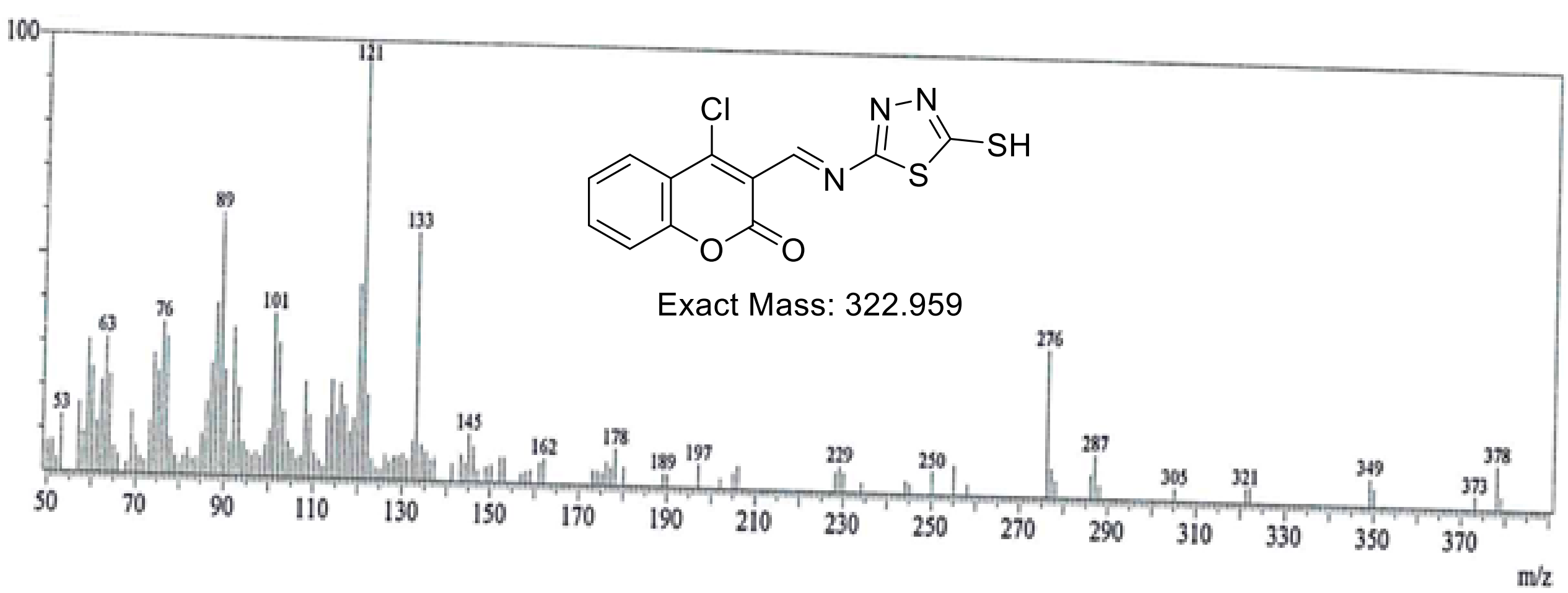

Fig. S3; Mass Spectrum of compound 3

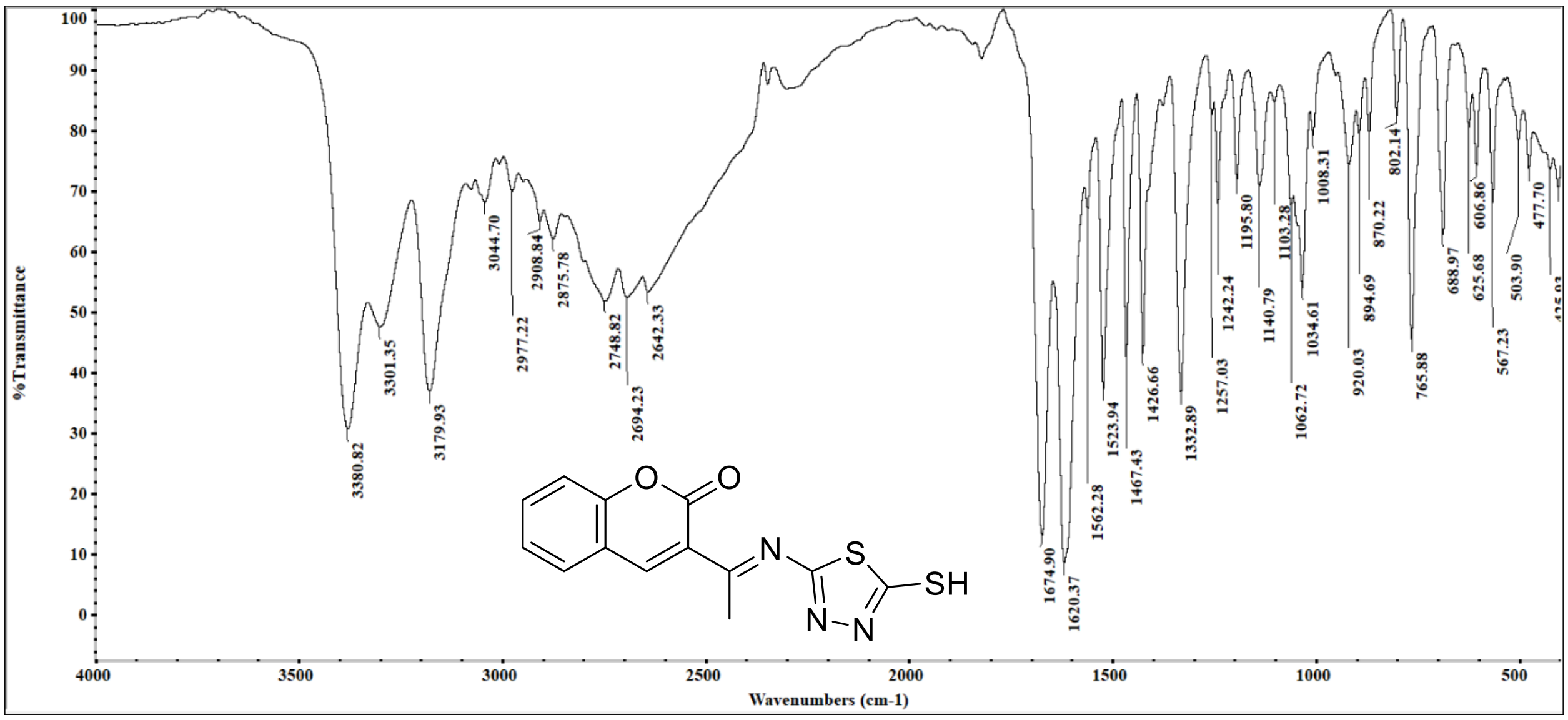

Fig. S4; FT-IR Spectrum of Compound 4

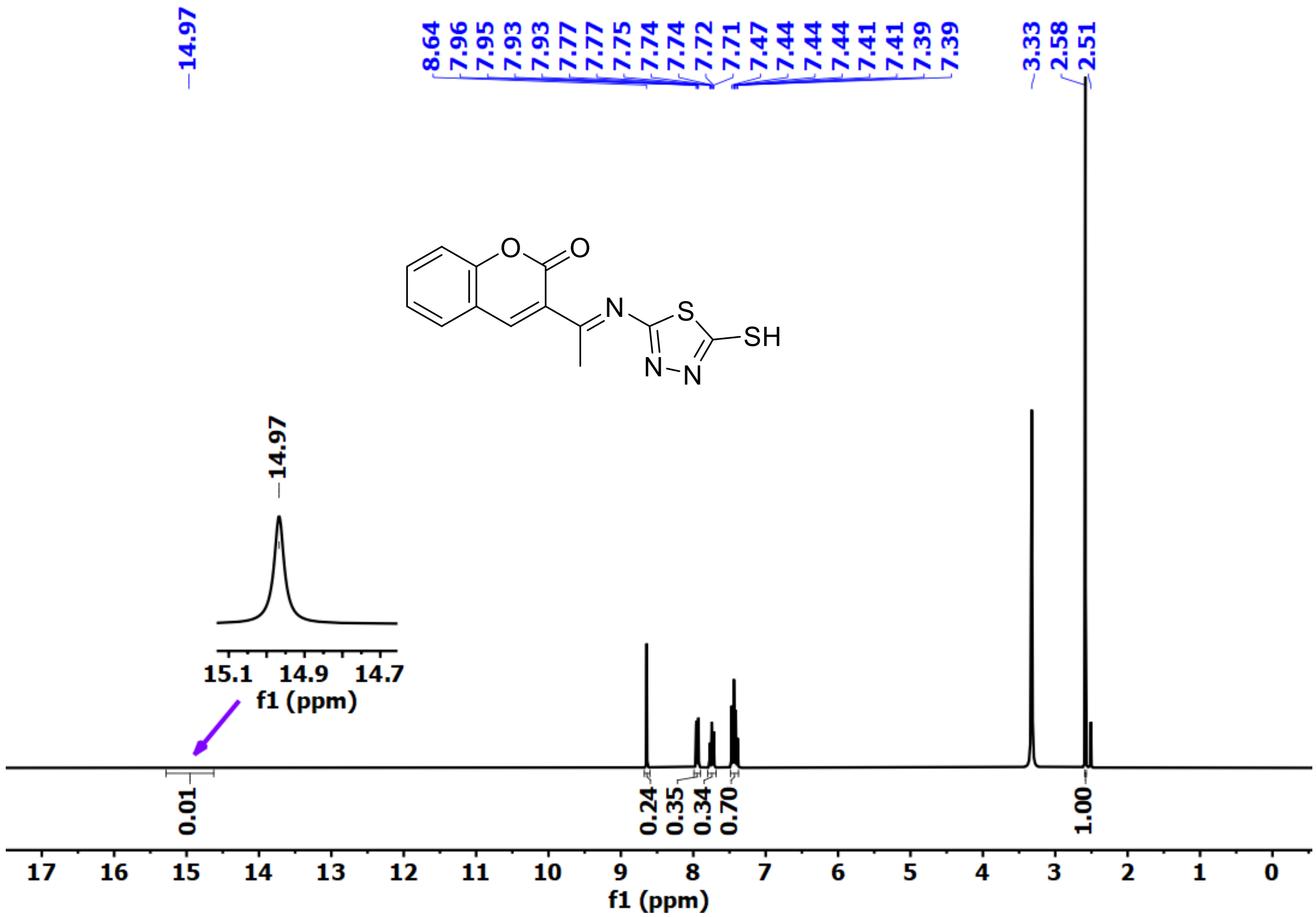

Fig. S5; ^{1}H NMR (DMSO-d$_6$) Spectrum of compound 4

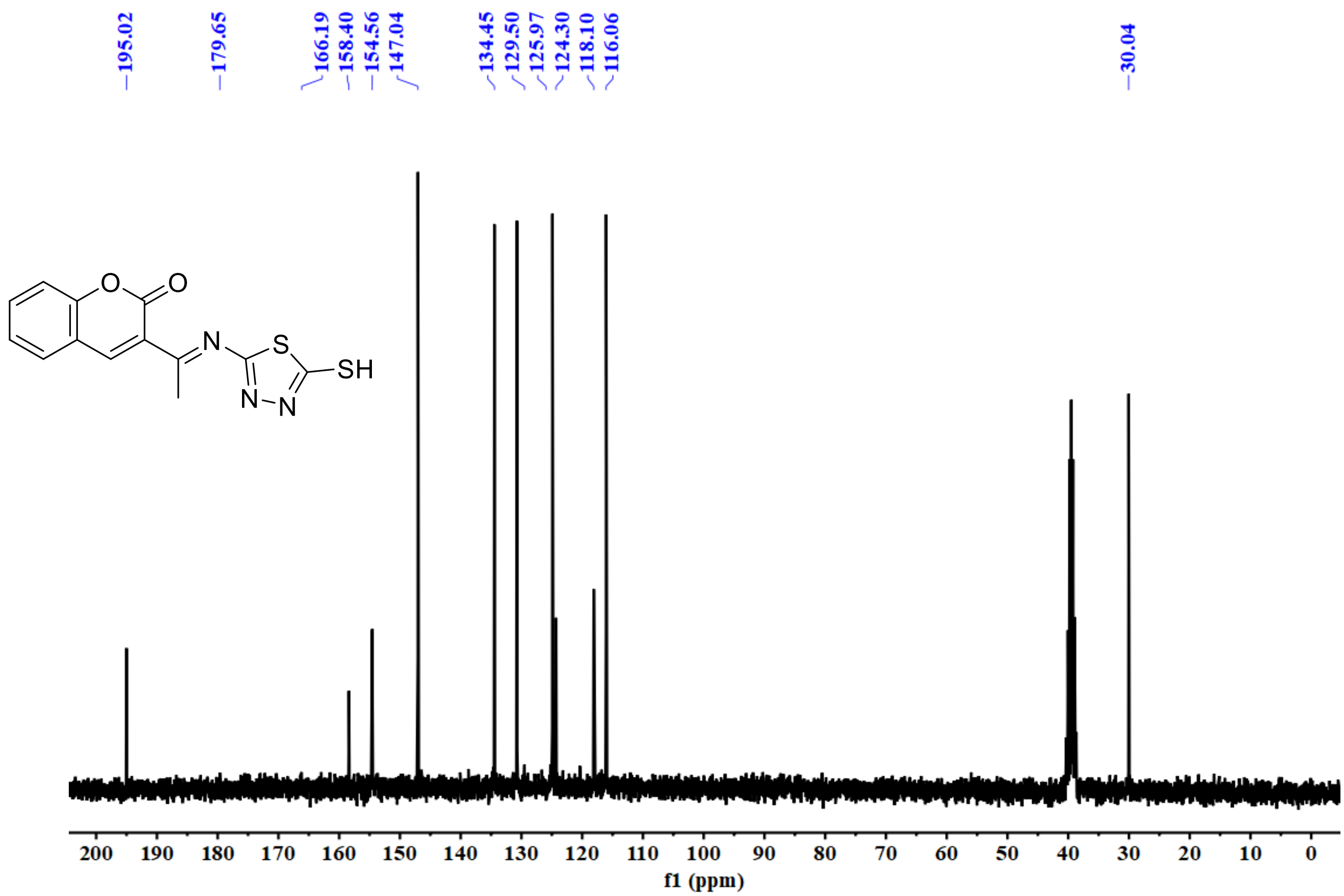

Fig. S6; ^{13}C NMR (DMSO-d_6) Spectrum of compound 4

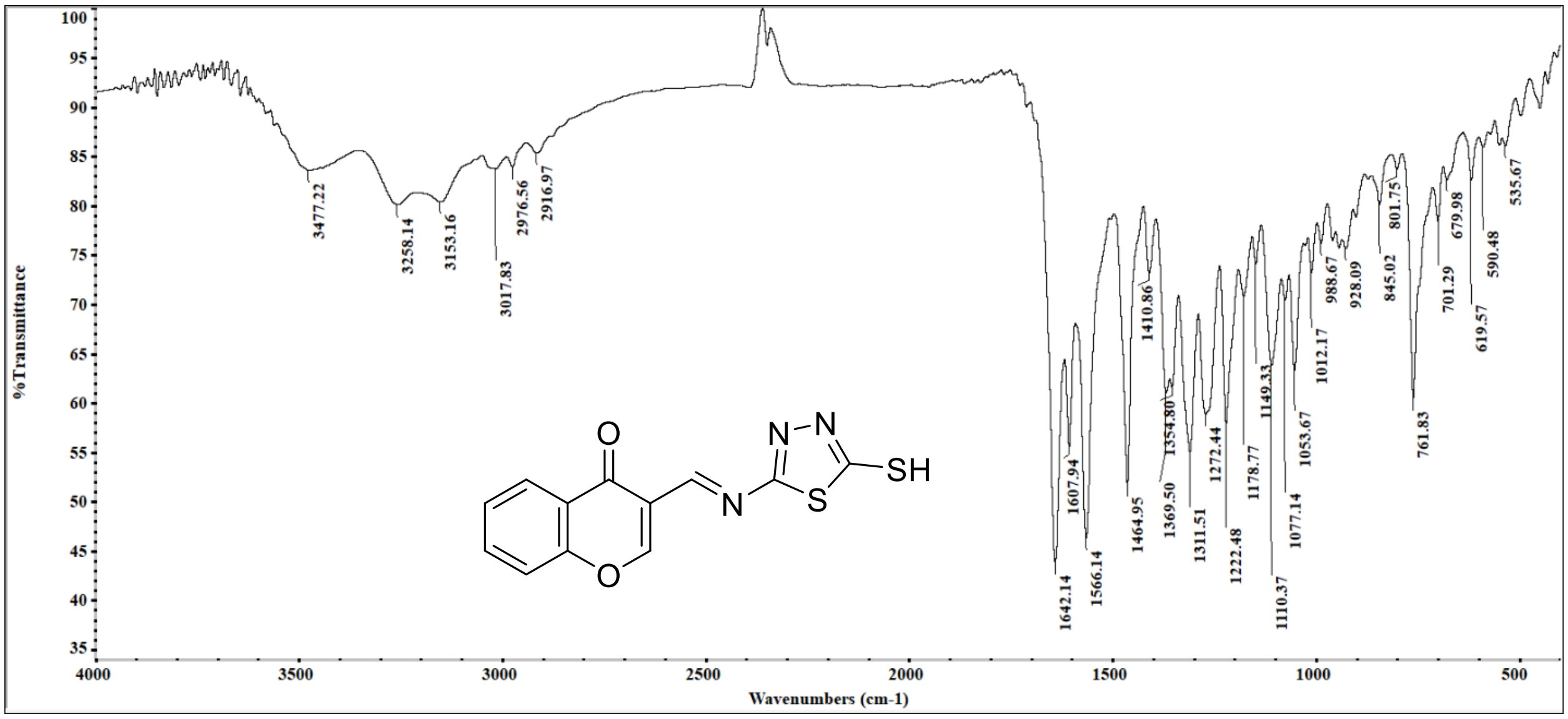

Fig.S7; FT-IR Spectrum of compound 5

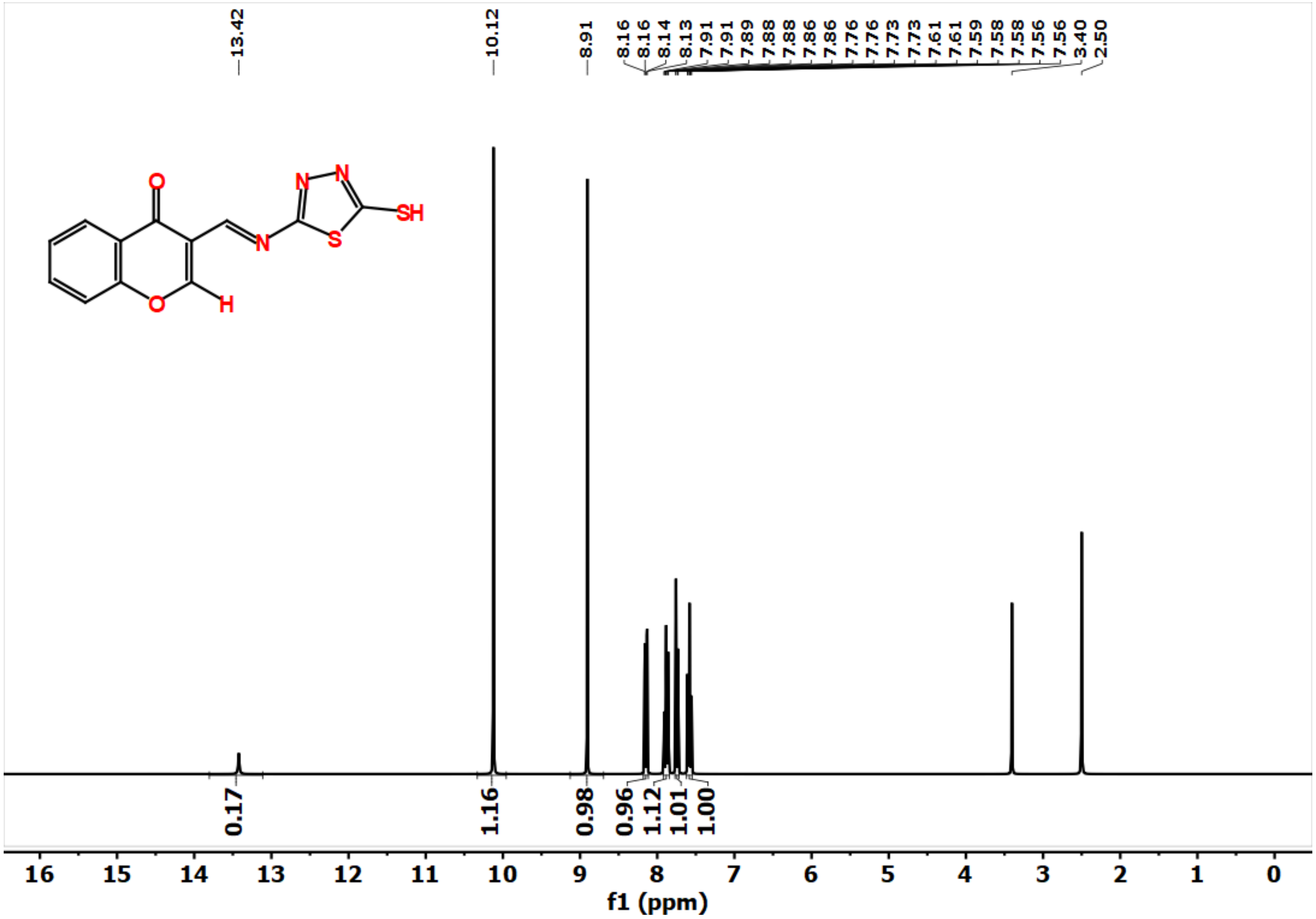

Fig. S8; ^{1}H NMR (DMSO-d_6) Spectrum of compound 5

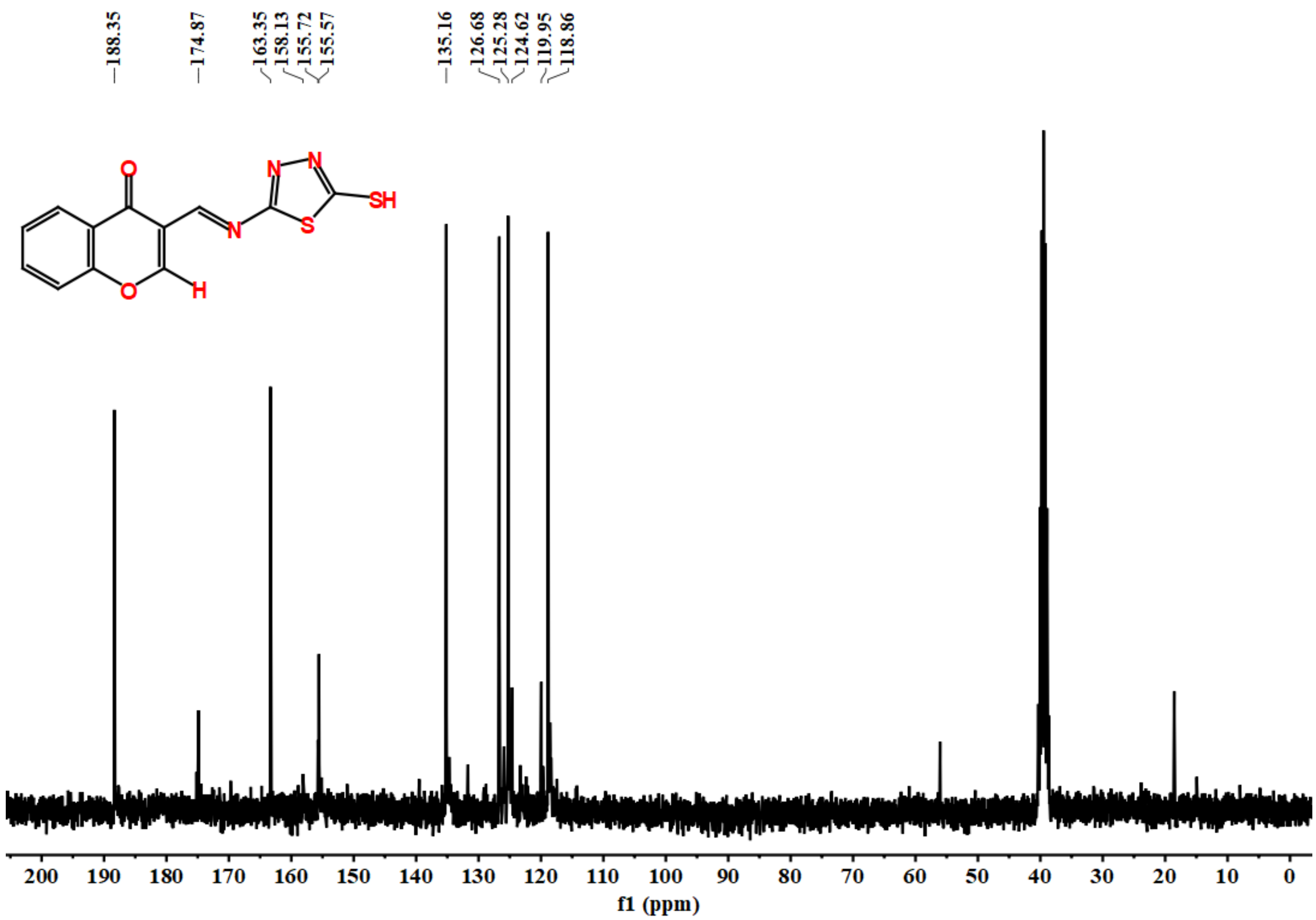

Fig. S9; ^{13}C NMR (DMSO-d_6) Spectrum of compound 5

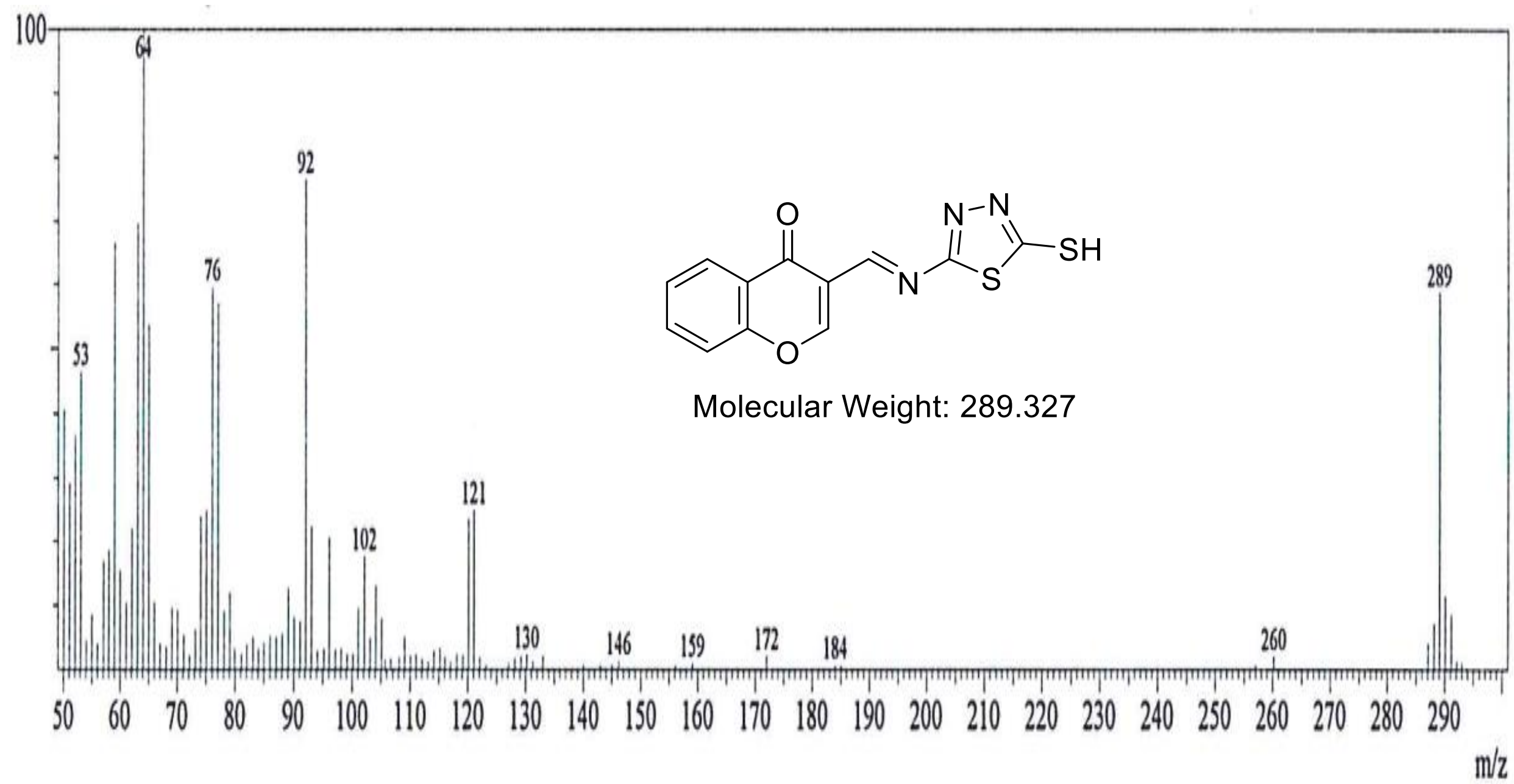

Fig. S10; Mass Spectrum of compound 5

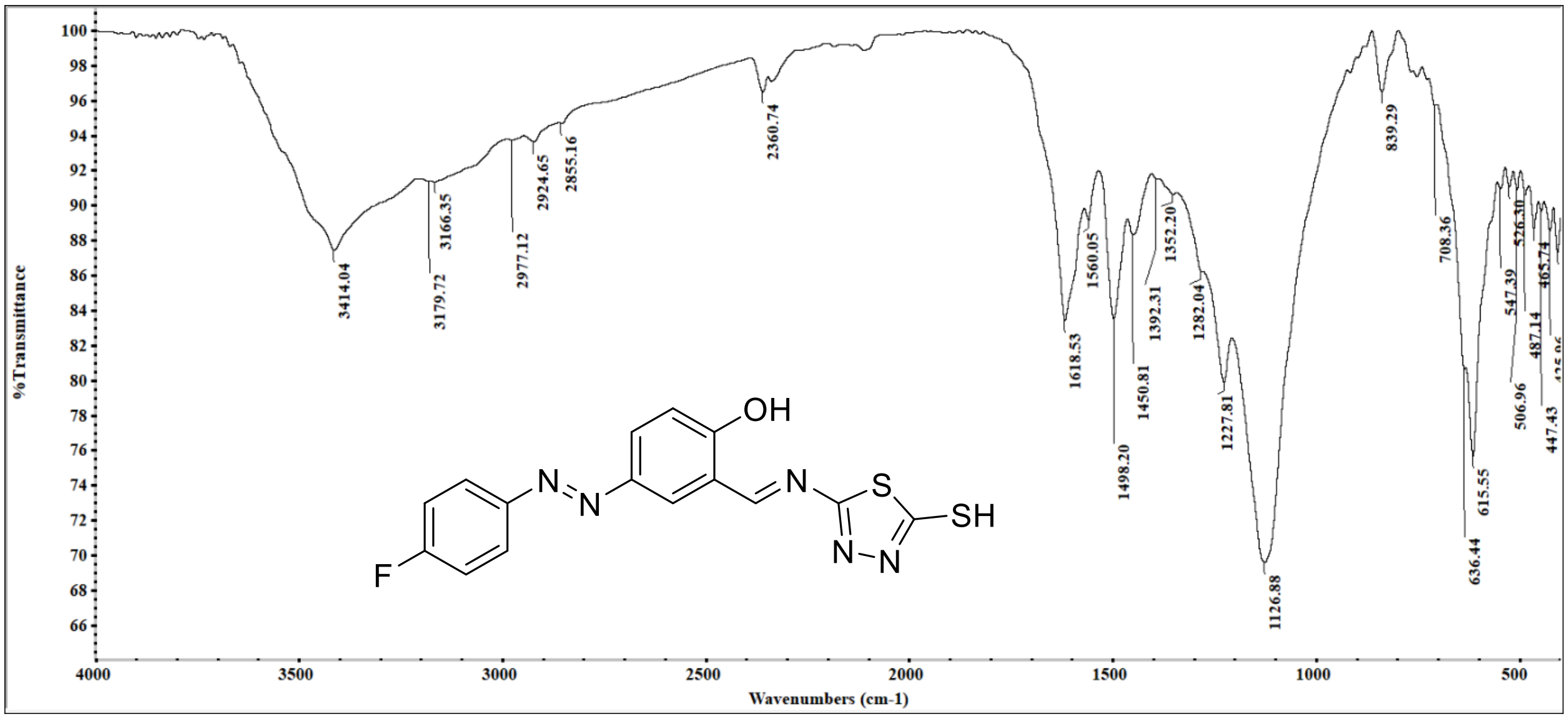

Fig.S11; FT-IR Spectrum of compound 6

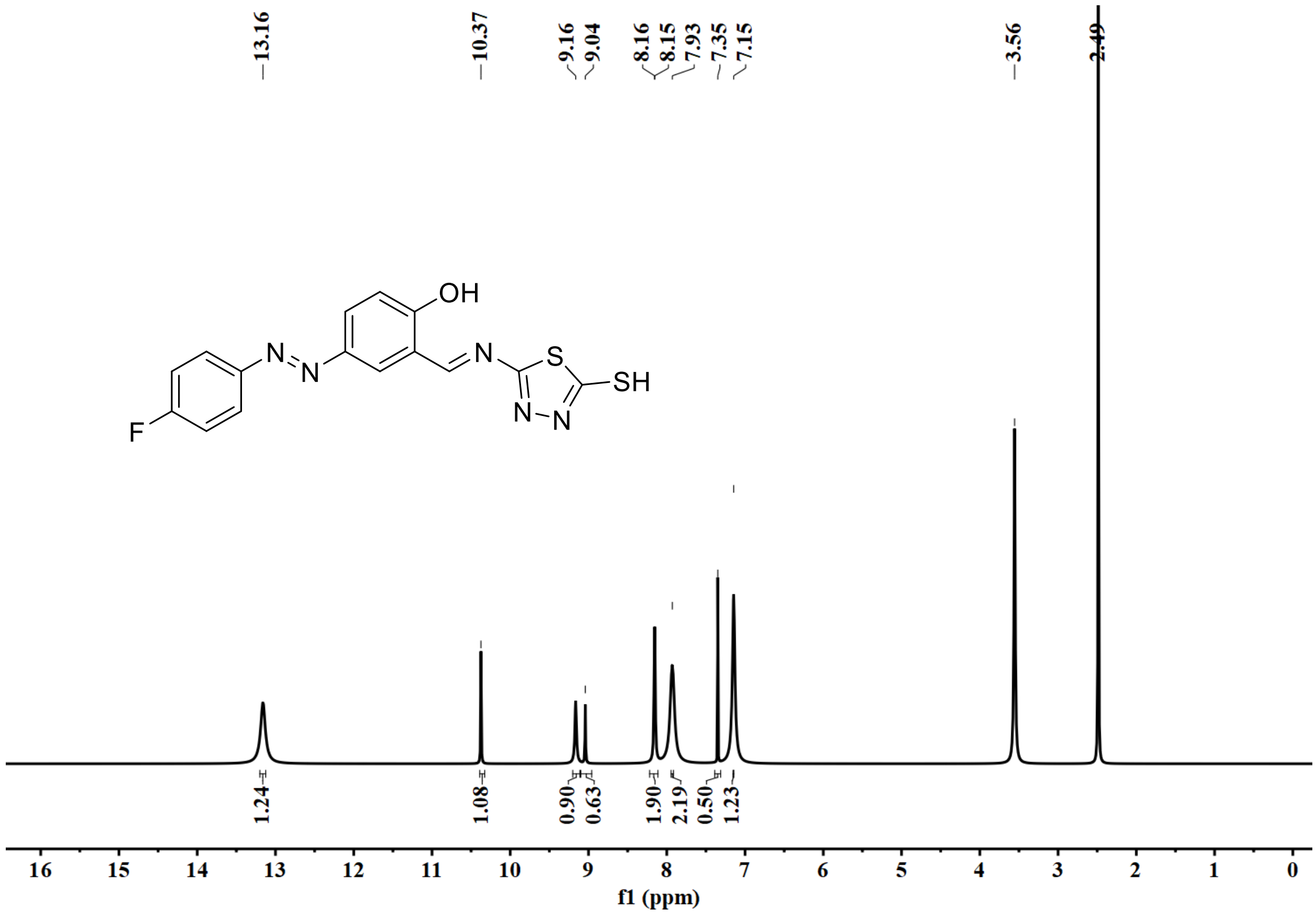

Fig. S12; ^{1}H NMR (DMSO-d_6) Spectrum of compound 6

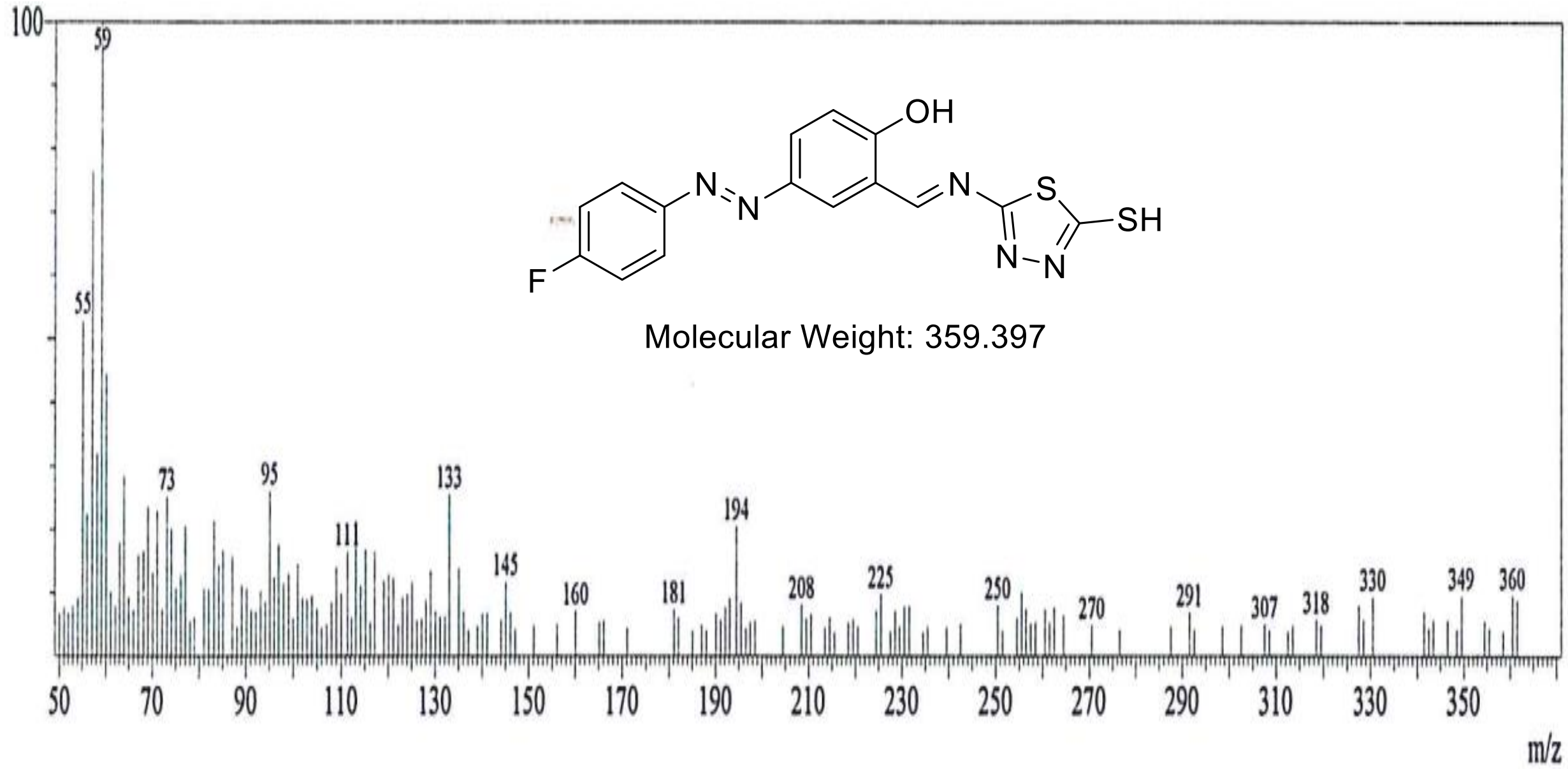

Fig. S13; Mass Spectrum of compound 6

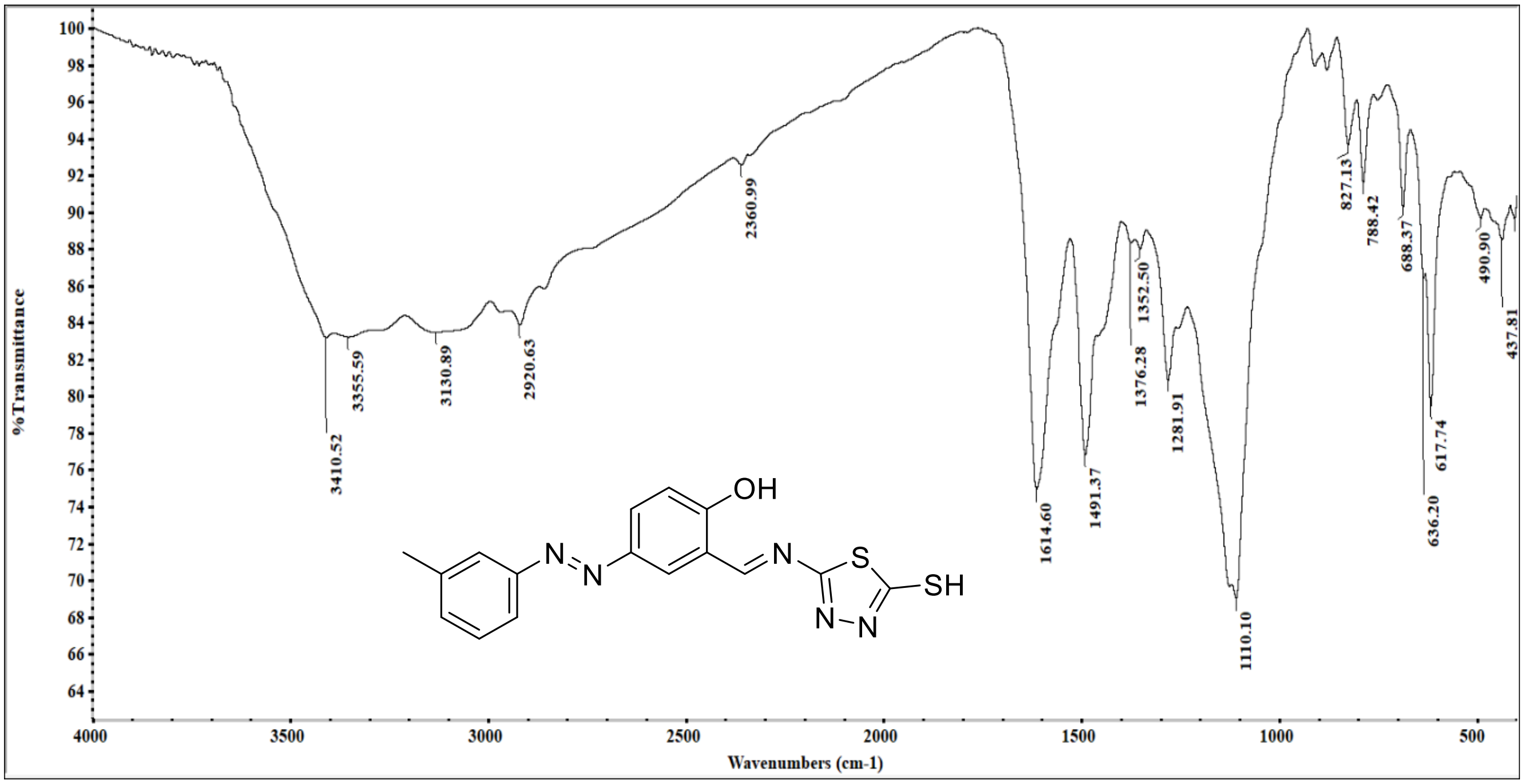

Fig.S14; FT-IR Spectrum of compound 7

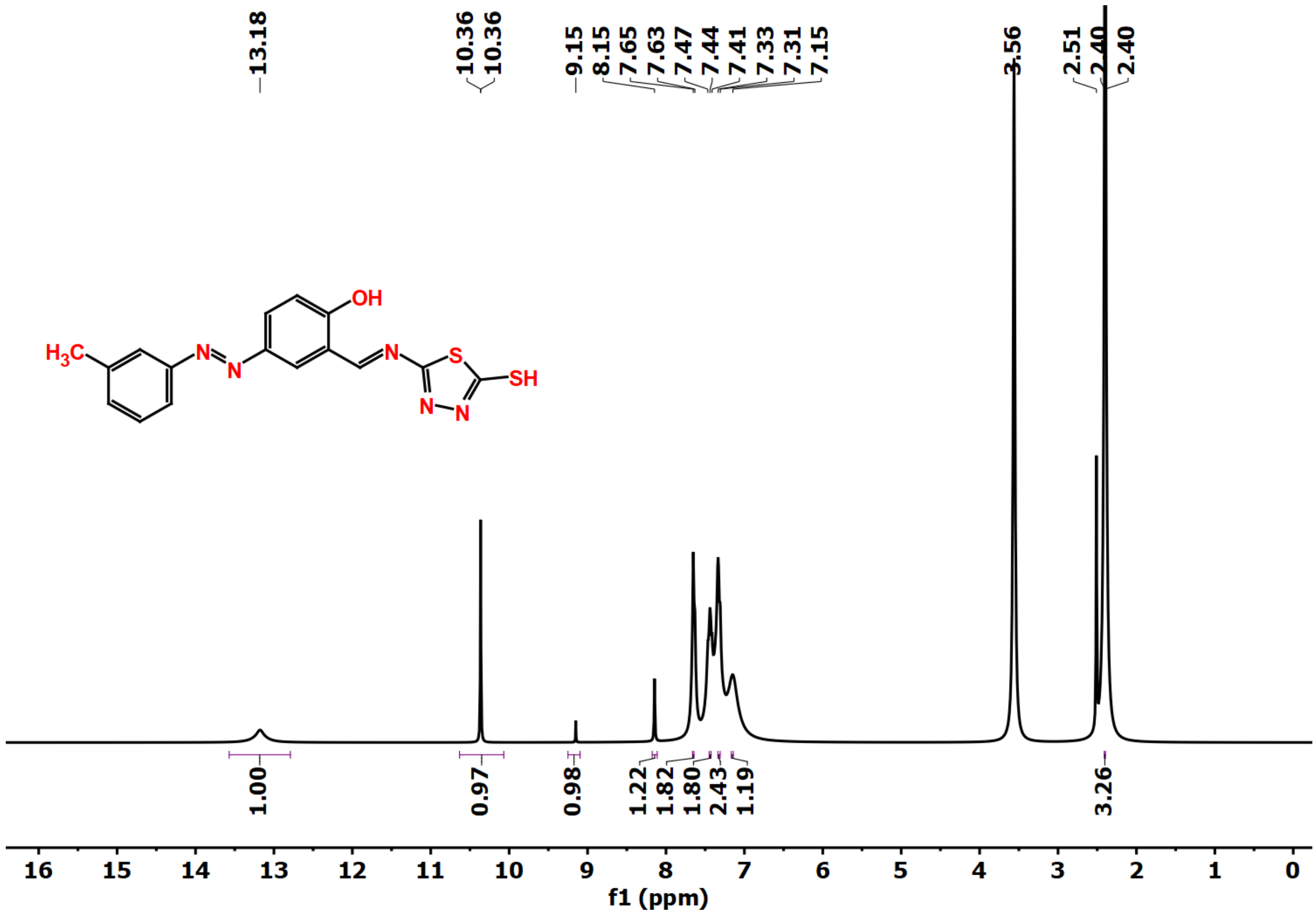

Fig. S15; ^{1}H NMR (DMSO-d_6) Spectrum of compound 7

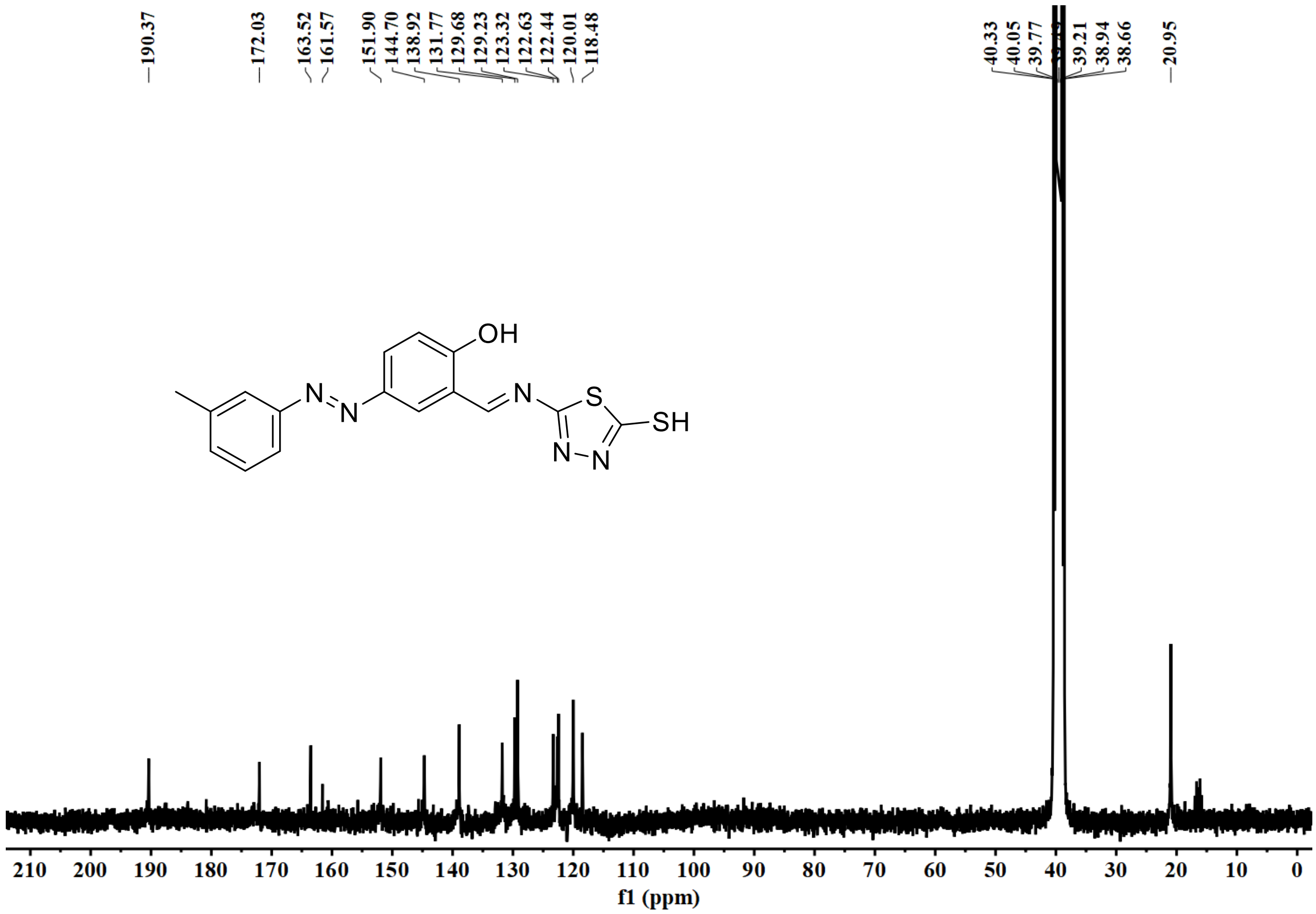

Fig. S16; ^{13}C NMR (DMSO-d_6) Spectrum of compound 7

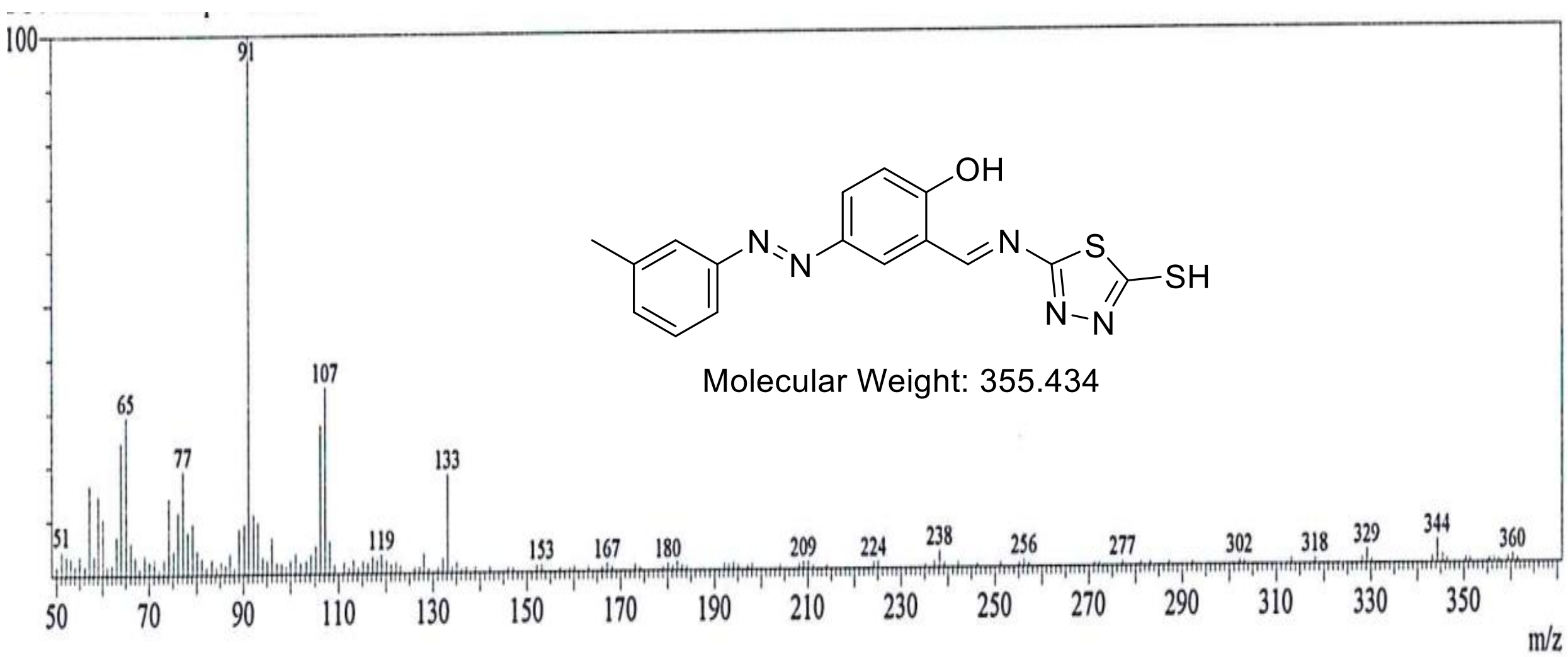

Fig. S17; Mass Spectrum of compound 7

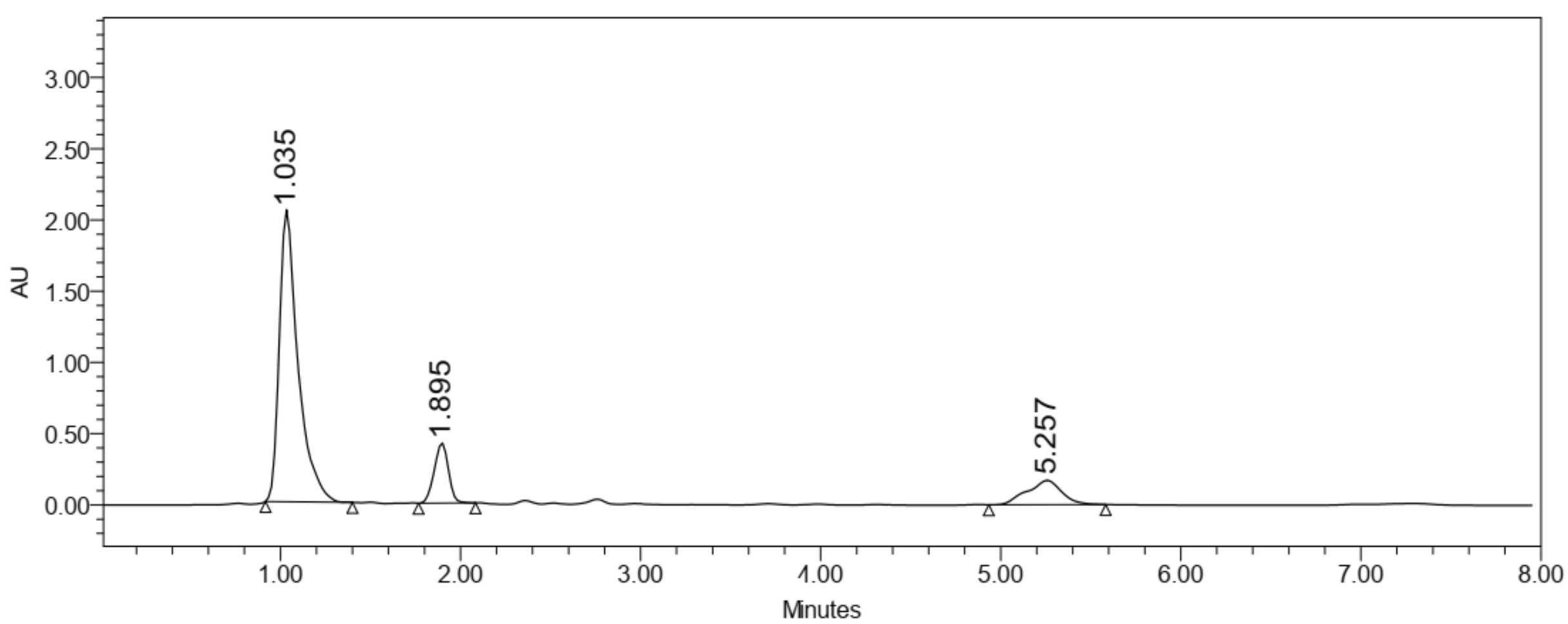

	RT	Area	% Area
1	1.035	8984225	95.17
2	1.895	229938	2.43
3	5.257	226128	2.39

Fig. S18; HPLC of compound **7**

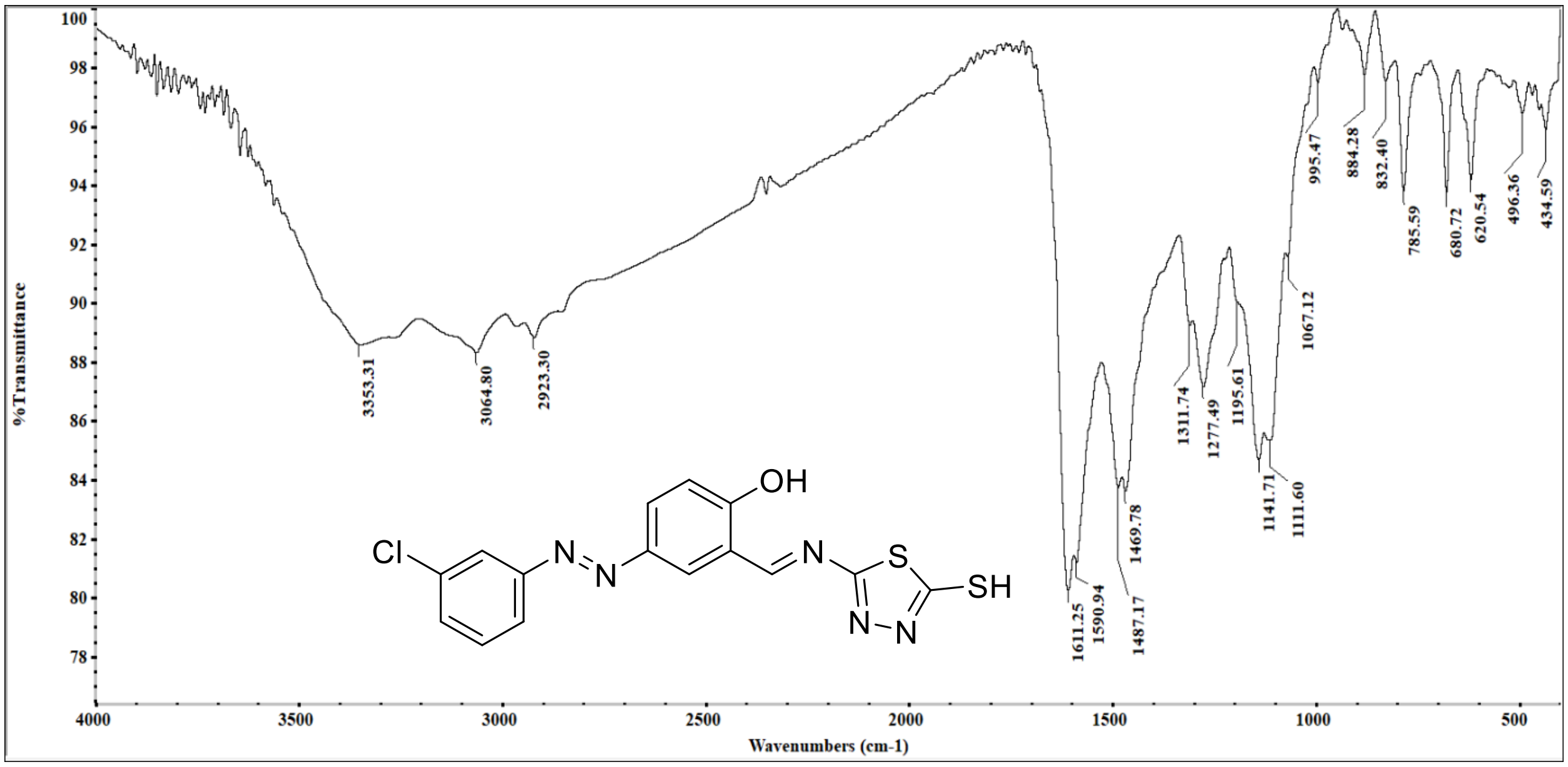

Fig.S19; FT-IR Spectrum of compound 8

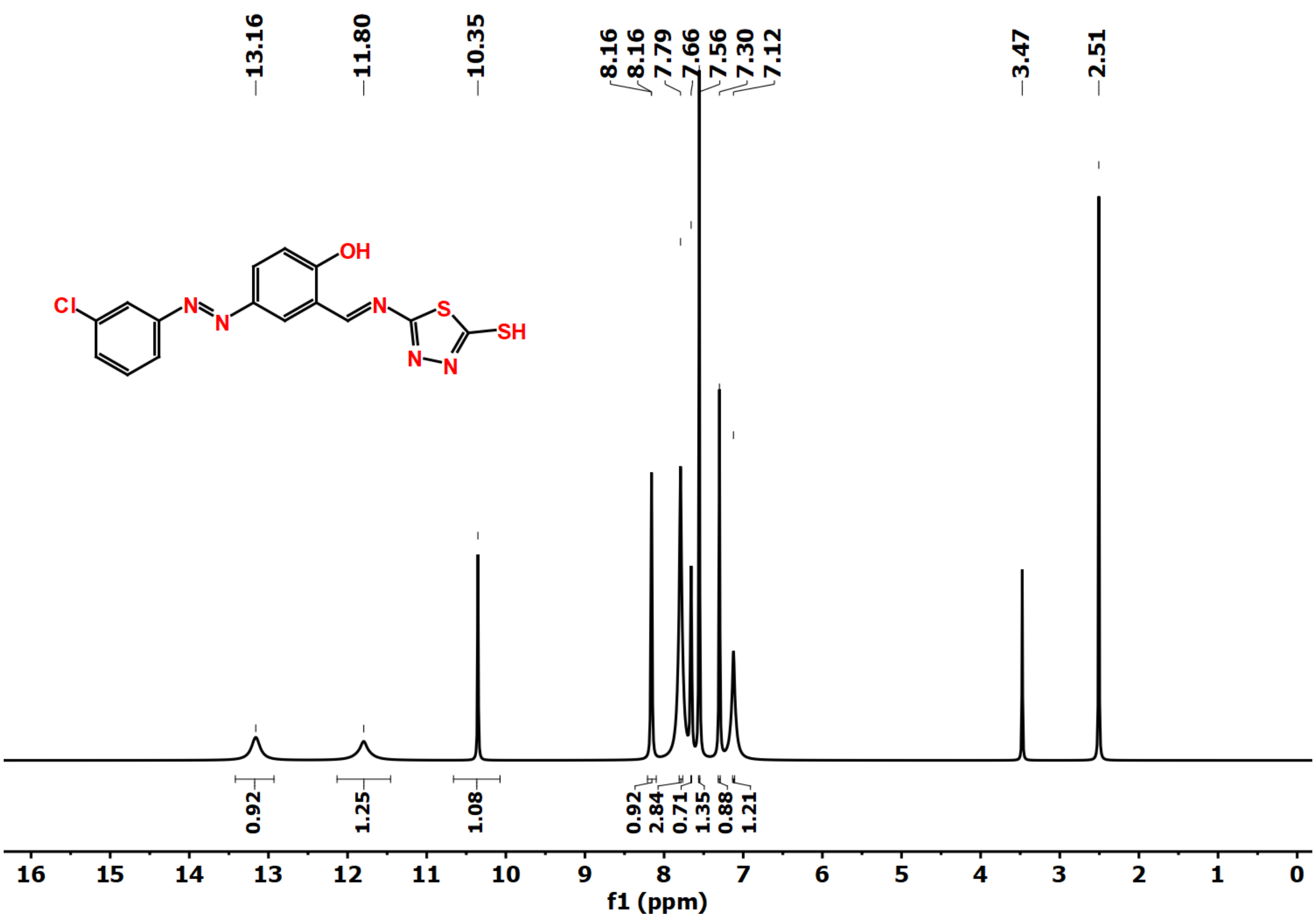

Fig. S20; ^{1}H NMR (DMSO-d_6) Spectrum of compound 8

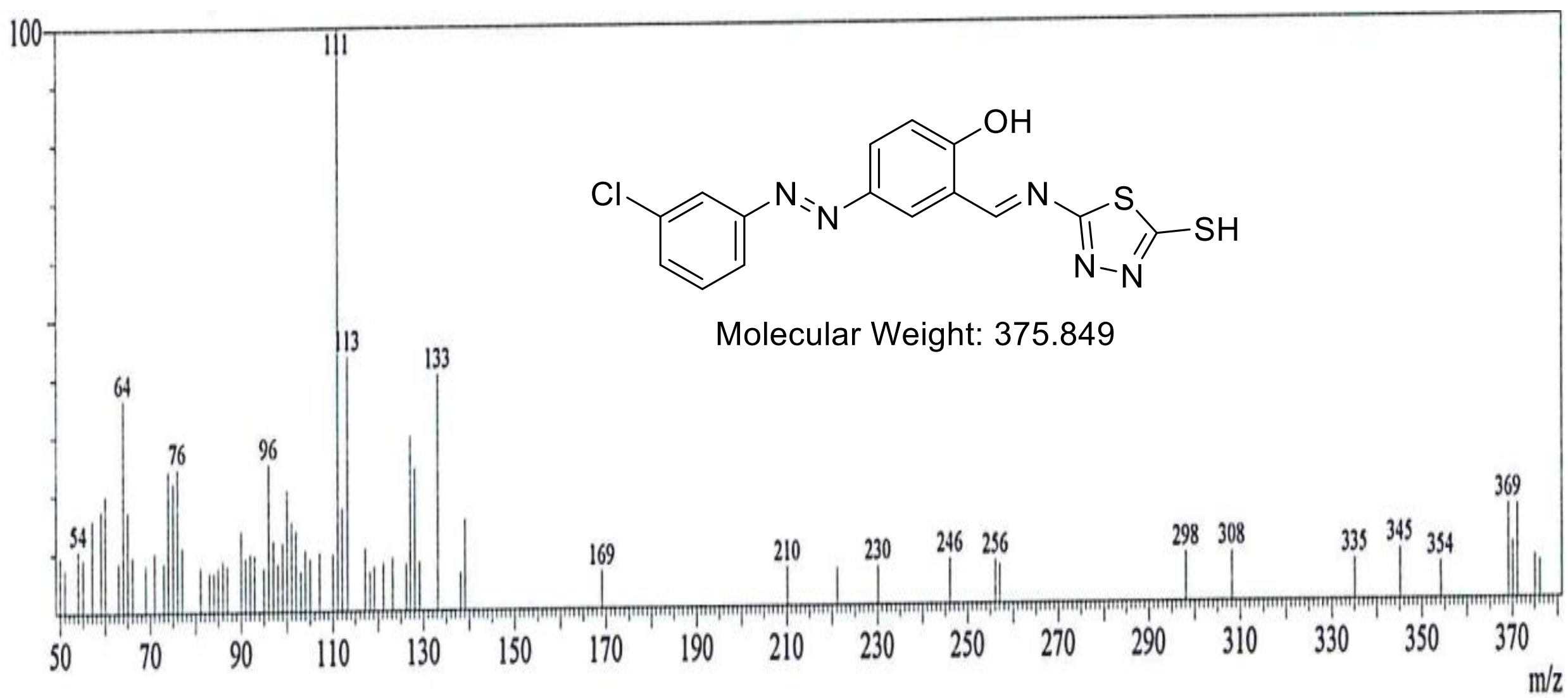

Fig. S21; Mass Spectrum of compound 8

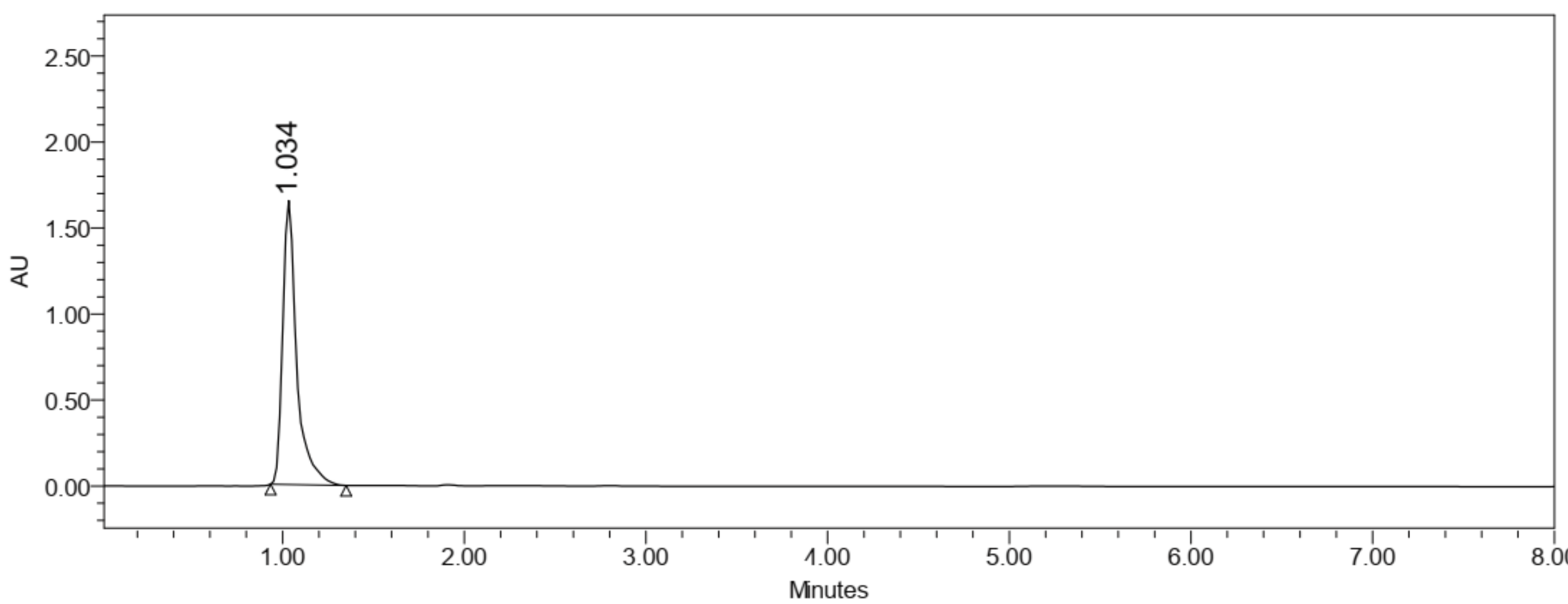

	RT	Area	% Area
1	1.034	8282617	100.00

Fig. S22; HPLC of compound **8**

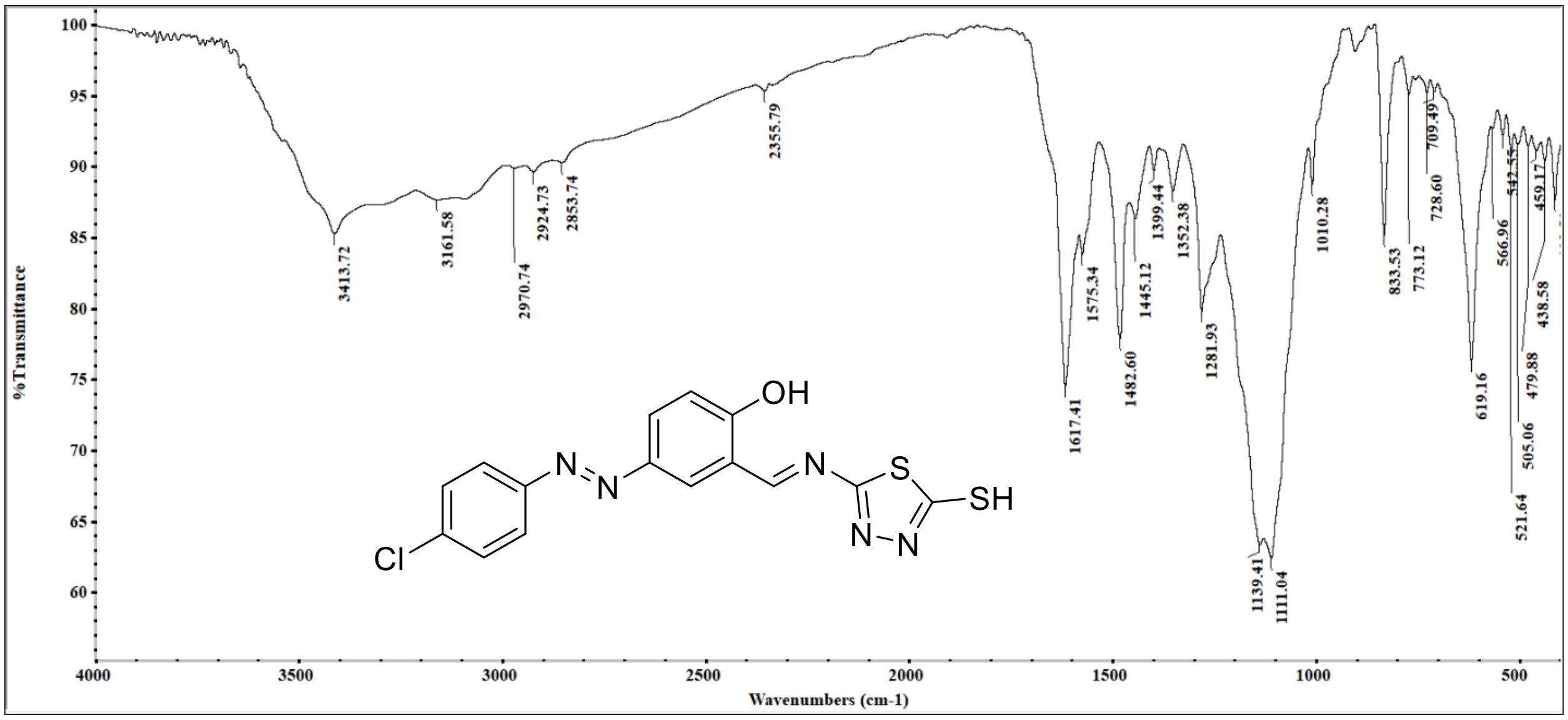

Fig.S23; FT-IR Spectrum of compound 9

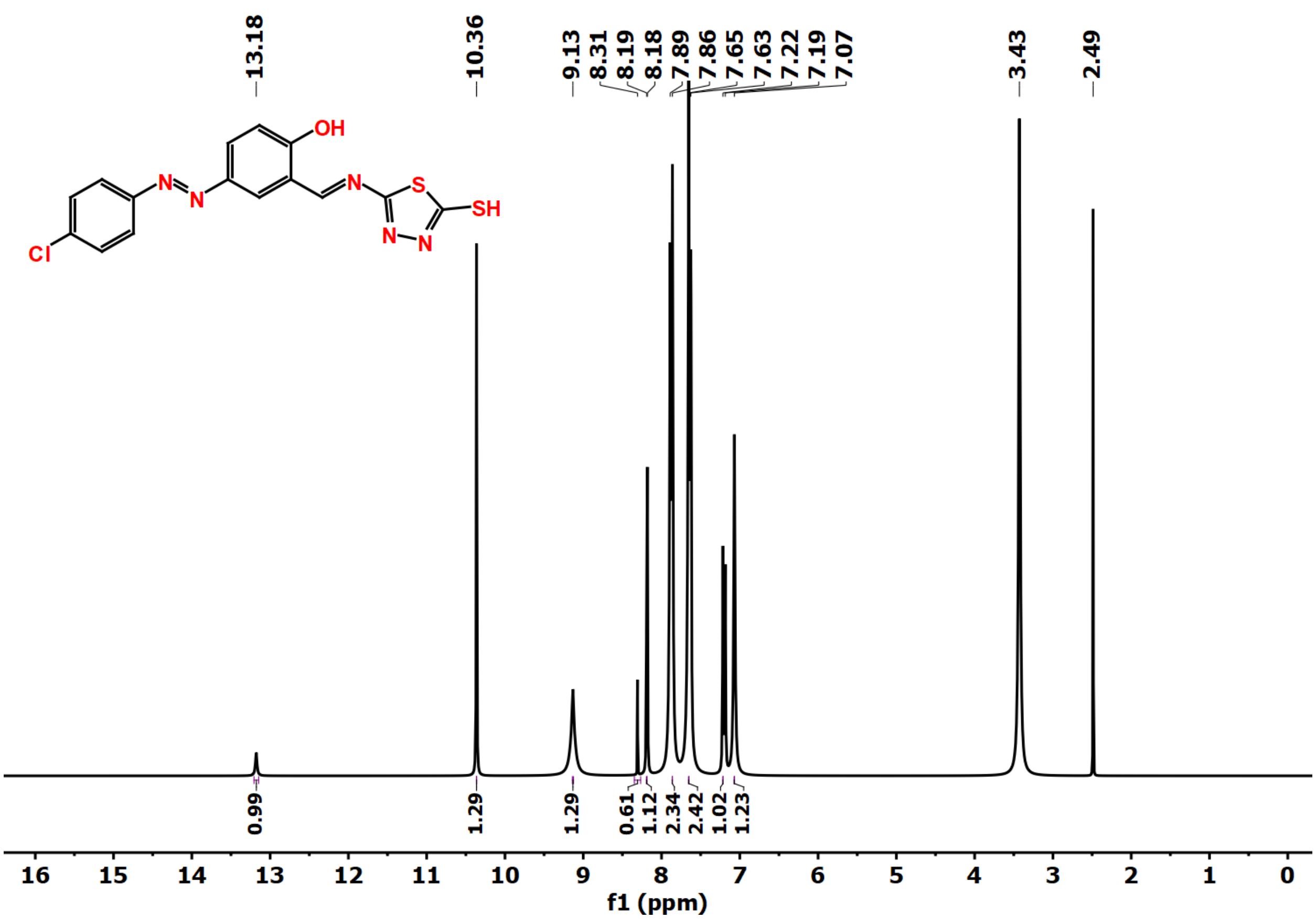

Fig. S24; ^{1}H NMR (DMSO-d_6) Spectrum of compound 9

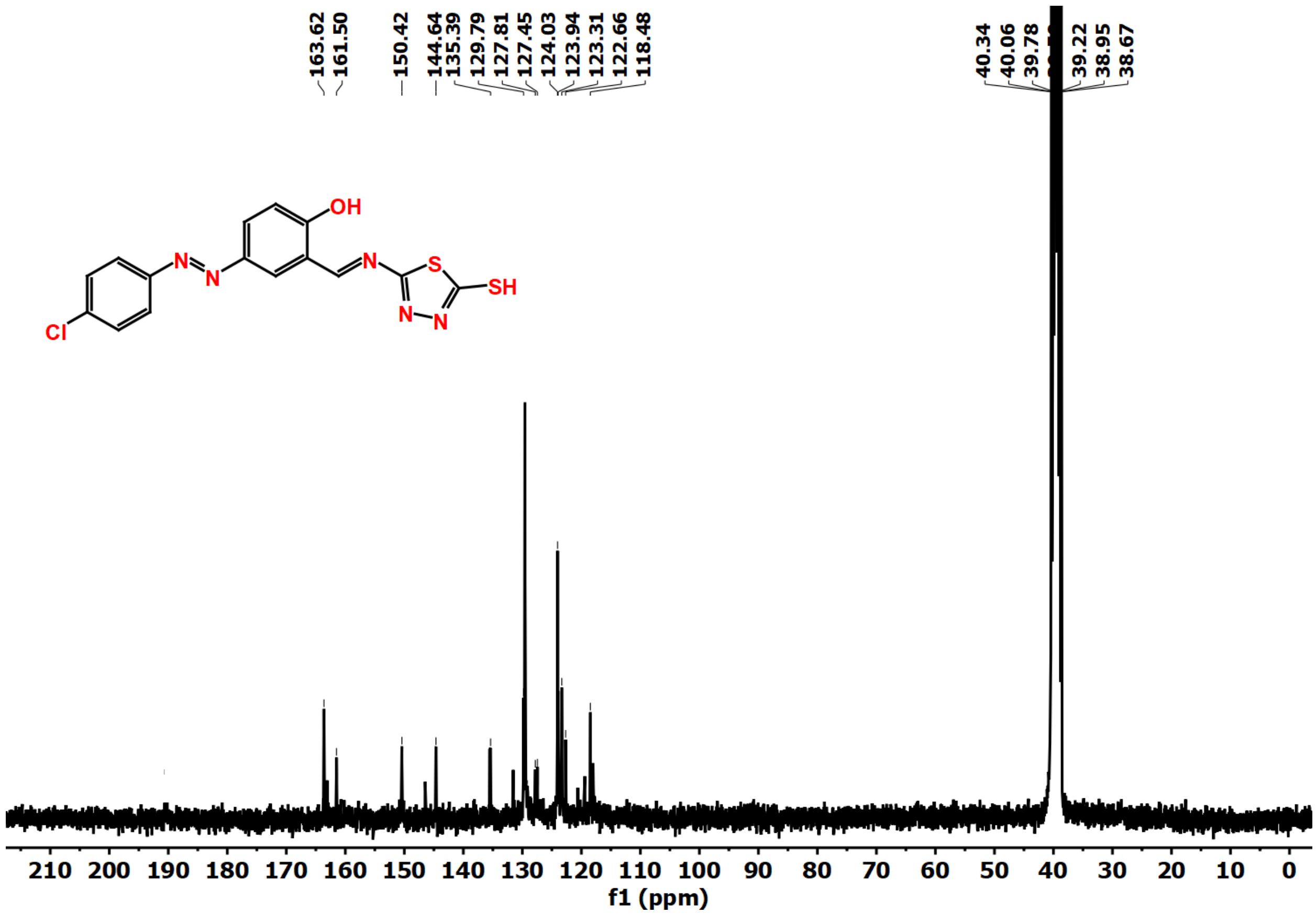

Fig. S25; ^{13}C NMR (DMSO-d_6) Spectrum of compound 9

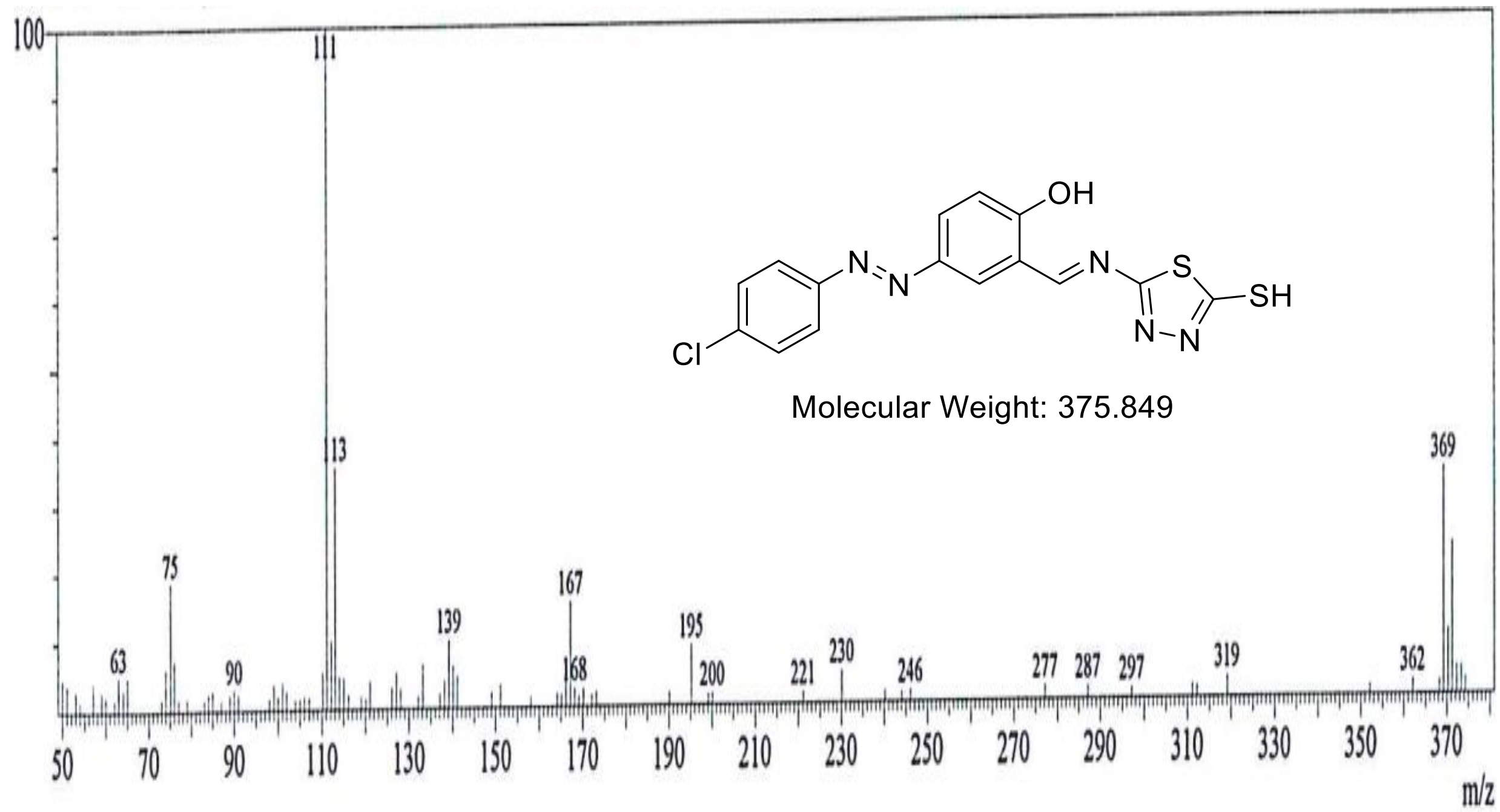

Fig. S26; Mass Spectrum of compound 9

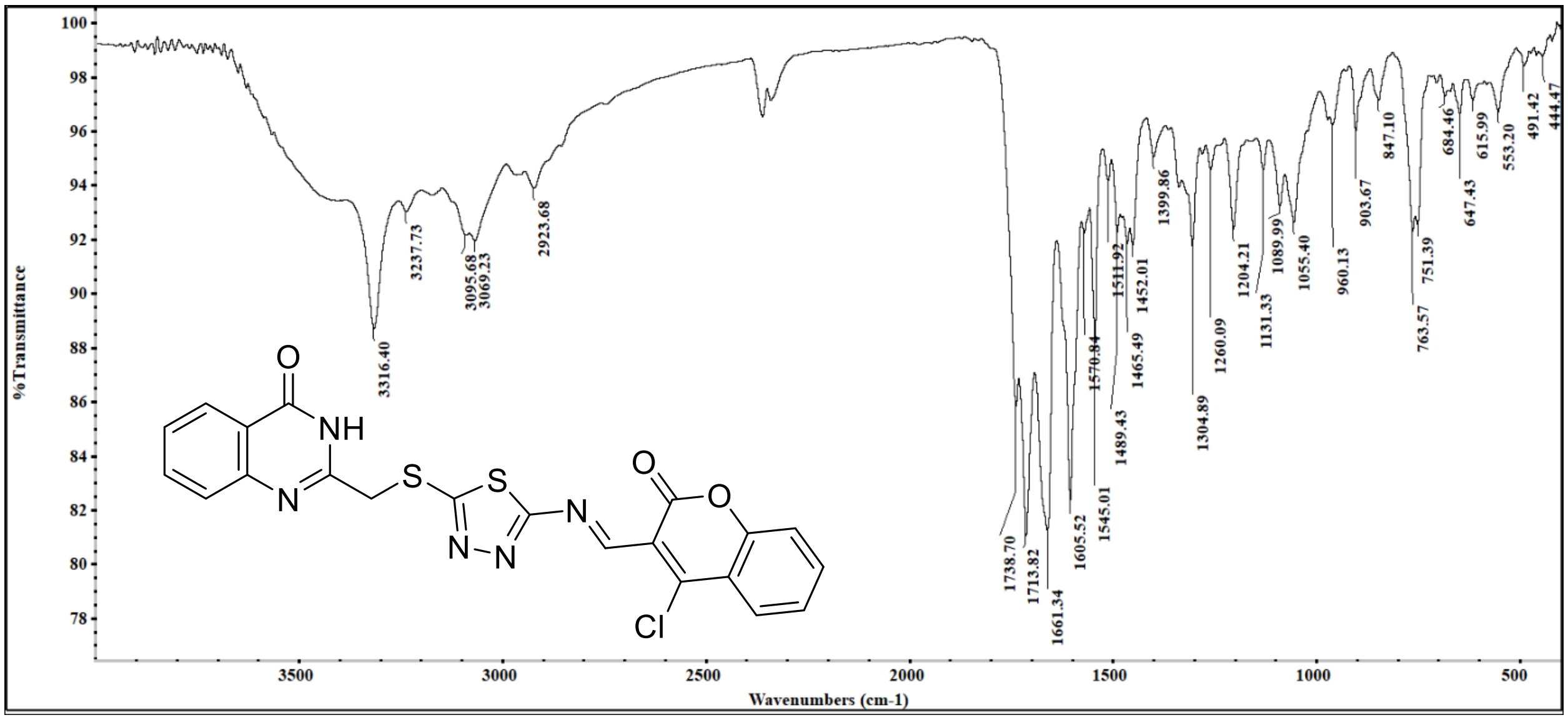

Fig.S27; FT-IR Spectrum of compound 11

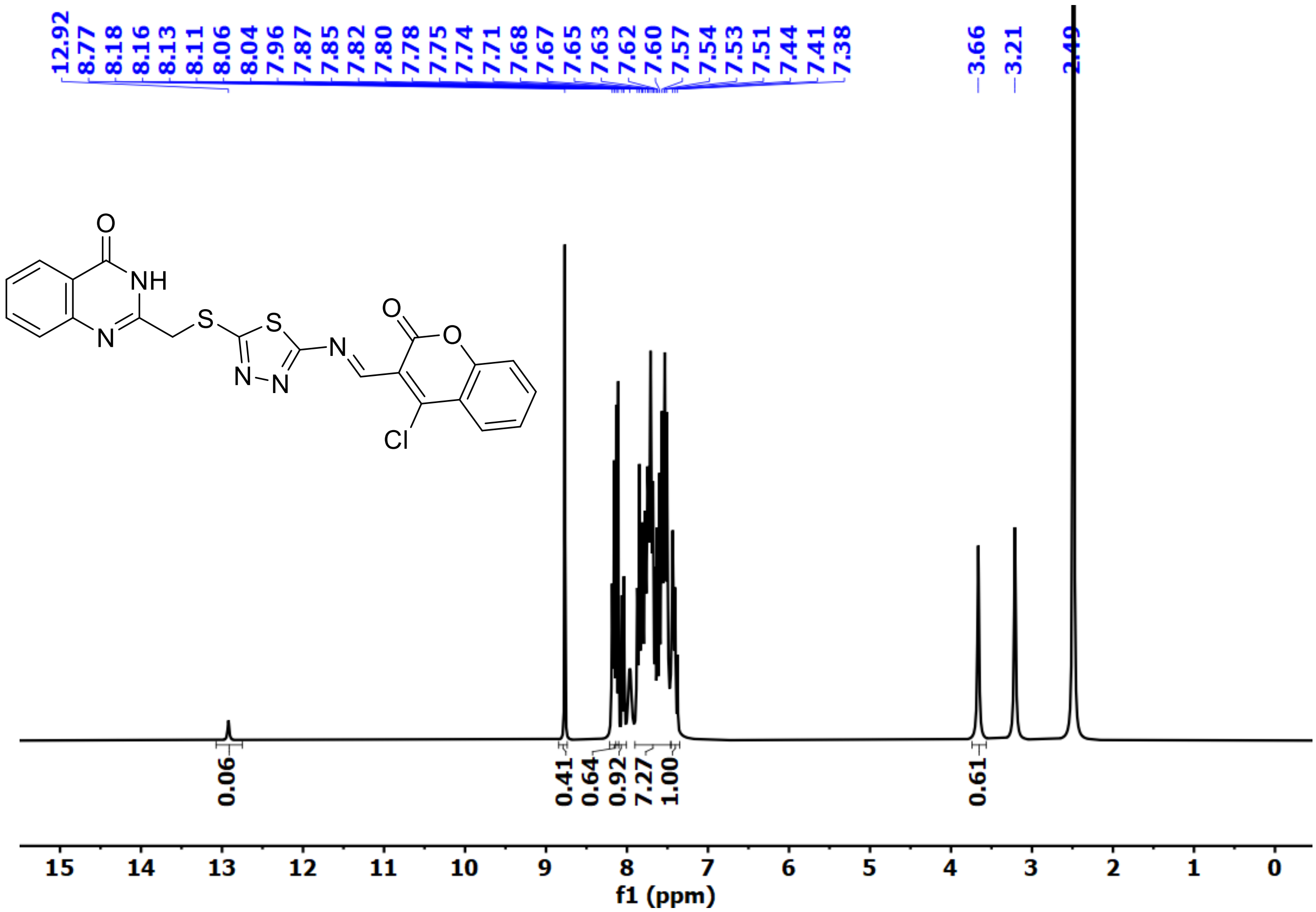

Fig. S28; ^{1}H NMR (DMSO-d_6) Spectrum of compound 11

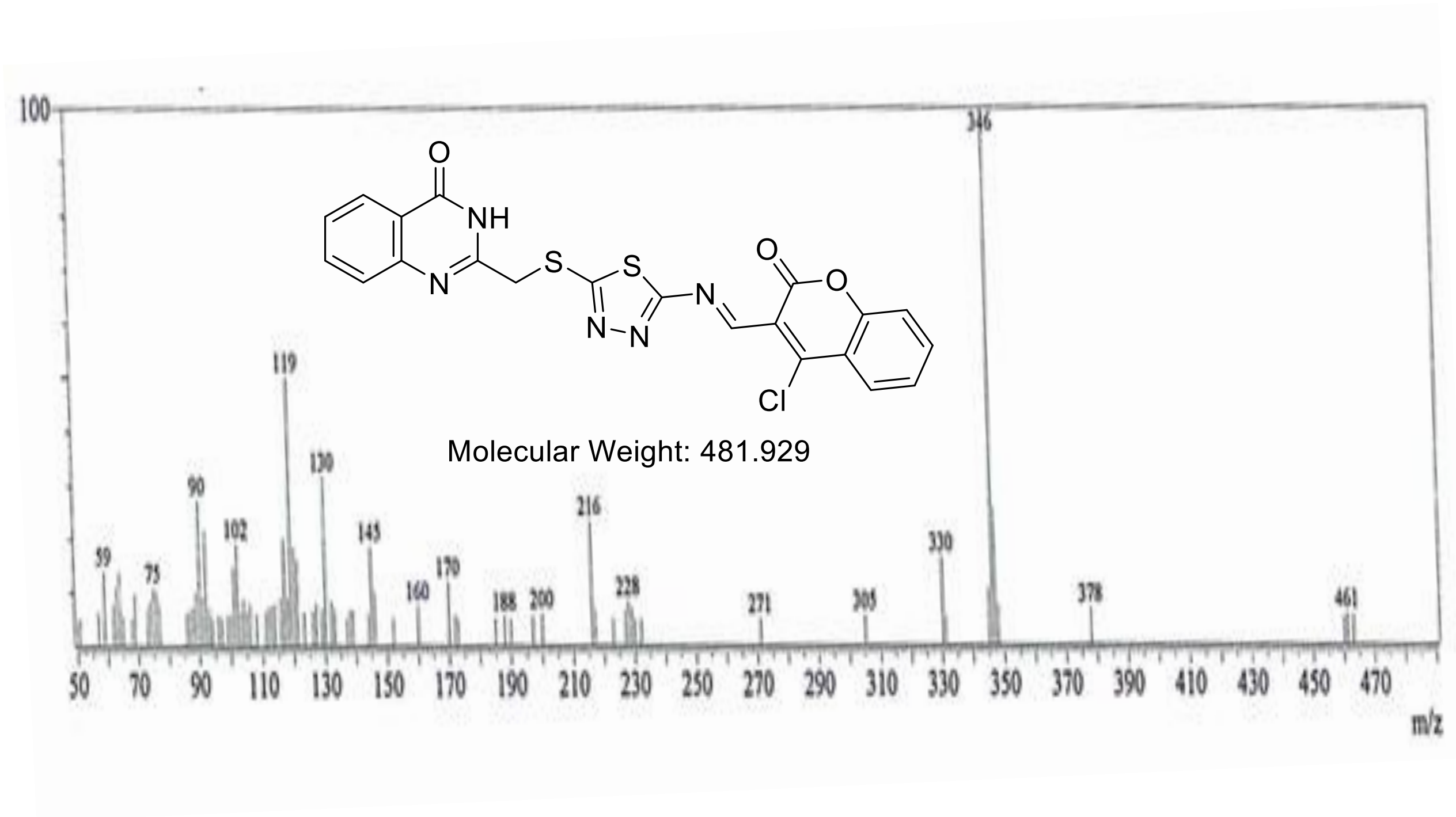

Fig. S29; Mass Spectrum of compound 11

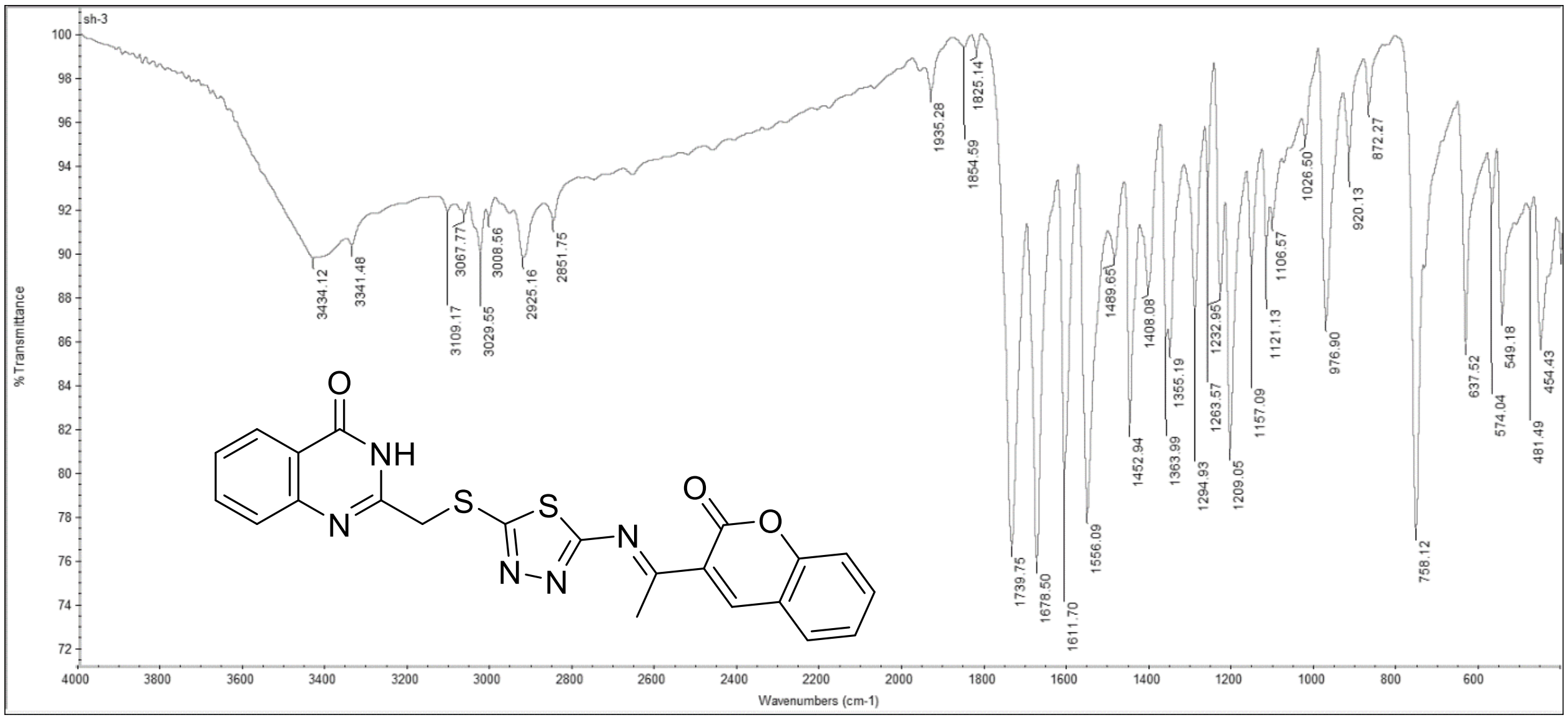

Fig.S30; FT-IR Spectrum of compound 12

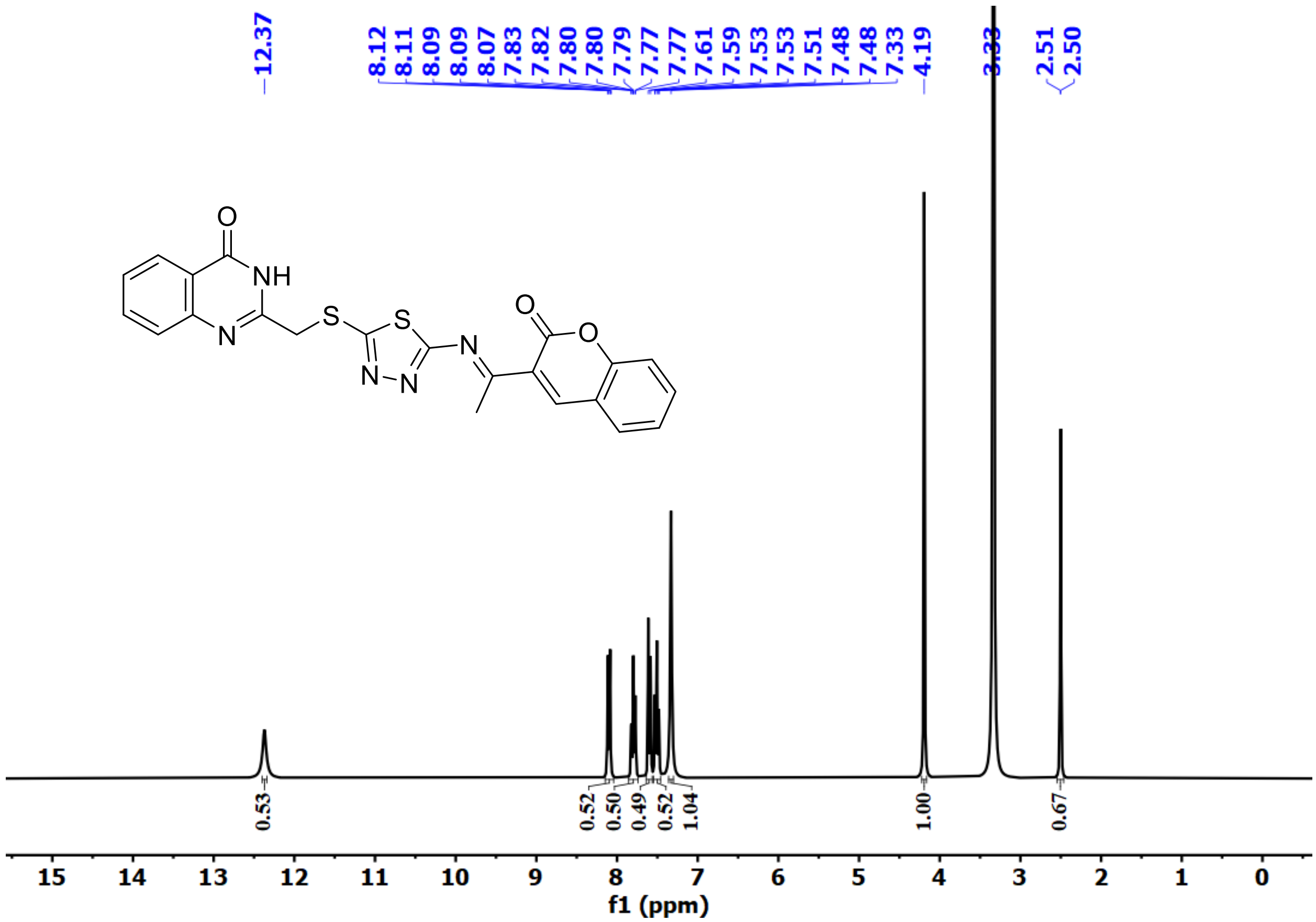

Fig. S31; ^{1}H NMR (DMSO-d_6) Spectrum of compound 12

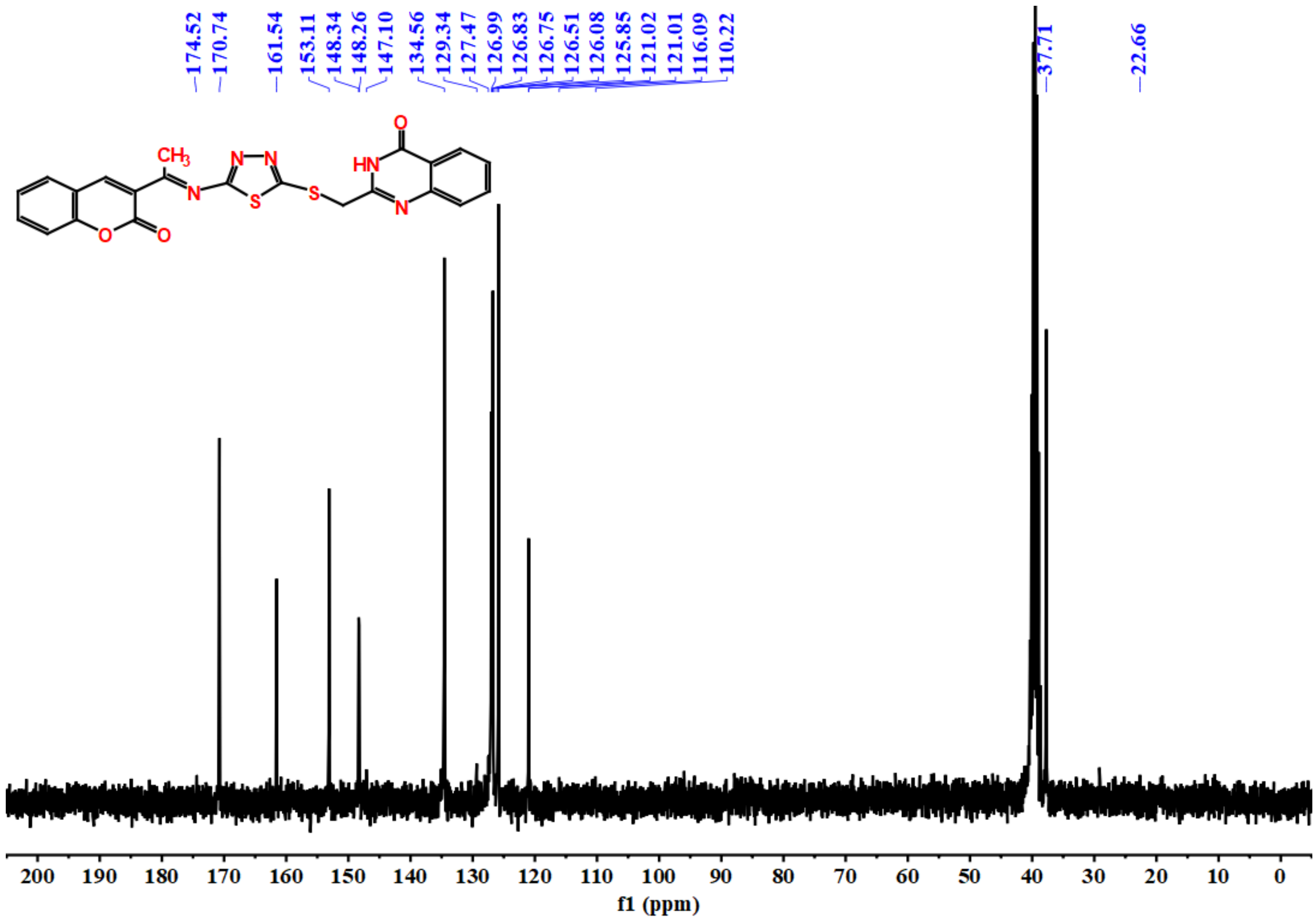

Fig. S32; ^{13}C NMR (DMSO-d_6) Spectrum of compound 12

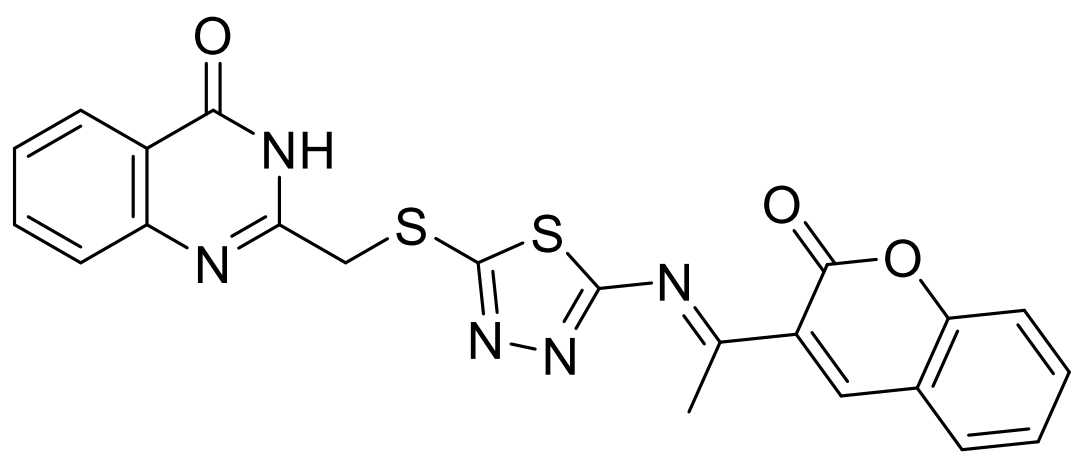
Molecular Weight: 461.514

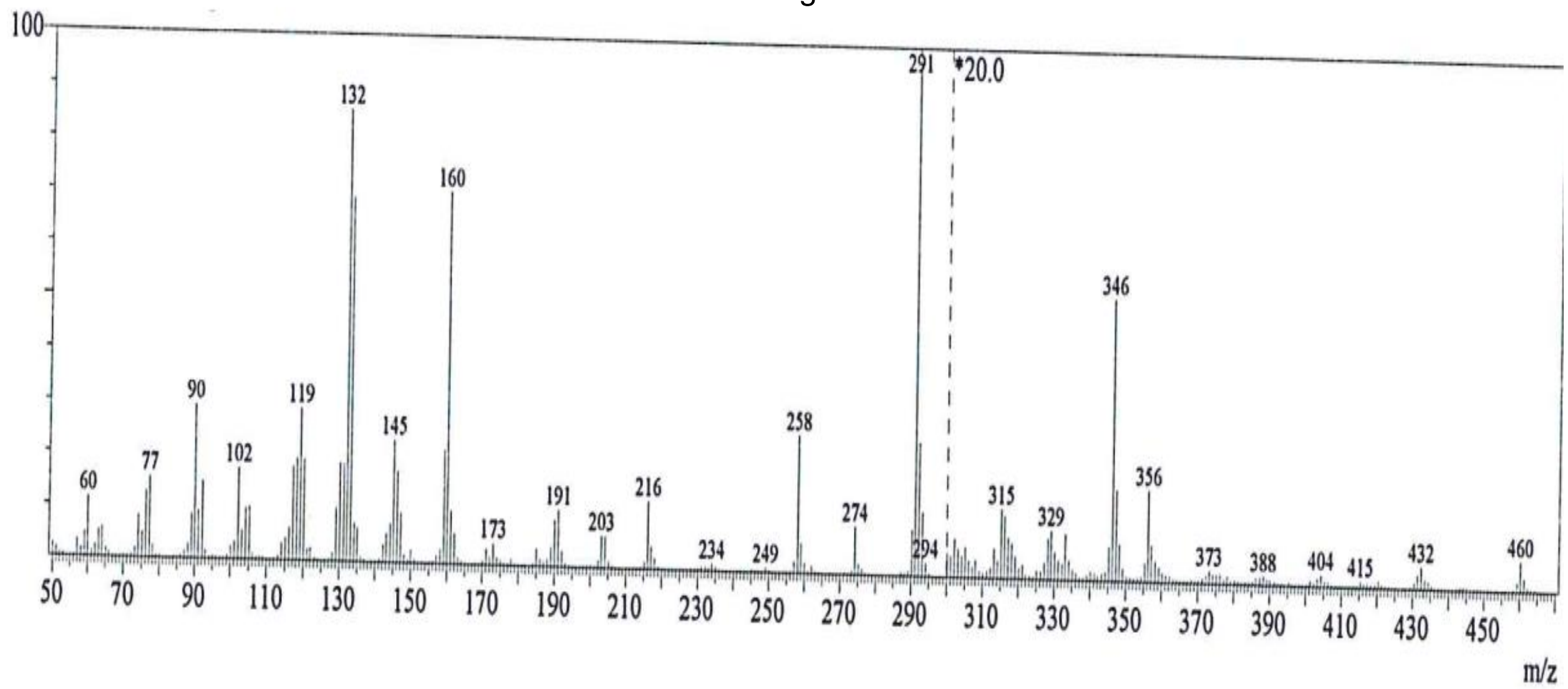

Fig. S33; Mass Spectrum of compound 12

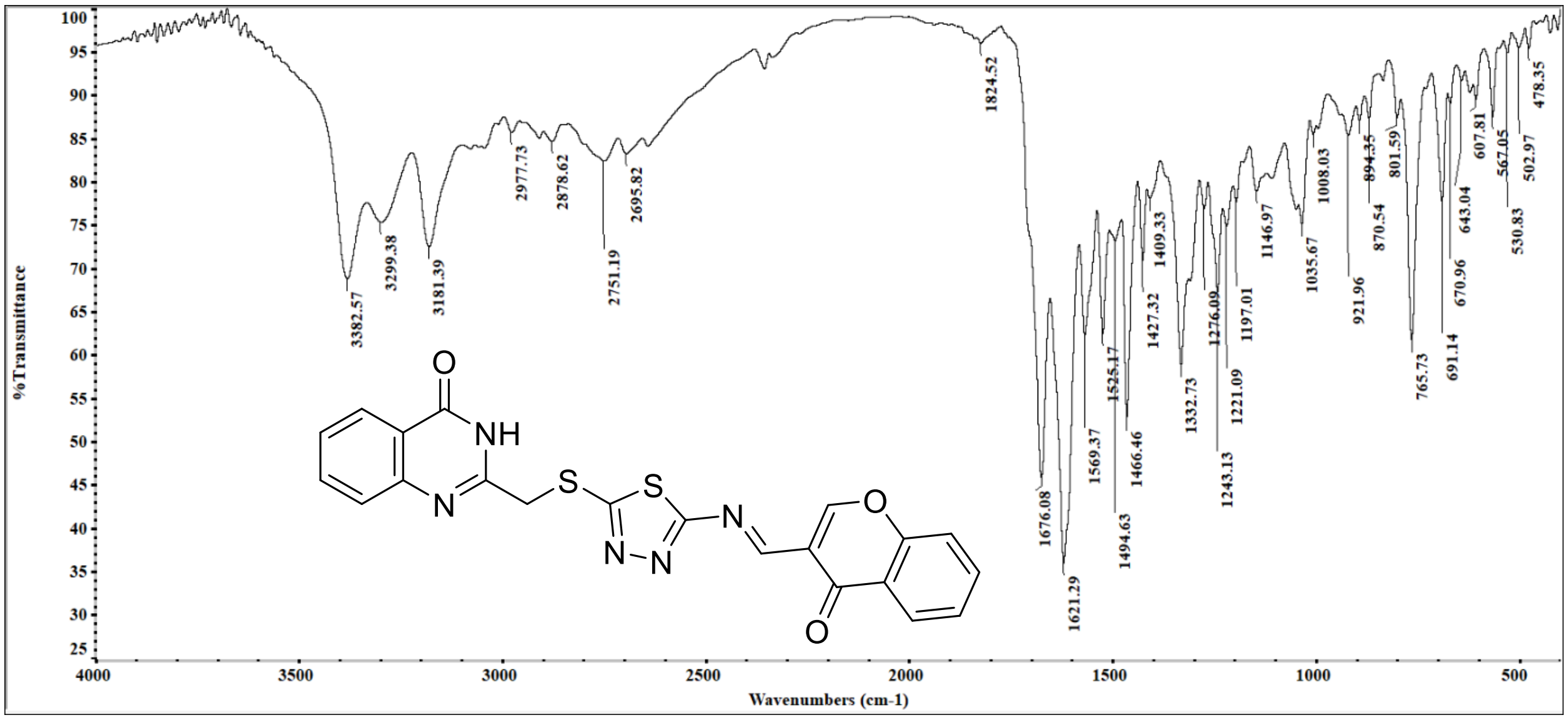

Fig.S34; FT-IR Spectrum of compound 13

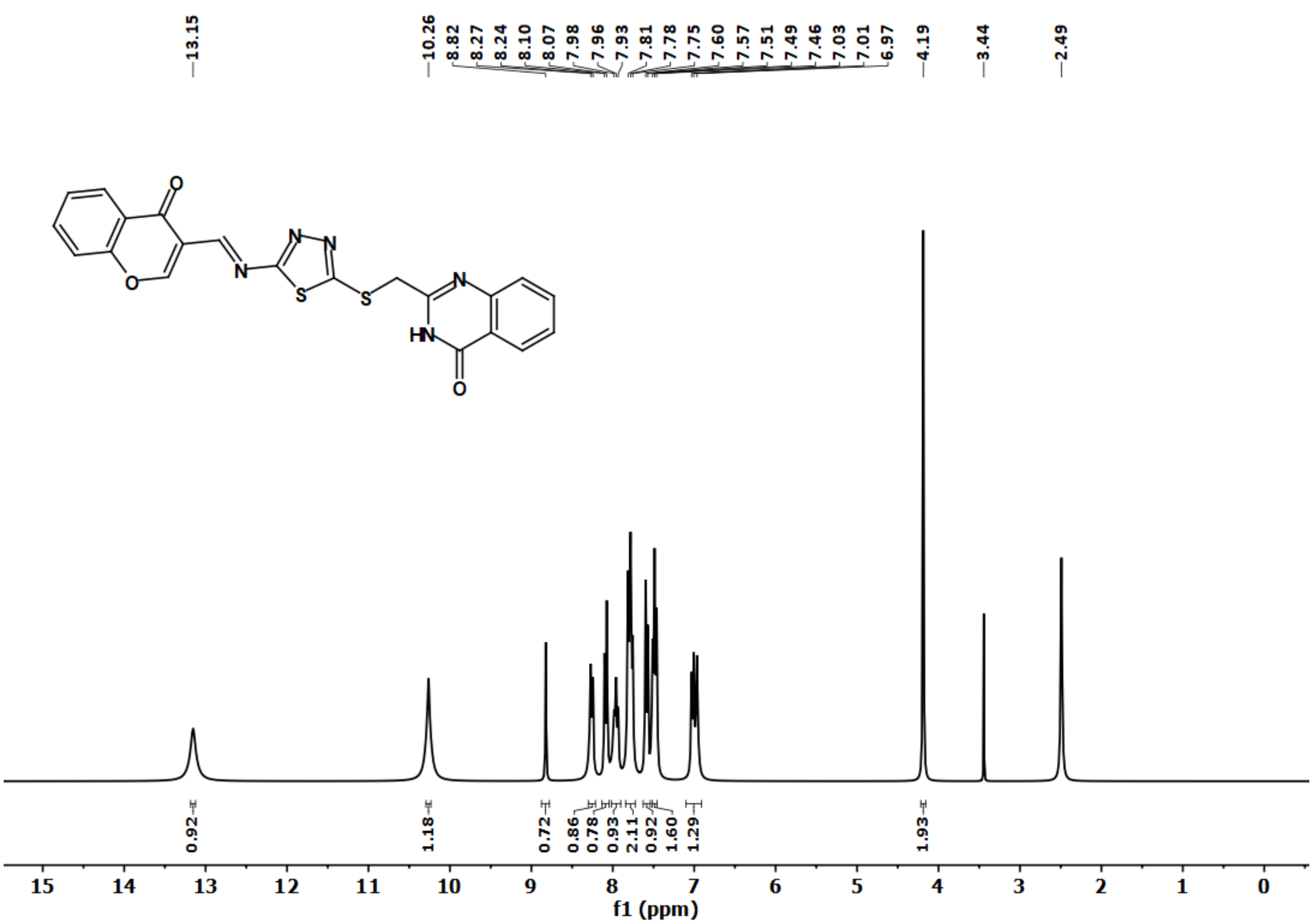

Fig. S35; ^{1}H NMR (DMSO-d$_6$) Spectrum of compound 13

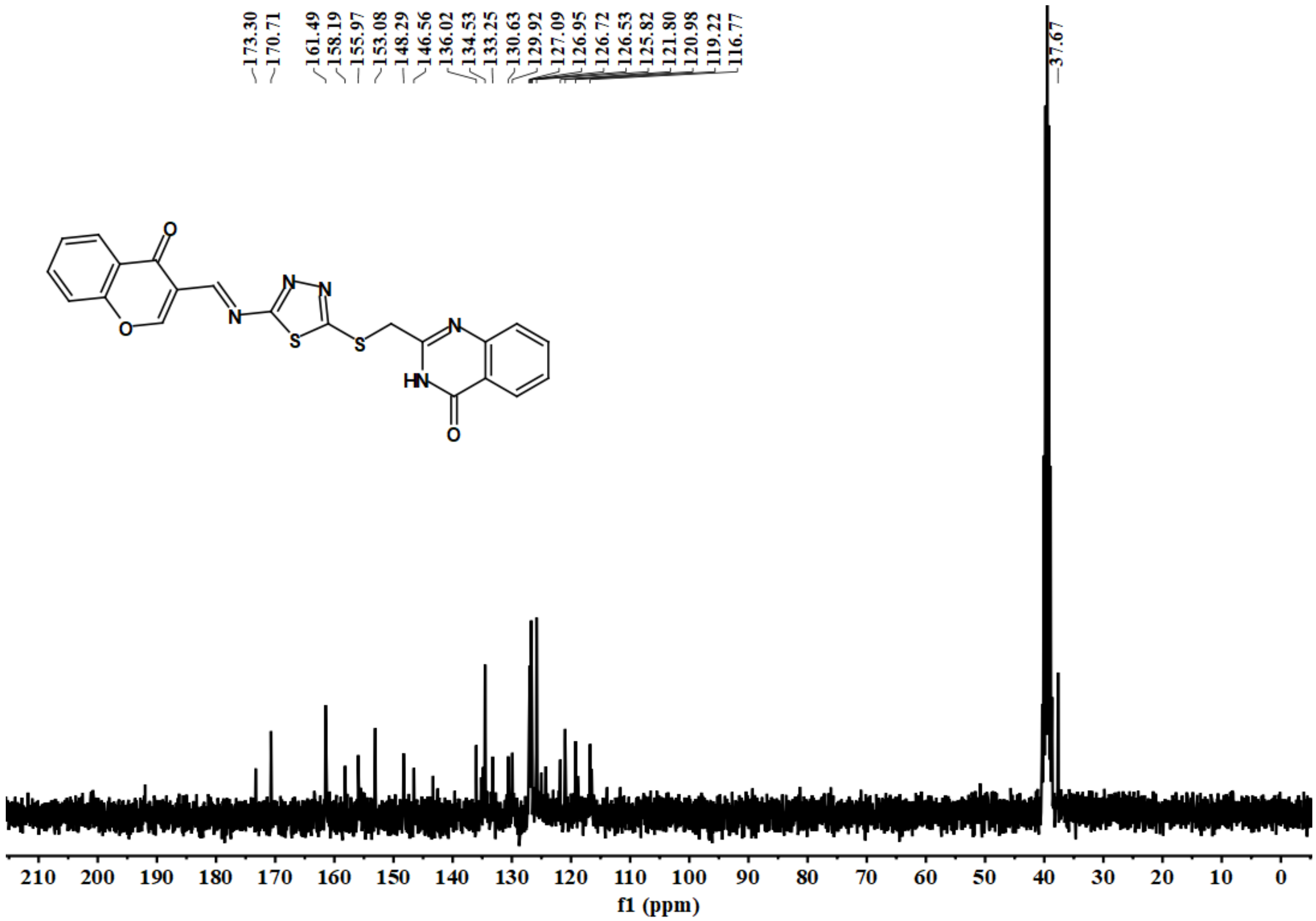

Fig. S36; ^{13}C NMR (DMSO-d_6) Spectrum of compound 13

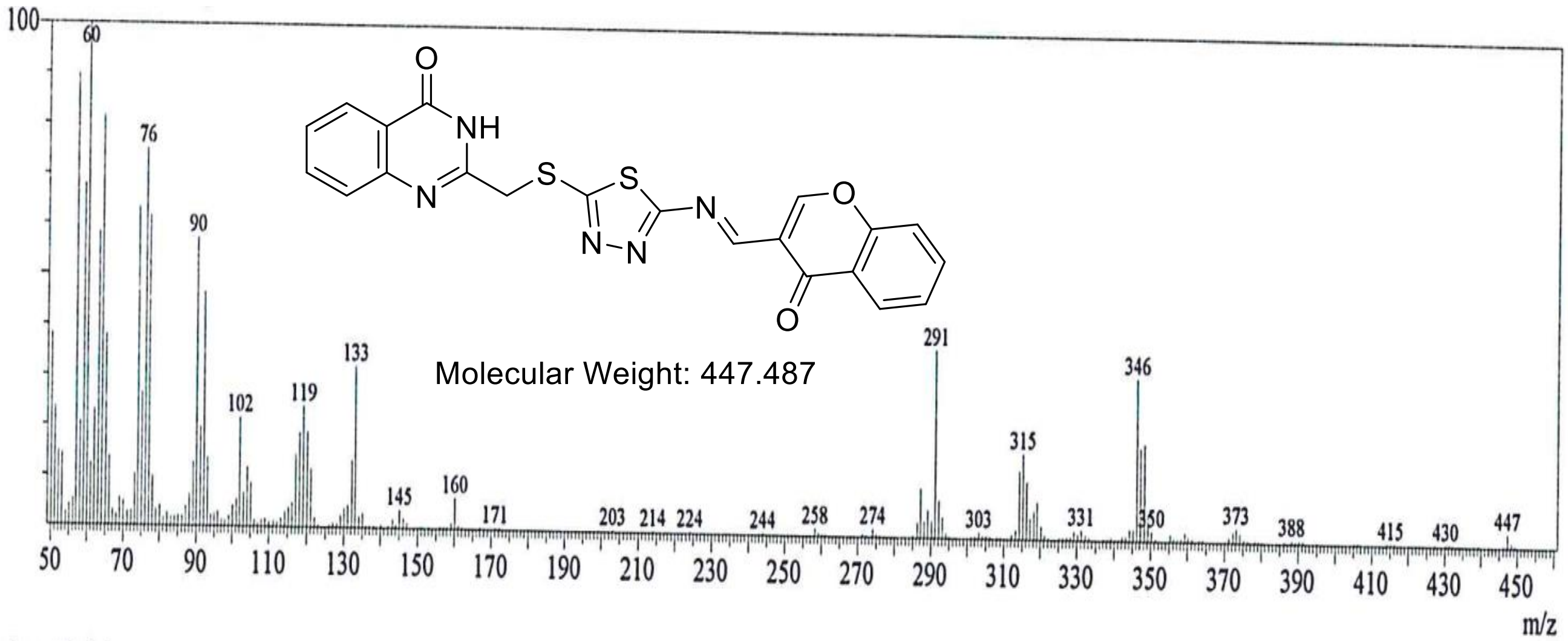

Fig. S37; Mass Spectrum of compound 13

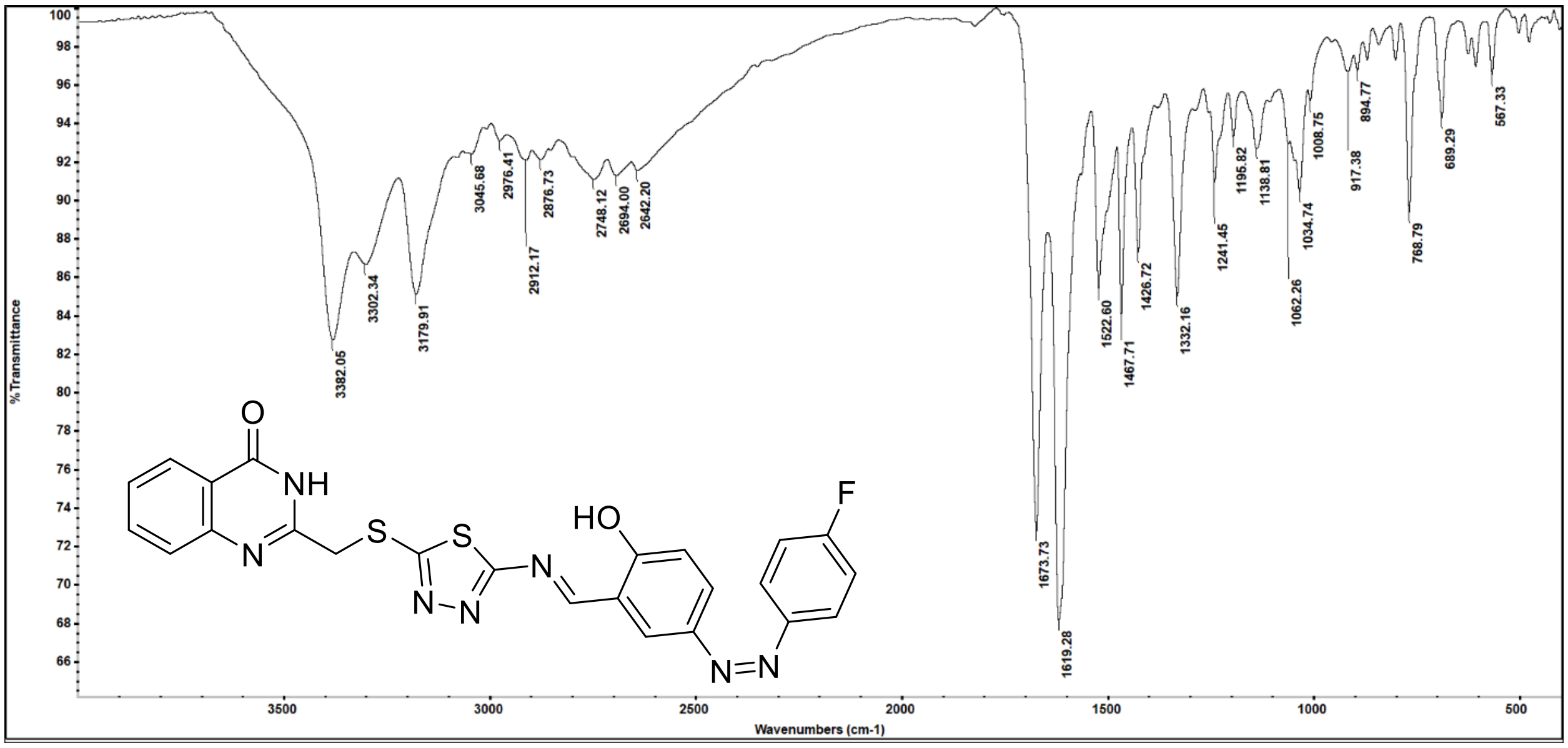

Fig.S38; FT-IR Spectrum of compound 14

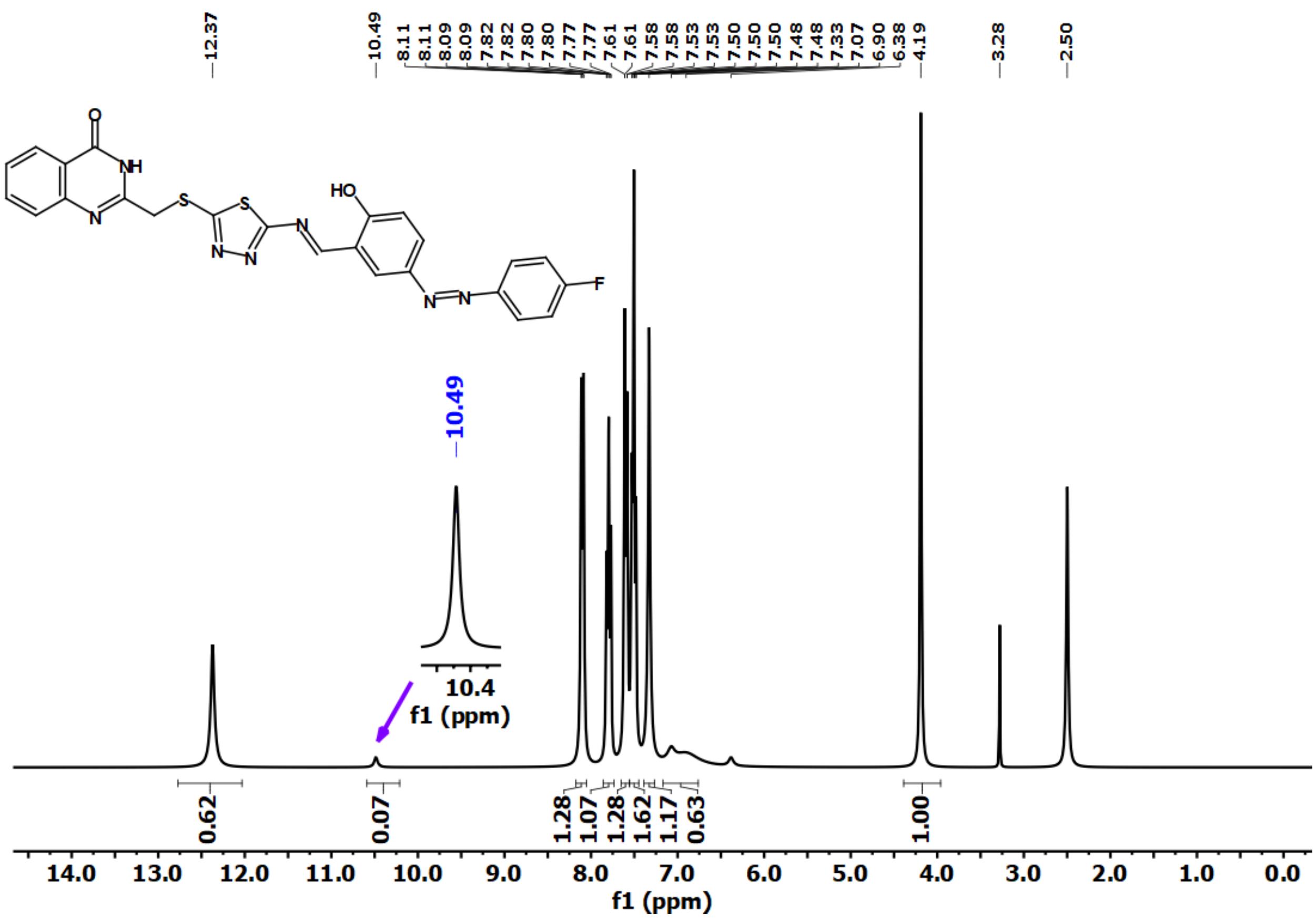

Fig. S39; ^{1}H NMR (DMSO-d_6) Spectrum of compound 14

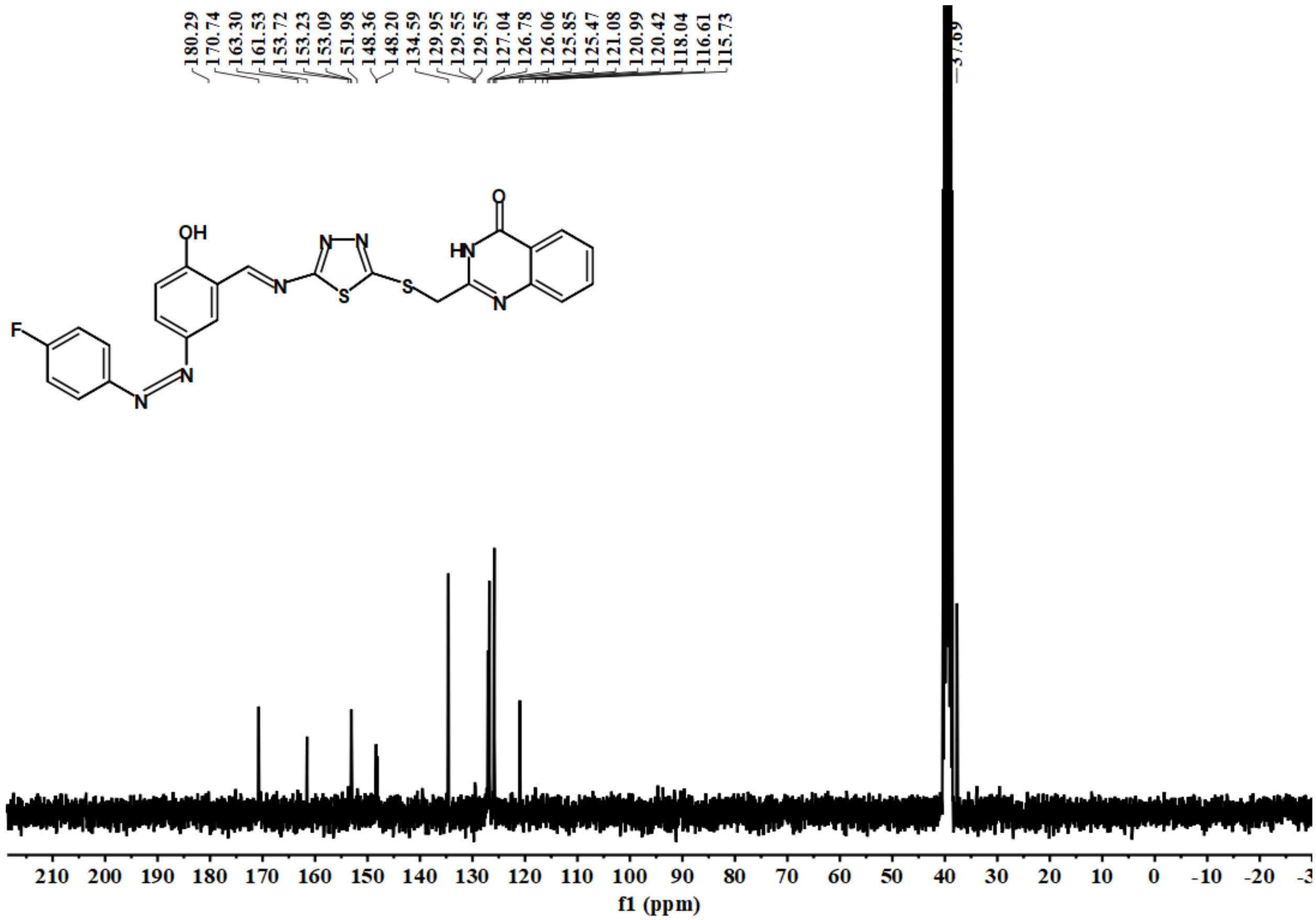

Fig. S40; ^{13}C NMR (DMSO-d_6) Spectrum of compound 14

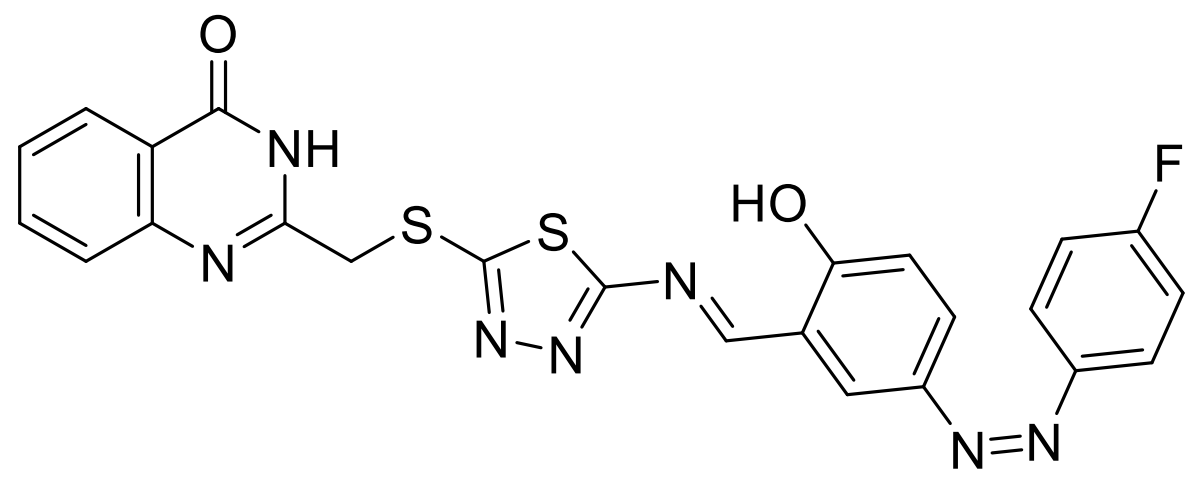

Molecular Weight: 517.557

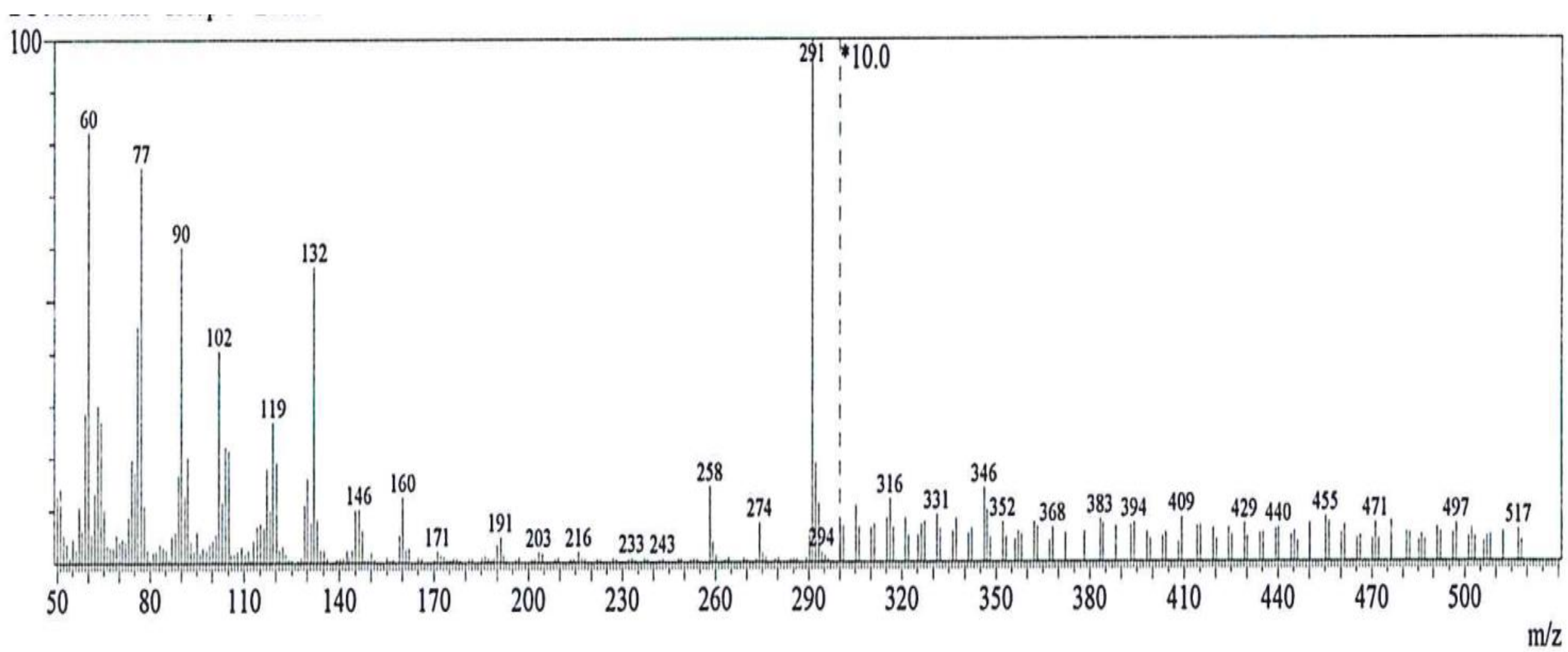

Fig. S41; Mass Spectrum of compound 14

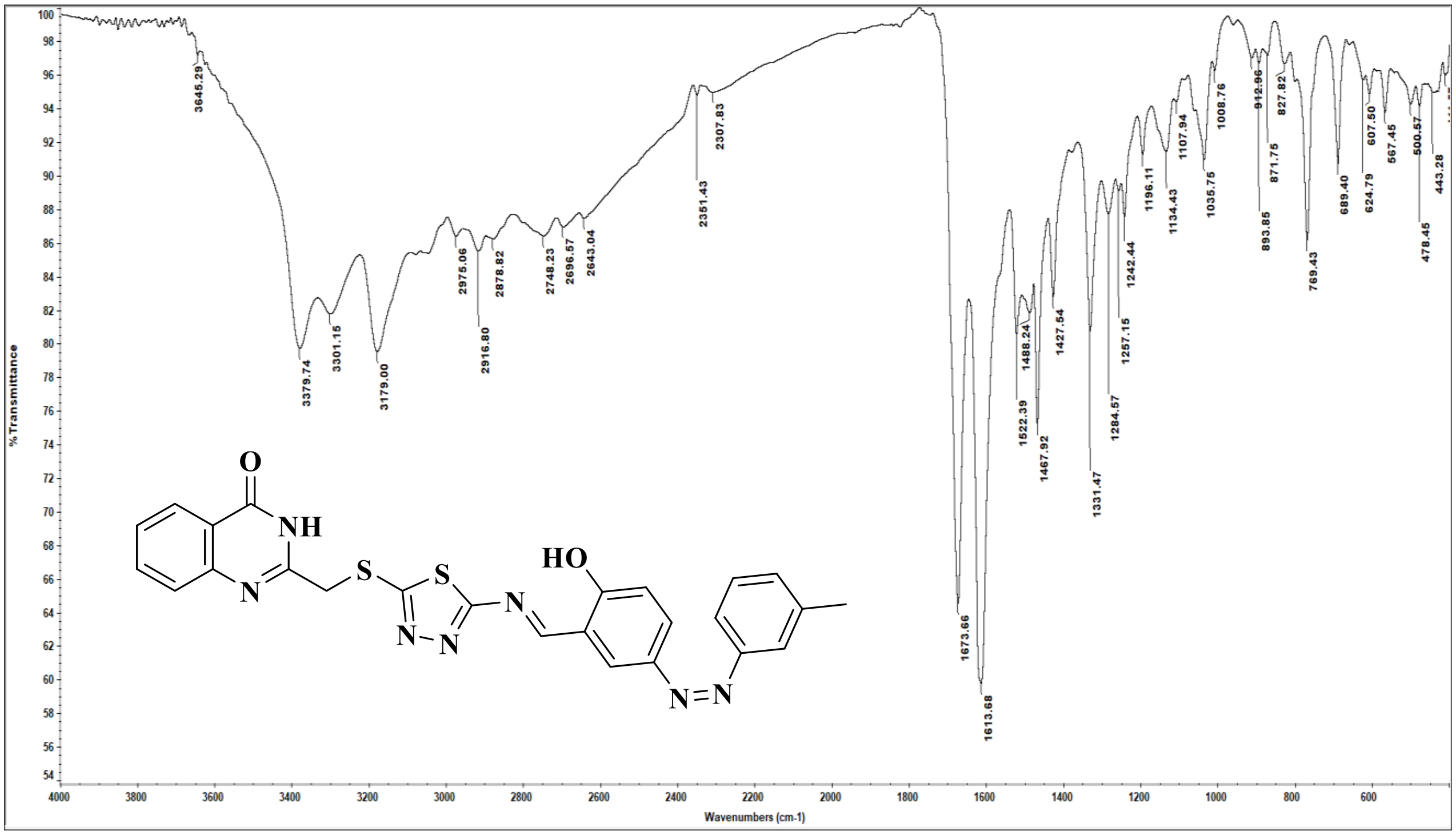

Fig.S42; FT-IR Spectrum of compound 15

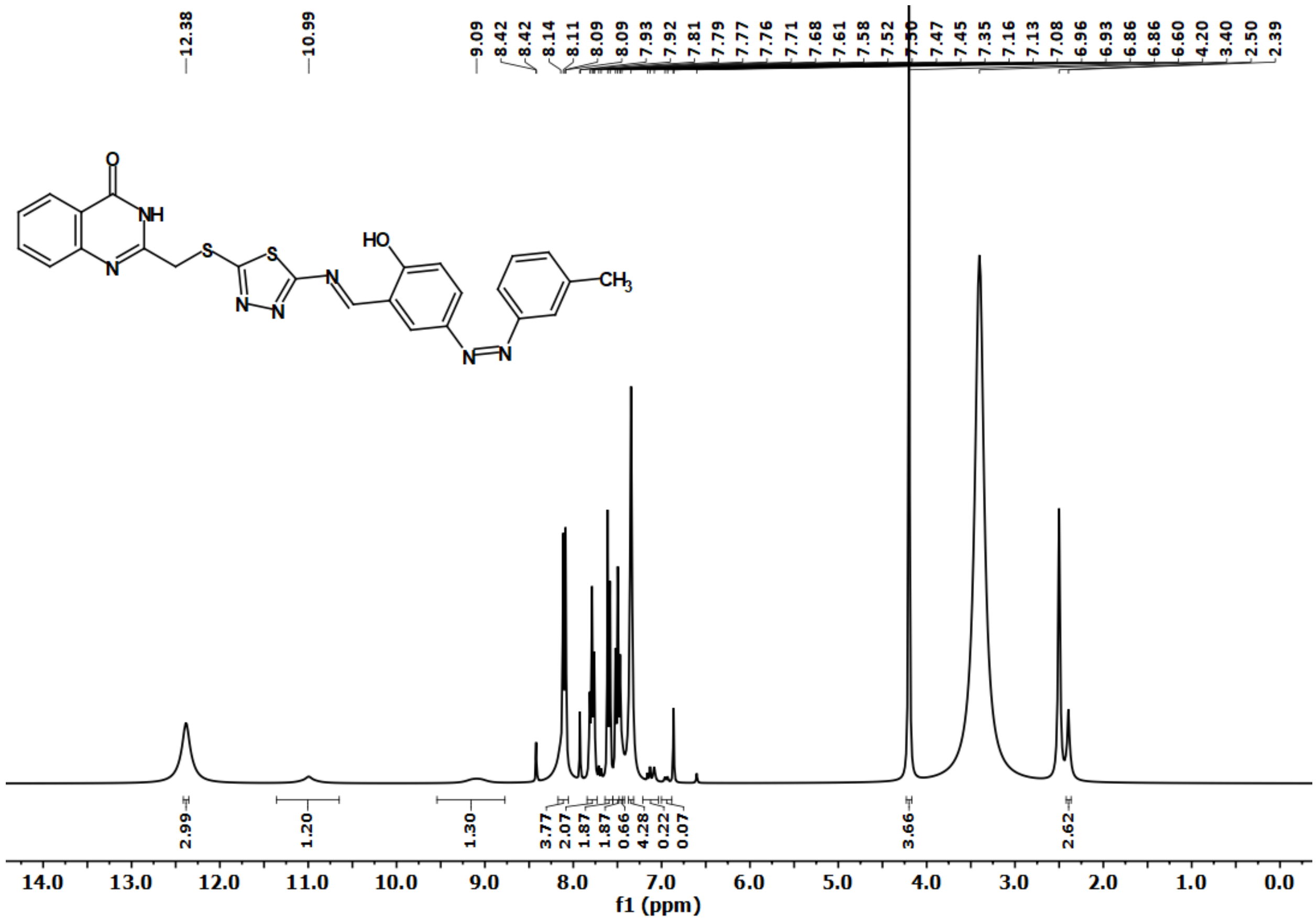

Fig. S43; ^{1}H NMR (DMSO-d_6) Spectrum of compound 15

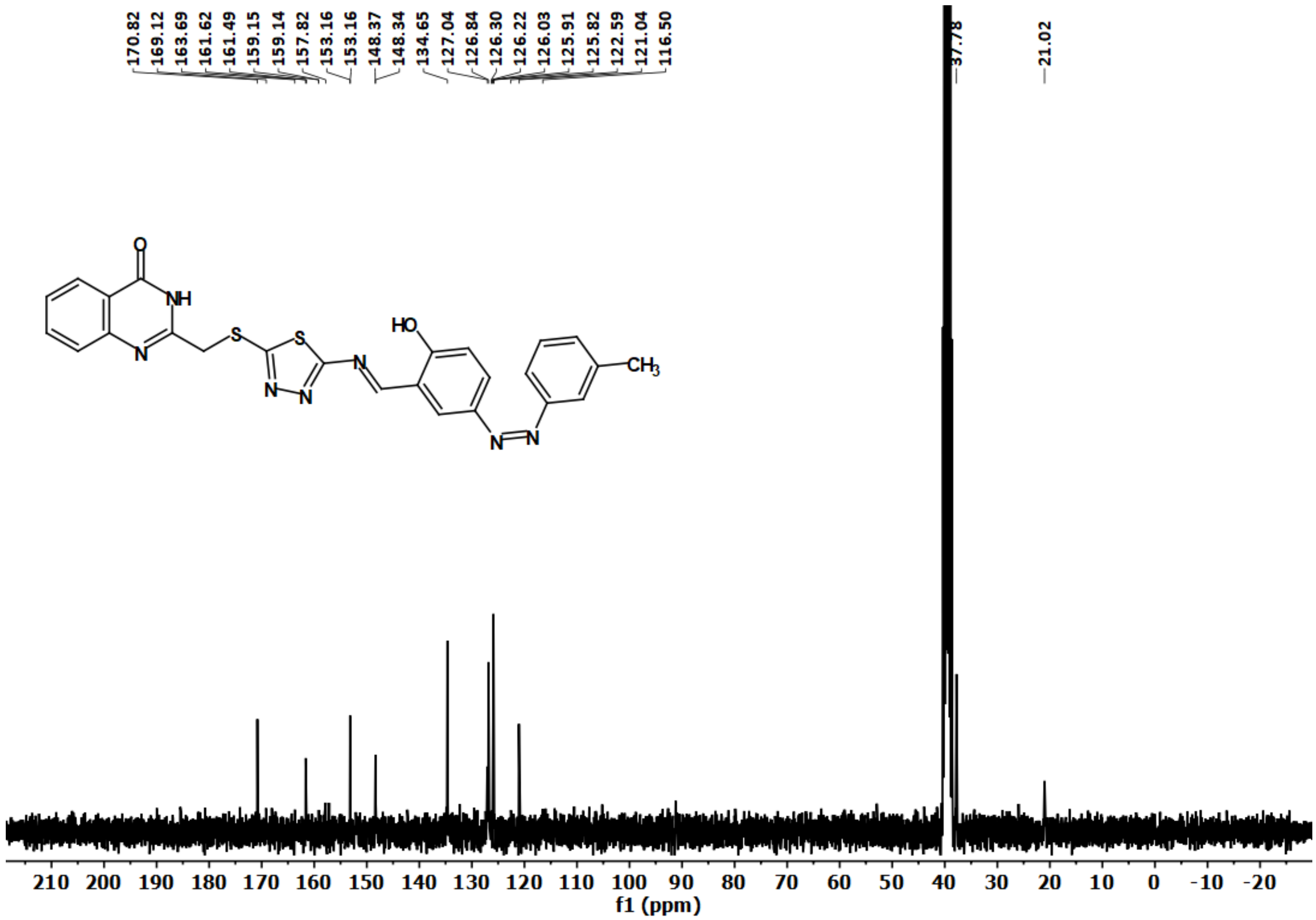

Fig. S44; ^{13}C NMR (DMSO-d_6) Spectrum of compound 15

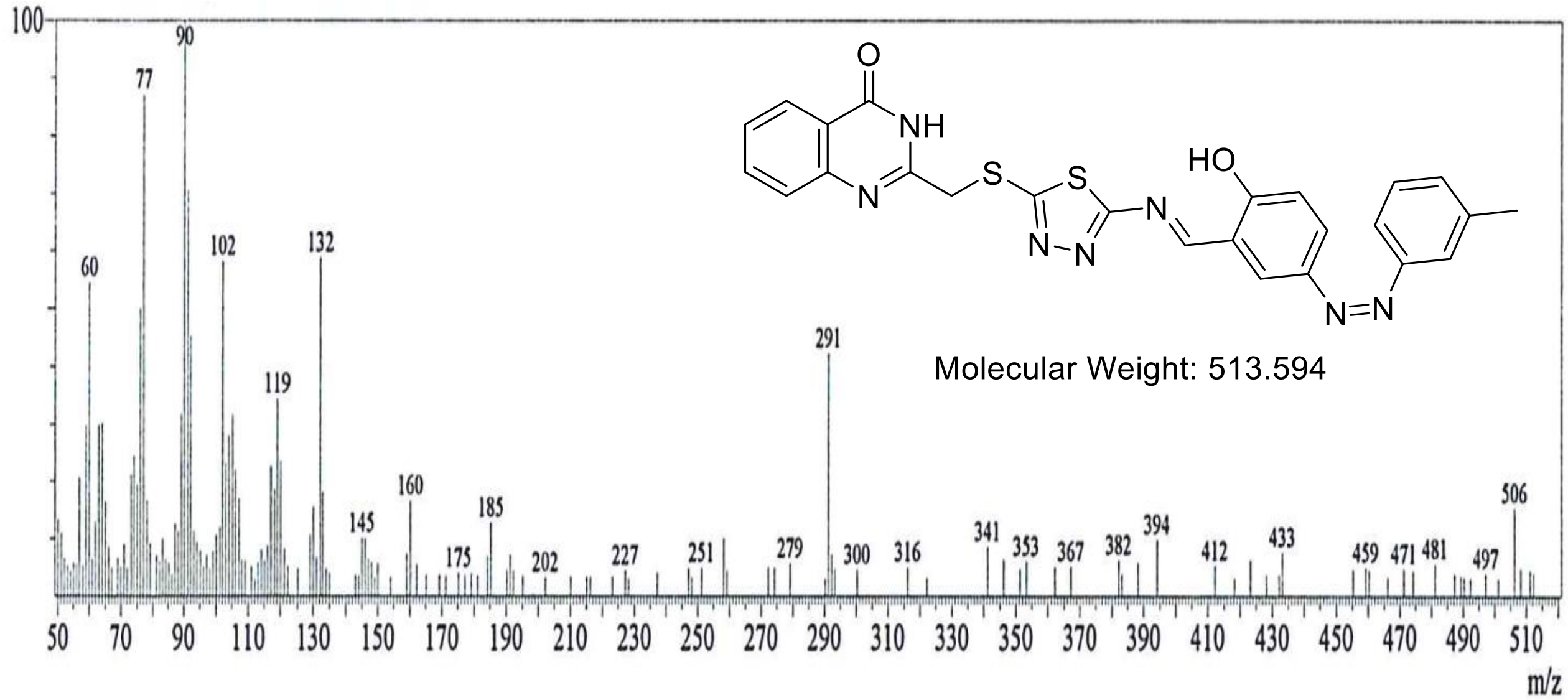

Fig. S45; Mass Spectrum of compound 15

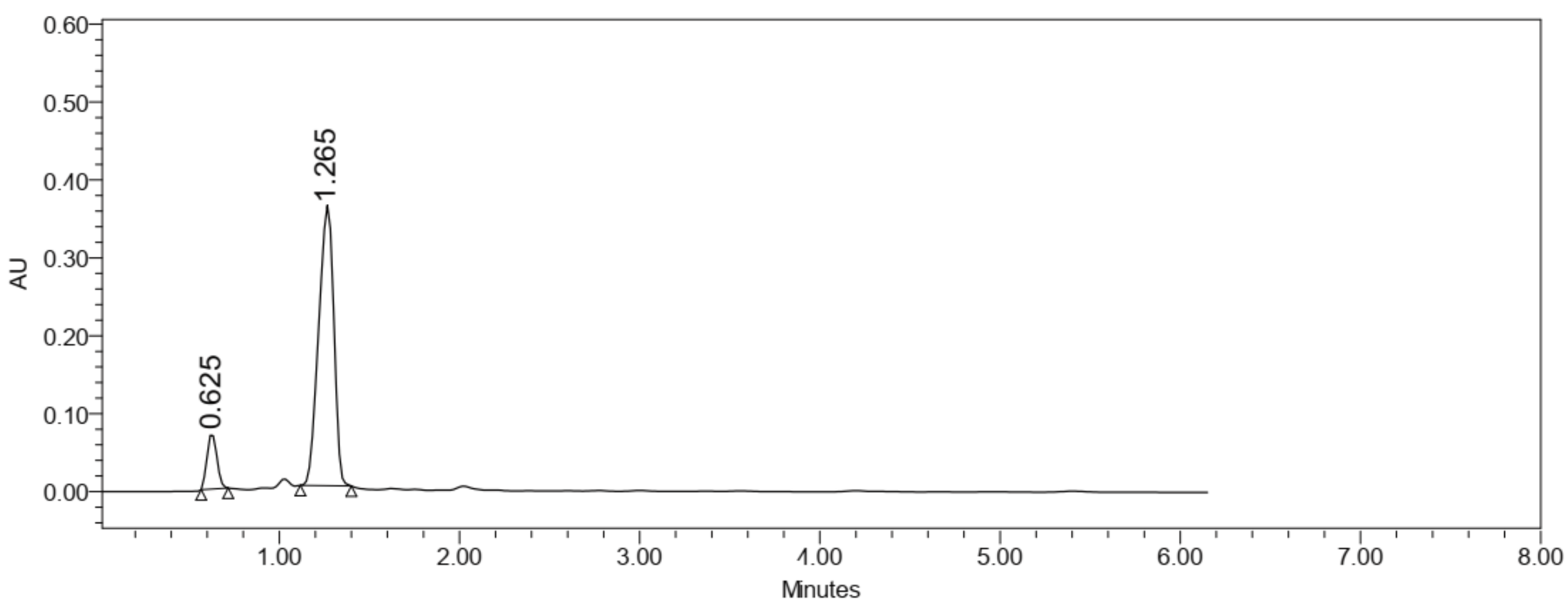

	RT	Area	% Area
1	0.625	271157	4.25
2	1.265	6107261	95.75

Fig. S46; HPLC of compound **15**

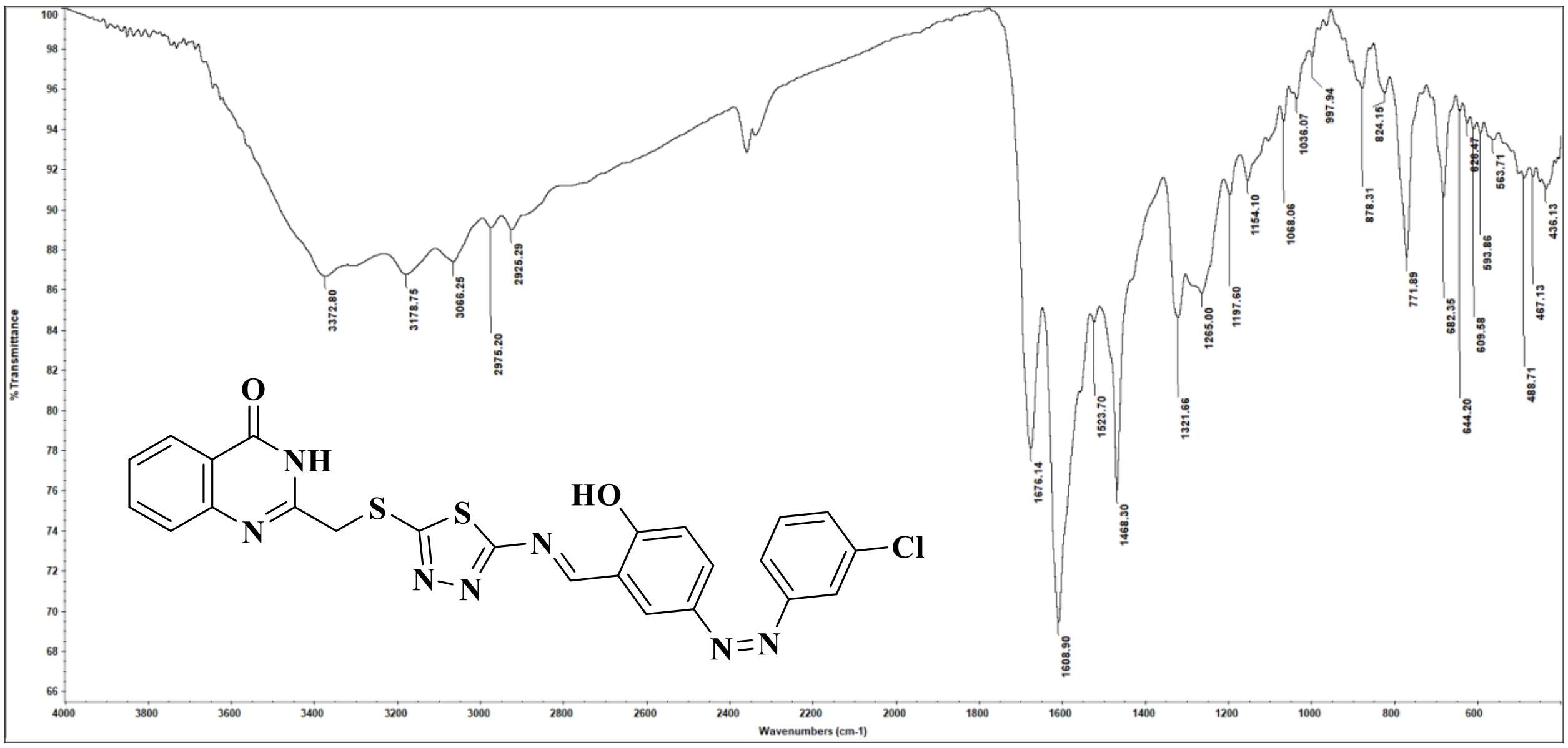

Fig.S47; FT-IR Spectrum of compound 16

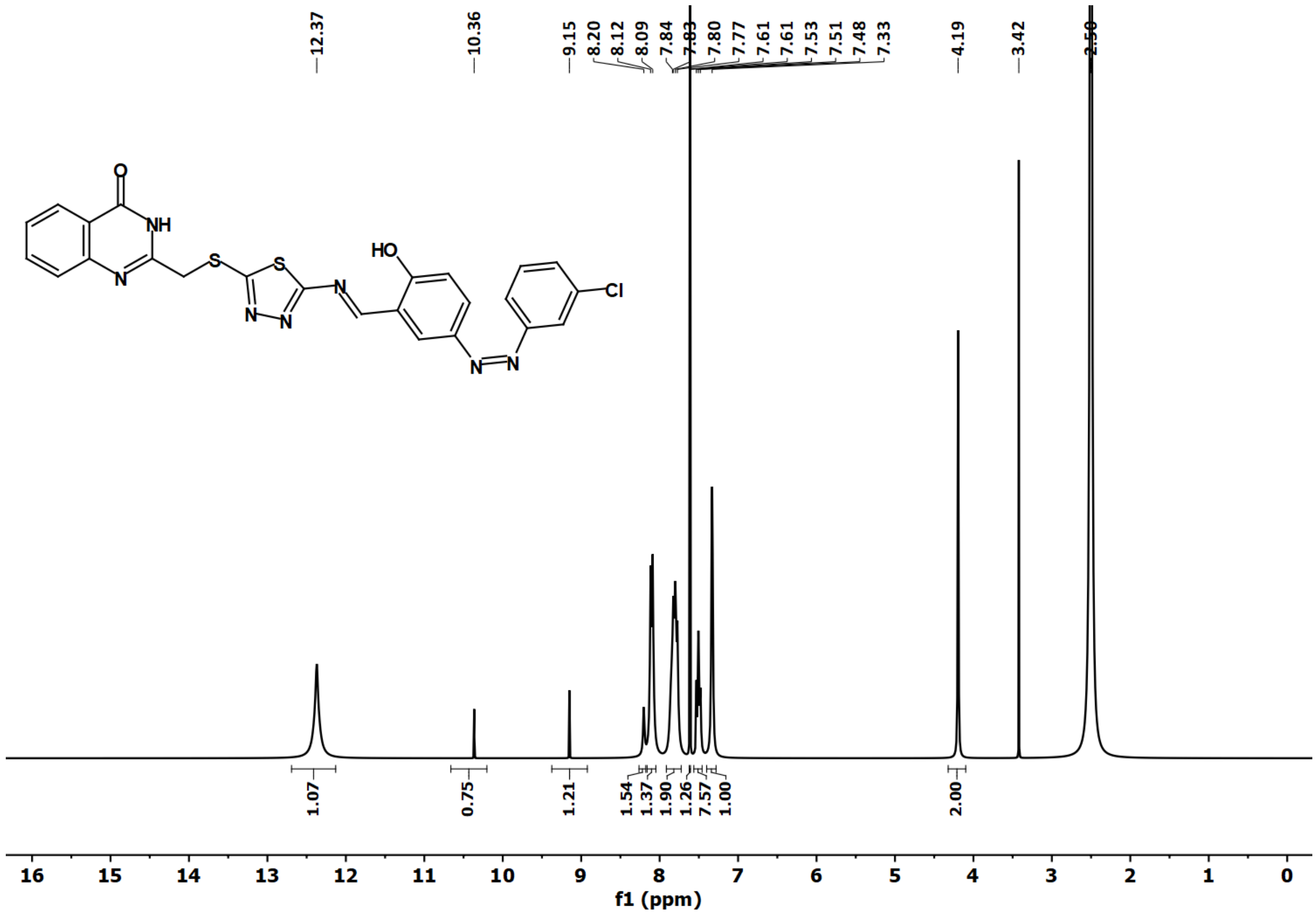

Fig. S48; ^{1}H NMR (DMSO-d_6) Spectrum of compound 16

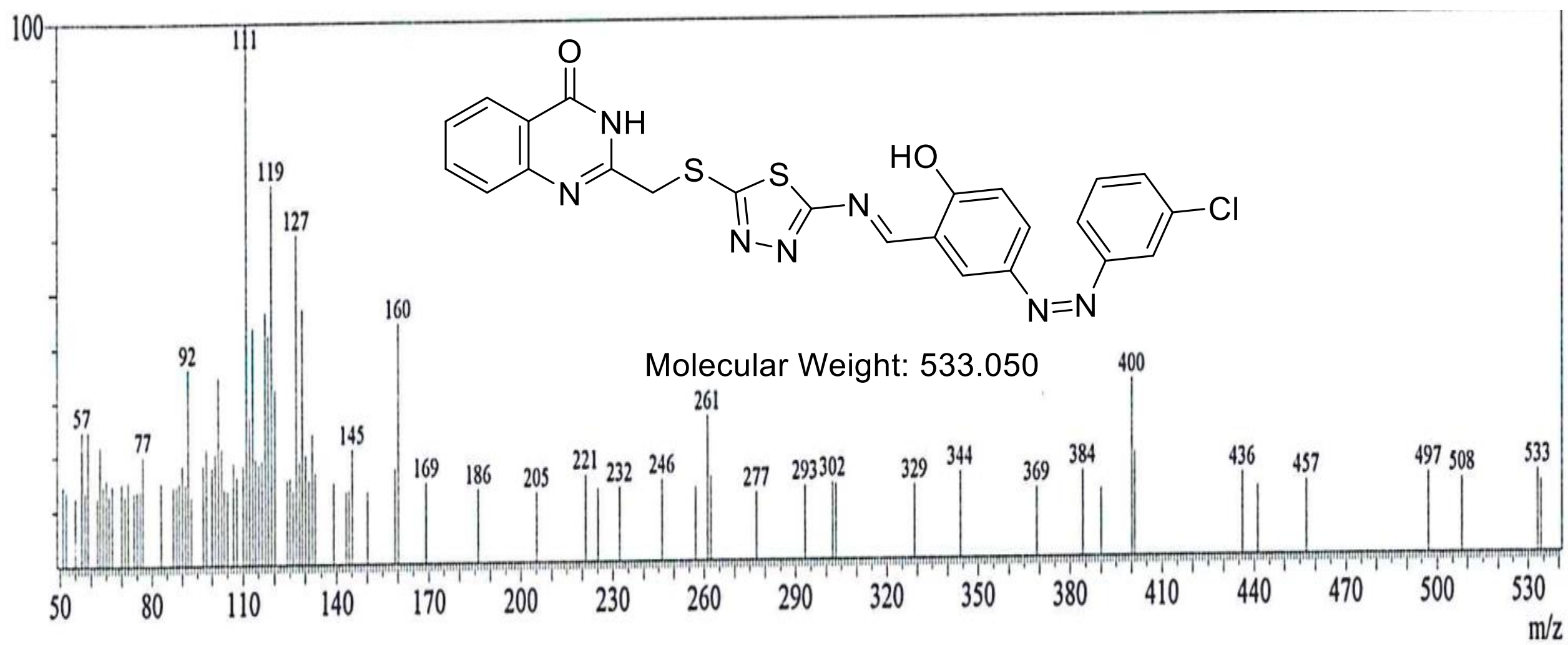

Fig. S49; Mass Spectrum of compound 16

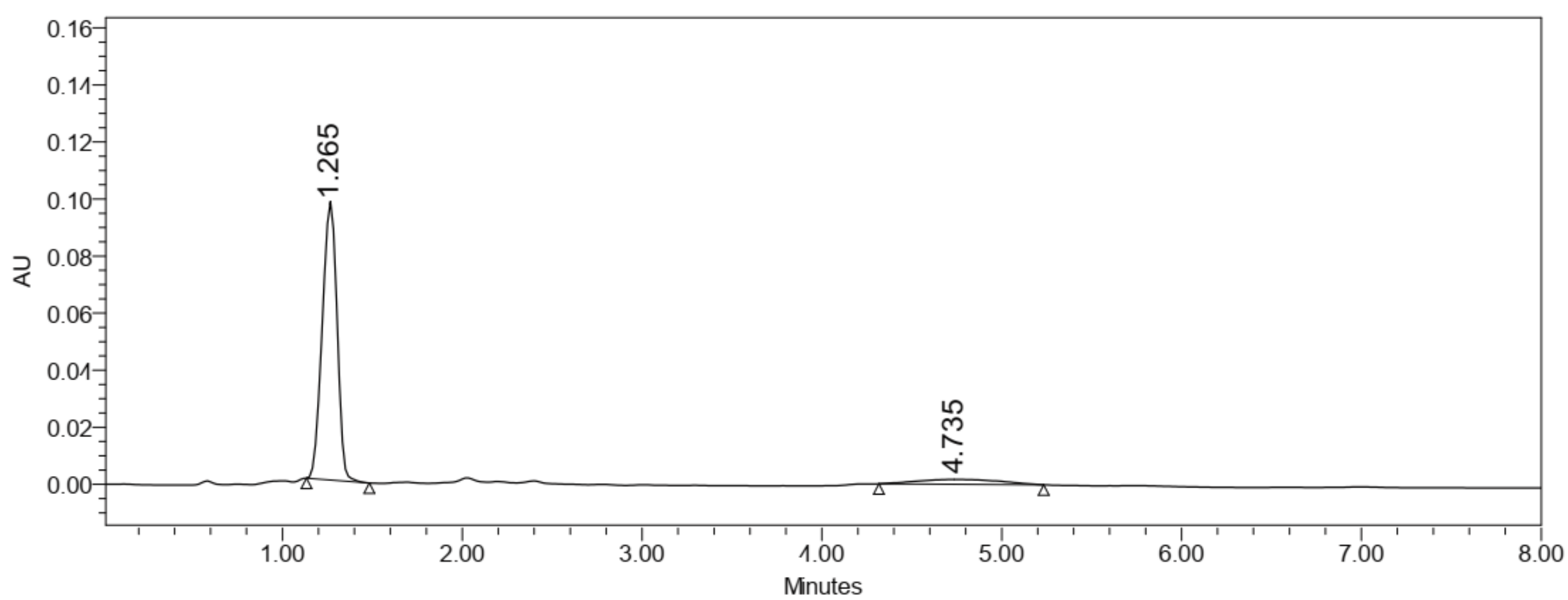

	RT	Area	% Area
1	1.265	958958	96.96
2	4.735	30007	3.04

Fig. S50; HPLC of compound **16**

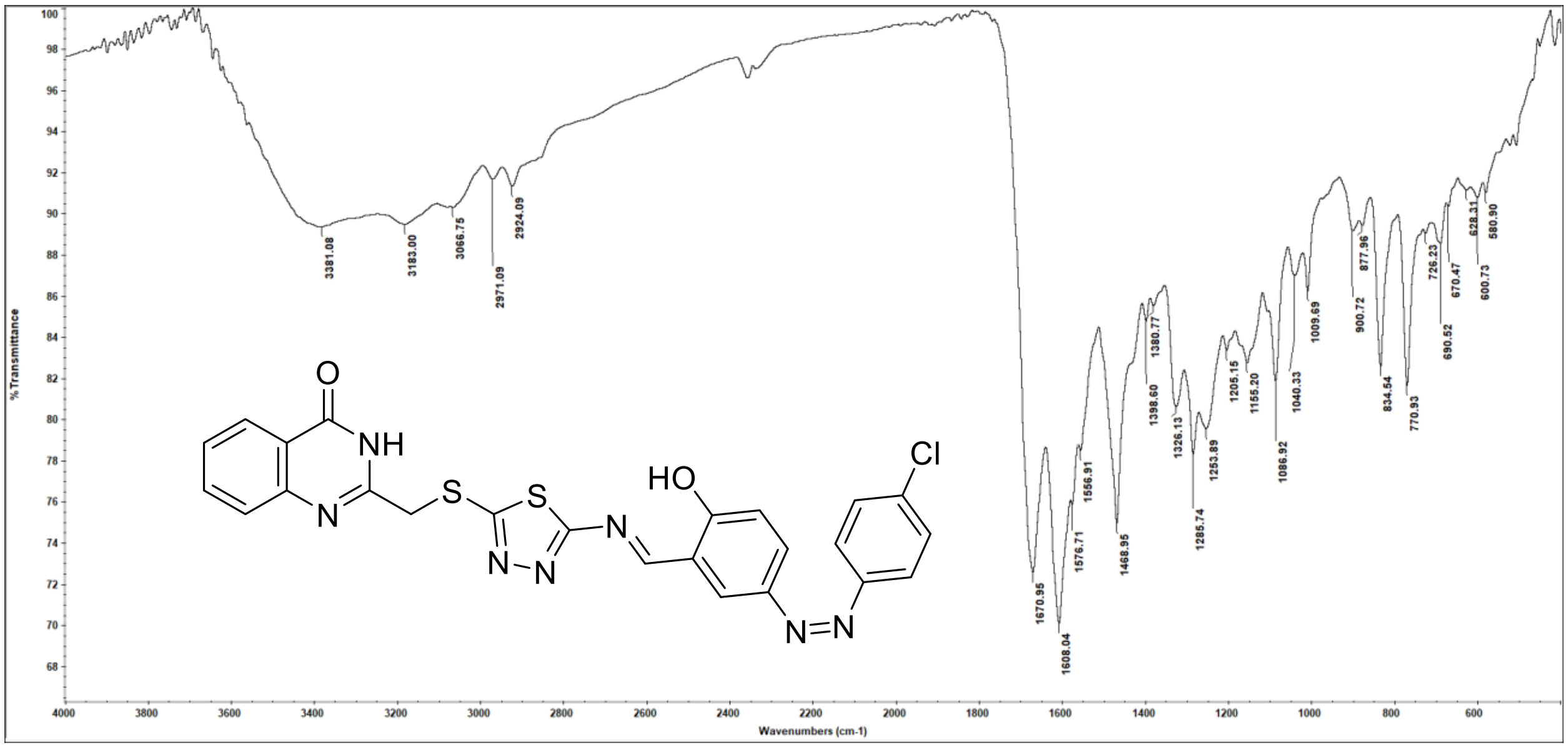

Fig.S51; FT-IR Spectrum of compound 17

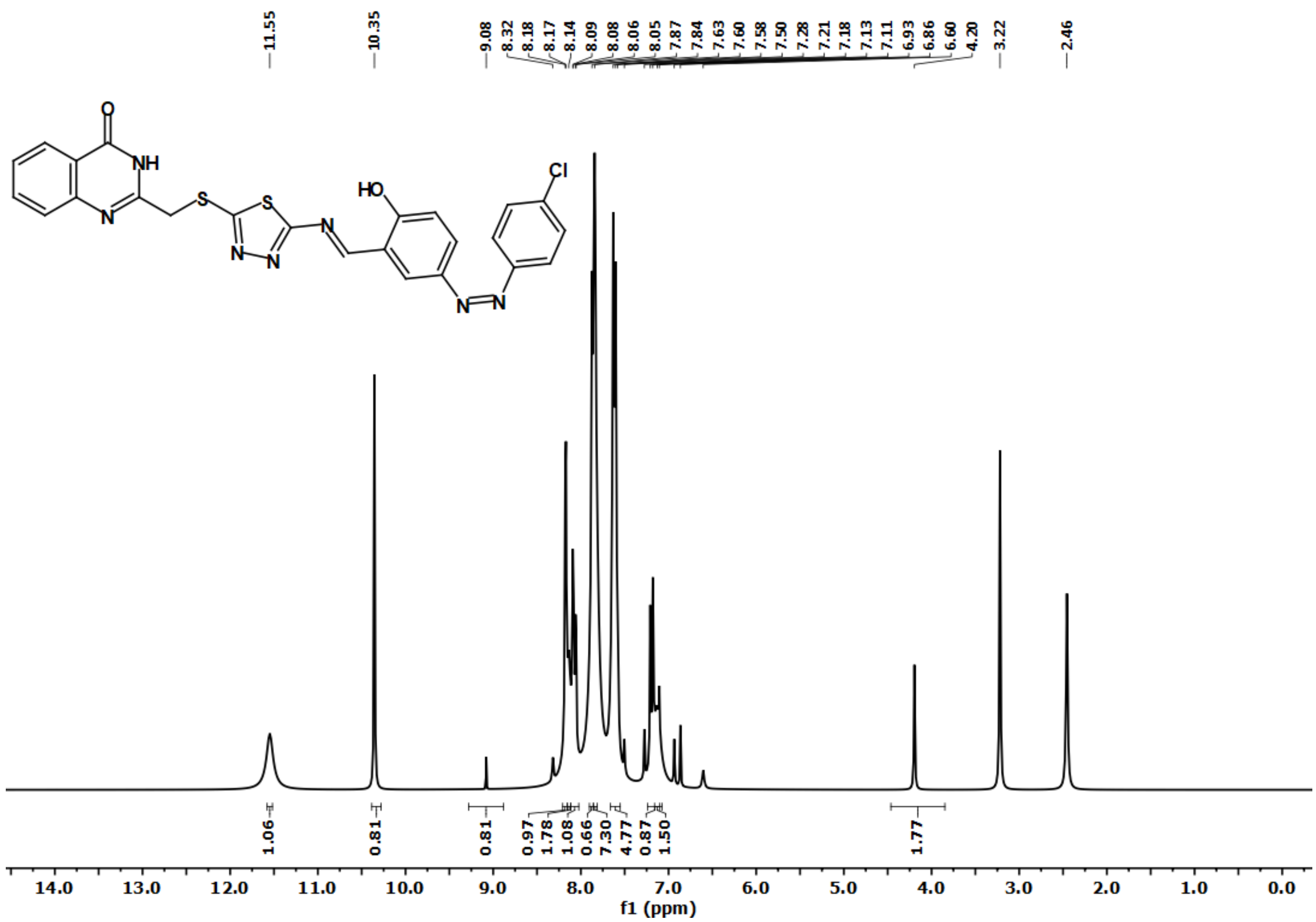

Fig. S52; ^{1}H NMR (DMSO-d_6) Spectrum of compound 17

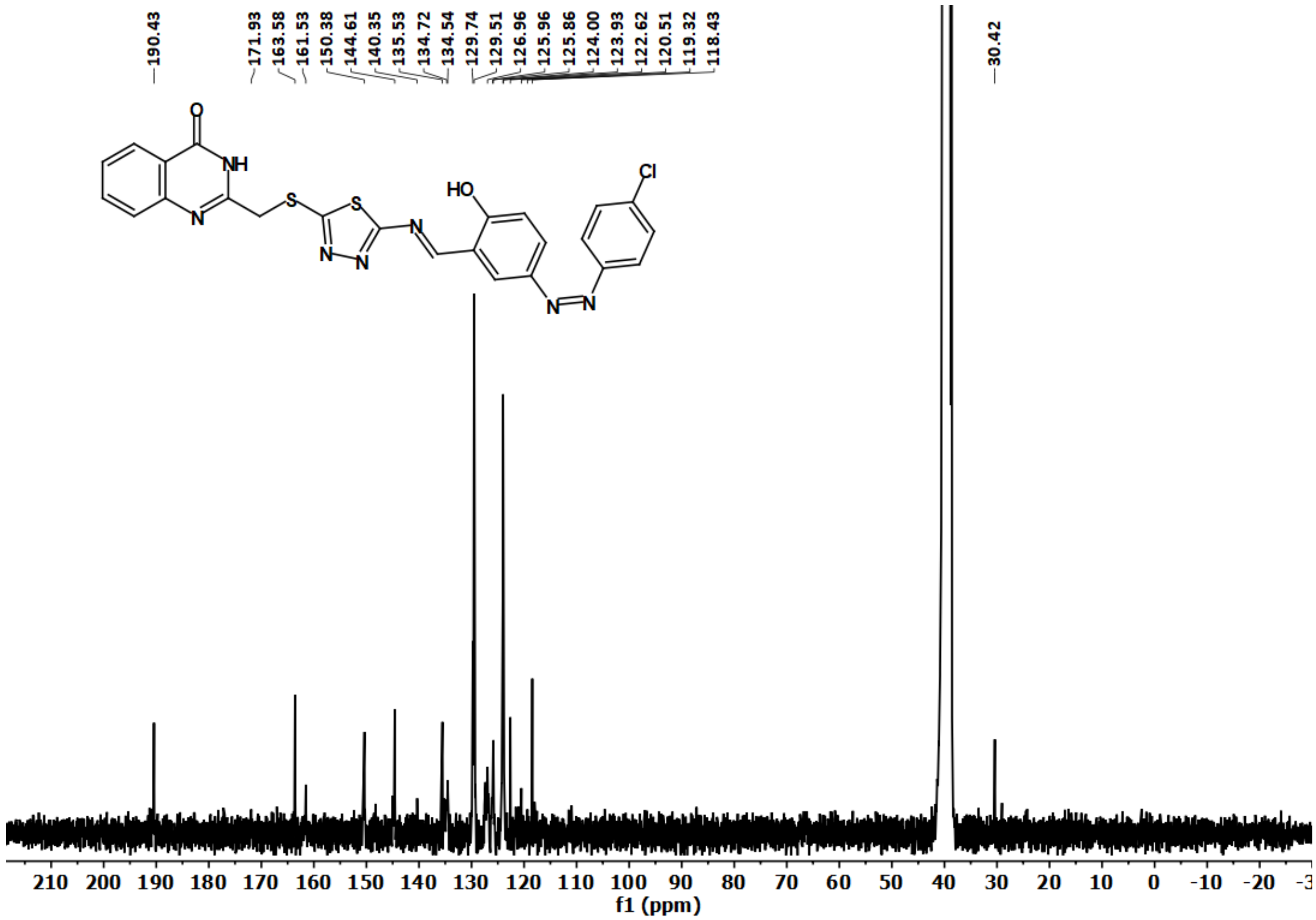

Fig. S53; ^{13}C NMR (DMSO-d_6) Spectrum of compound 17

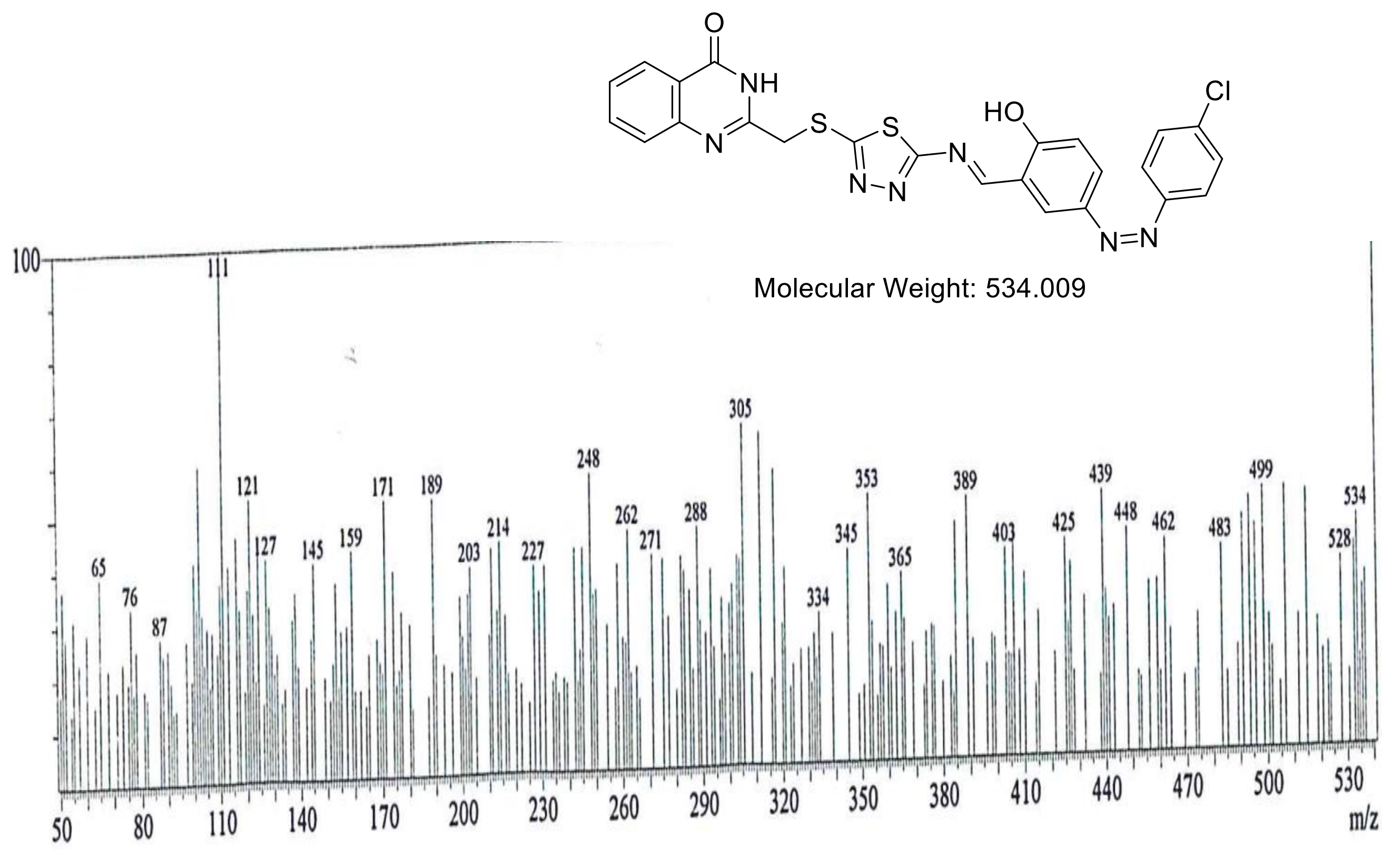

Fig. S54. Mass Spectrum of compound 17
